# The oral bacterial microbiome of occlusal surfaces in children and its association with diet and caries

**DOI:** 10.1371/journal.pone.0180621

**Published:** 2017-07-05

**Authors:** Apoena Aguiar Ribeiro, Maria Andrea Azcarate-Peril, Maria Belen Cadenas, Natasha Butz, Bruce J. Paster, Tsute Chen, Eric Bair, Roland R. Arnold

**Affiliations:** 1 Department of Pediatric Dentistry and Cariology, School of Dentistry, Fluminense Federal University, Nova Friburgo, Brazil; 2 Department of Diagnostic Sciences, School of Dentistry, University of North Carolina, Chapel Hill, United States of America; 3 Department of Cell Biology and Physiology, School of Medicine, University of North Carolina, Chapel Hill, United States of America; 4 Microbiome Core Facility, School of Medicine, University of North Carolina, Chapel Hill, United States of America; 5 Department of Microbiology, Forsyth Institute, Cambridge, United States of America; 6 Department of Oral Medicine, Infection & Immunity, Harvard School of Dental Medicine, Boston, United States of America; 7 Department of Endodontics and Biostatistics, School of Dentistry, University of North Carolina, Chapel Hill, United States of America; University of Florida, UNITED STATES

## Abstract

Dental caries is the most prevalent disease in humans globally. Efforts to control it have been invigorated by an increasing knowledge of the oral microbiome composition. This study aimed to evaluate the bacterial diversity in occlusal biofilms and its relationship with clinical surface diagnosis and dietary habits. Anamneses were recorded from thirteen 12-year-old children. Biofilm samples collected from occlusal surfaces of 46 permanent second molars were analyzed by 16S rRNA amplicon sequencing combined with the BLASTN-based search algorithm for species identification. The overall mean decayed, missing and filled surfaces modified index [DMFSm Index, including active white spot lesions (AWSL)] value was 8.77±7.47. Biofilm communities were highly polymicrobial collectively, representing 10 bacterial phyla, 25 classes, 29 orders, 58 families, 107 genera, 723 species. *Streptococcus sp_Oral_Taxon_065*, *Corynebacterium matruchotii*, *Actinomyces viscosus*, *Actinomyces sp_Oral_Taxon_175*, *Actinomyces sp_Oral_Taxon_178*, *Actinomyces sp_Oral_Taxon_877*, *Prevotella nigrescens*, *Dialister micraerophilus*, *Eubacterium_XI G 1 infirmum* were more abundant among surfaces with AWSL, and *Streptococcus gordonii*, *Streptococcus sp*.*_Oral_Taxon_058*, *Enterobacter sp*.*_str*.*_638 Streptococcus australis*, *Yersinia mollaretii*, *Enterobacter cloacae*, *Streptococcus sp*.*_Oral_Taxon_71*, *Streptococcus sp*.*_Oral_Taxon_F11*, *Centipeda sp*.*_Oral_Taxon_D18* were more abundant among sound surfaces. *Streptococcus mutans* was detected on all surfaces in all patients, while *Streptococcus sobrinus* was detected only in three patients (mean relative abundances 7.1% and 0.6%, respectively). Neither species differentiated healthy from diseased sites. Diets of nine of the subjects were scored as high in fermentable carbohydrates (≧2X/day between meals). A direct association between relative abundances of bacteria and carbohydrate consumption was observed among 18 species. High consumption of fermentable carbohydrates and sound surfaces were associated with a reduction in bacterial diversity. PCoA plots displayed differences in bacterial community profiles between sound and diseased surfaces. Our study showed that, in addition to mutans streptococci, other species may be associated with the initiation of dental caries on occlusal surfaces, and that biofilm diversity of tooth surfaces is influenced by carbohydrate consumption and a surface’s health status.

## Introduction

Dental caries remains the most common chronic disease among children aged between 5 and 17 years in the US, as well as in the world [1,2]. Recent data from CDC showed that in the US the prevalence of untreated cavities among children remains high with 19.5% in children 2–5 years of age and 22.9% in children 6–19 years of age [3]. Caries is a biofilm-mediated disease with a diverse composition of the biofilm associated with initiation and progression [4]. Different oral structures and tissues, such as tongue, teeth and gingiva, are colonized by distinct microbial communities [5,6]. Hence, to gather full information on the healthy and disease-associated oral microbiome, microbial samples should be obtained from clinically defined, discrete sites [7]. Few studies, however, have applied specific sampling for accurate characterization of the different oral microniches [6,8–10]. Moreover, most oral microbiologic studies were based on pooled samples [11–14], rather than characterizing potential differences in microbial composition between teeth and discrete sites on teeth that could influence interpretation of results.

Limited dental microbiome high-throughput DNA sequencing studies have already provided initial basic information on biofilm composition, but not the full microbial profiles of oral biofilms [15–17]. Moreover, it is now possible to compare healthy and disease-associated biofilms by high-throughput sequencing to determine the bacterial composition of the biofilm associated with formation of white-spot lesions [18,19]. Researchers are currently able to assess microbiome composition by 16S rRNA amplicon sequencing at the genus level with regard to the dominant genera; however, the capability to accurately classify reads at the species level, which is essential when comparing bacterial taxa according to caries experience, has been limited. Recently, Al-Hebshi *et al*. [20] developed a BLASTN-based search algorithm that uses three 16S rRNA reference sequence databases (HOMD version 13.2, HOMDextended version 1.1 and Greengene Gold) for classification of next-generation sequencing (NGS) reads from oral microbiological samples to the species level.

It is clear that dental caries is the result of dissolution of the tooth mineral by a reduction in pH due to the sustained fermentation of carbohydrate by bacteria in a local biofilm structure that limits the ability of saliva to wash away or buffer the acid metabolic products [21,22]. Cariogenic species must be able not only to produce acid, but also to sustain metabolism in a low pH environment. There are also species that utilize these acid end products to meet their own metabolic needs preventing the critical drop in pH associated with demineralization. A better understanding of the nature of species that can persist and thrive in the hostile environment of a potentially cariogenic biofilm is therefore important for caries risk evaluation and for development of caries preventive and control strategies. Our study aimed to define the diversity of bacterial microbiomes from newly erupted occlusal surfaces and compare it with clinical surface diagnosis [sound versus diseased (AWSL)], dietary habits and fluoride exposure, by 16S rRNA amplicon sequencing combined with the BLASTN-based search algorithm for species identification.

## Materials and methods

### Study design and subjects

We conducted a cross-sectional comparison of microbial biodiversity in a convenience sample of 13 children, aged 12 years old, both genders (7 girls/6 boys), students of public schools from Nova Friburgo, Rio de Janeiro State, Brazil. The Ethics Committee of HUAP/Fluminense Federal University approved this study and written informed consent was obtained from all parents/guardians. The inclusion criteria were: (1) medically healthy child; (2) no use of antibiotics within the last 3 months. All children/parent pairs were interviewed through a validated semi-quantitative food-frequency questionnaire (QFASQ) to access dietary habits [23], in terms of frequency of fermentable carbohydrates in separate eating events (excluding breakfast, lunch and dinner) and their sugars and starch content, including intake of soft drink, juice and snacks [24]. Dietary groups were classified as: Low–consumption ≤ 1 time; High–consumption ≥ 2 times; between main meals (S1 Fig).

### Clinical examinations and biofilm collection

All children were examined using a standardized clinical protocol and a single examiner (AAR). Children were asked to refrain from brushing in the morning prior to sampling. Clinical appointments consisted of: (1) performance of Biofilm Thickness Index [25]; (2) collection of dental biofilm from all fully erupted occlusal surfaces, of all fully or partially erupted second permanent molars; (3) supervised tooth brushing and (4) dental examination, according to Nyvad *et al*. [26]. Caries prevalence scores in permanent dentition (DMFT-m—decayed, missing, filled teeth, DMFS-m—decayed, missing, filled surface and SiC—'Significant Caries Index—mean DMFT of the one third of the study group with the highest caries score) were analyzed considering active white spot lesion (AWSL) as caries [26]. Tooth eruption stage was classified as “total” when the tooth had reached the occlusal plane on the arch; and as “partial” when the tooth had not reach the occlusal plane on the arch. After examination, all children were enrolled for treatment and follow-up in the Dental Clinic from Fluminense Federal University–Nova Friburgo, Brazil.

### Sample collection

Biofilm samples were collected 2 h after eating in the morning, according to the Manual of Procedures for Human Microbiome Project (http://hmpdacc.org/resources/tools_protocols.php), with minor modifications. Briefly, each second molar was isolated with cotton rolls and dried with a gentle air stream to avoid saliva contamination. Then, supragingival plaque from each occlusal surface was removed with sterile toothpicks and immediately inserted in separate Eppendorf tubes containing 1ml of transport media (Anaerobic Dental Transport Medium; Anaerobe Systems®) and sent to the laboratory. The samples were maintained refrigerated until analysis.

### DNA isolation and 16s rRNA amplicon library preparation and sequencing

DNA isolation, preparation of sequencing libraries and sequencing were done in the UNC Microbiome Core Facility as described [27,28]. Briefly, bacterial DNA extraction was performed using QIAmp DNA extraction kit (QIAGEN). A step of pre-incubation with lysozyme for 30 min was introduced to the protocol to ensure optimal DNA yield from Gram-positive bacteria. For generation of sequencing libraries, 12.5ng of total DNA from each sample was amplified using the 2x KAPA HiFi HotStart ReadyMix (KAPA Biosystems, Wilmington, MA). Primers targeting the V1–V2 region of the 16S rRNA gene [29,30] were designed to incorporate Illumina compatible sequencing adaptors. The complete sequences of the primers were: F–5’*TCGTCGGCAGCGTCAGATGTGTATAAGAGACAGAGAGTTTGATCCTGGCTCAG*3’ and R–5’*GTCTCGTGGGCTCGGAGATGTGTATAAGAGACAGGCTGCCTCCCGTAGGAGT*3’. PCR conditions consisted of an initial denaturing step at 95° for 3 min, 25 cycles of 95°C for 30 sec, 55°C for 30 sec and 72°C for 30 sec, followed by extension at 72°C for 5 min and a final hold at 4°C. Illumina sequencing adapters and dual index barcodes (Illumina, San Diego, CA) were added using one more round of PCR amplification consisting of 8 cycles. PCR products were purified using AMPure XP reagent (Beckman Coulter, Indianapolis, IN), quantified by Quanti-IT Picogreen dsDNA 1 kit (Invitrogen) and pooled in equimolar amounts. Sequencing was performed on a MiSeq instrument (Illumina) operating Real Time Analysis software version 1.17.28. Paired-end sequencing used custom primers and a 500-cycle sequencing kit (version 3) according to manufacturer instructions. Amplicon sequencing was carried out in the presence of 7% PhiX control (Illumina) to allow proper focusing and matrix calculations.

### Bioinformatics pipeline and statistical analysis

Raw reads were de-multiplexed and quality filtered. All processed unique reads where submitted to BLASTN search against 16S rDNA, using the BLASTN parameters: -q-5-r4-G5-E5. The BLASTN results were parsed using the following criteria: (a) for each read, the alignment length must be > = 90% of read length; (b) for each read, the best hit to references was determined by highest percent identity and score (reflecting alignment length); (c) if a read hit multiple reference sequences that represent multiple species with equal percent identity and score, all the species were recorded in the original results. A consensus taxonomy level for these multiple species would be determined. Read count data from the 98% cutoff were used to calculate combined counts at different taxonomy levels and percent read counts by sample were used to chart the stack-column graphs [20]. Observed species richness, Chao1 and Shannon's index were recorded and compared at the 10,000 rarefactions depth. Phylogenetic and non-phylogenetic beta diversity matrices were calculated by three-dimensional Principal Coordinate Analysis (PCoA) plots, calculated within QIIME [31,32] using weighted and unweighted UniFrac distances between samples. For each sample group, descriptive statistics included means and standard deviations (SD). Significance tests for differences in the multivariate structure of microbiome communities between patients and surface clinical diagnosis were performed using mixed effects regression models. Bacterial abundance was the outcome variable, and fixed effect covariates included dummy variables for tooth group (healthy vs. AWSL), diet, gender, and tooth. A random effect term for each participant was also included in the model. The value of the coefficients (and associated standard errors) for tooth group and diet were calculated as an estimate of the mean difference in bacterial abundance between the groups. The null hypothesis of no association between bacterial abundance and AWSL/diet was evaluated by testing the null hypothesis that the coefficient for AWSL/diet was equal to 0. Similar mixed models were used to evaluate the association between these groups and diversity measures. In these models, the diversity measure was the outcome variable, and fixed effect covariates included dummy variables for tooth group, diet, gender, and tooth. However, since multiple diversity measures were collected from each sample, these models included random effects for both the subject and the sample. P-values ≤0.05 were considered statistically significant. The sequencing data were submitted to https://www.ncbi.nlm.nih.gov/Traces/study/?acc=SRP100199.

## Results

### Sample description

A total of 46 occlusal surfaces were analyzed. S1 Table shows the distribution of samples according to dietary habits, fluoride exposure and oral health status: 22 molars were sound and 24 molars had AWSL on the occlusal surface. We observed a high accumulation of biofilm with 9 out of the 13 children, scoring 5 in the Biofilm Thickness Index [23]. Mean DMFT-m values were 6.31±4.25; DMFS-m values were 8.31±7.15 and mean SiC was 11.75. Most subjects (n = 9) consumed between meal fermentable carbohydrates (sugars and starch) in high frequency (≧2X/day). The main source of fluoride was toothpaste since there was no regular fluoridation of tap water in the city.

### Microbiome composition of plaque samples from occlusal surfaces

A total of 9,589,418 sequences were generated from the 46 occlusal biofilm samples, and 7,868,089 reads matched 723 unique species (171,045±78,732 reads per sample). Amplicon reads were assigned to 10 bacterial phyla, 25 classes, 29 orders, 58 families, 107 genera and 723 species. S2 Table shows the sample distribution by patient and tooth, according to the number of sequences obtained. S1 Fig illustrates bacterial relative abundances in all taxa levels from Phylum to Species.

**Fig 1:**
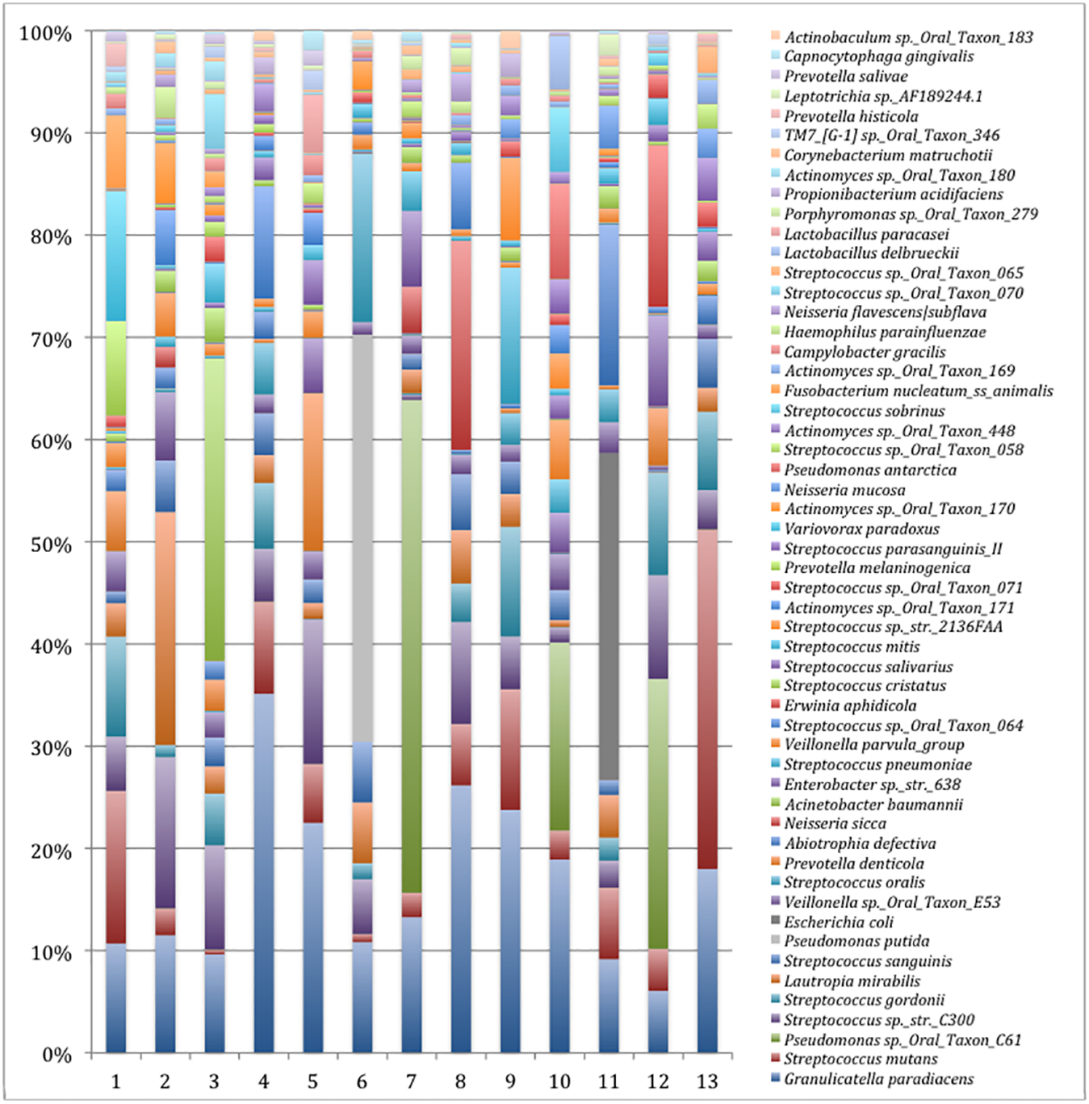
Total distribution of bacterial taxa per patient at the species level, representing a high interindividual variation. For illustrative purposes, only the 55 more abundant species are represented. The total representation per sample was 90%.

As would be expected, high interindividual variation was observed. Of the total of 723 species discerned, only 11 species were found in all subjects, but not necessarily on all surfaces within a subject: *Granulicatella paradiacens* (mean relative abundance 15.1% ± 7.8; variability ranging from 5.5–33.2%); *Streptococcus mutans* (7.1% ± 7.8; variability ranging from 0.4–31.1%); *Streptococcus sp*._str._C300 (6.0% ± 3.7; variability ranging from 0.4–13.1%); *Streptococcus gordonii* (4.1% ± 3.5; variability ranging from 0.0–10.2%); *Streptococcus sanguinis* (2.9% ± 1.6; variability ranging from 0.2–5.5); *Veillonella sp*._Oral_Taxon_E53 (2.2% ± 1.3; variability ranging from 0.3–5.9); *Abiotrophia defective* (2.0% ± 3.6; variability ranging from 0.0–14.1); *Veillonella parvula*_group (1.6% ± 1.4; variability ranging from 0.1–5.4); *Streptococcus cristatus* (1.1% ± 0.75; variability ranging from 0.1–2.6%); *Streptococcus mitis* (1.0% ± 0.79; variability ranging from 0.2–2.9) and *Actinomyces sp*.*_Oral_Taxon_169* (0.5% ± 0.5; variability ranging from 0.0–2.1%). Fig 1 illustrates the total distribution of bacterial taxonomy per patient at the species level, and S3 and S4 Tables show the overall most abundant species (relative abundance ≧ 0.1) representing over 98% of the total sample by patient and by sample, respectively.

Intra-individual variation was not observed between species diversity, but among species abundances, in relation to surface oral health. For example, in each patient, biofilm from sound surfaces showed highest abundances of *Granulicatella paradiacens*, *Streptococcus sp*.*_str*.*_C300* and *Streptococcus sp*.*_Oral_Taxon_064*, while *Veillonella sp*.*_Oral_Taxon_E53* was highly abundant on surfaces with AWSL. The differences of species’ abundance by each surface and caries diagnosis are presented in S4 Table.

**Fig 2:**
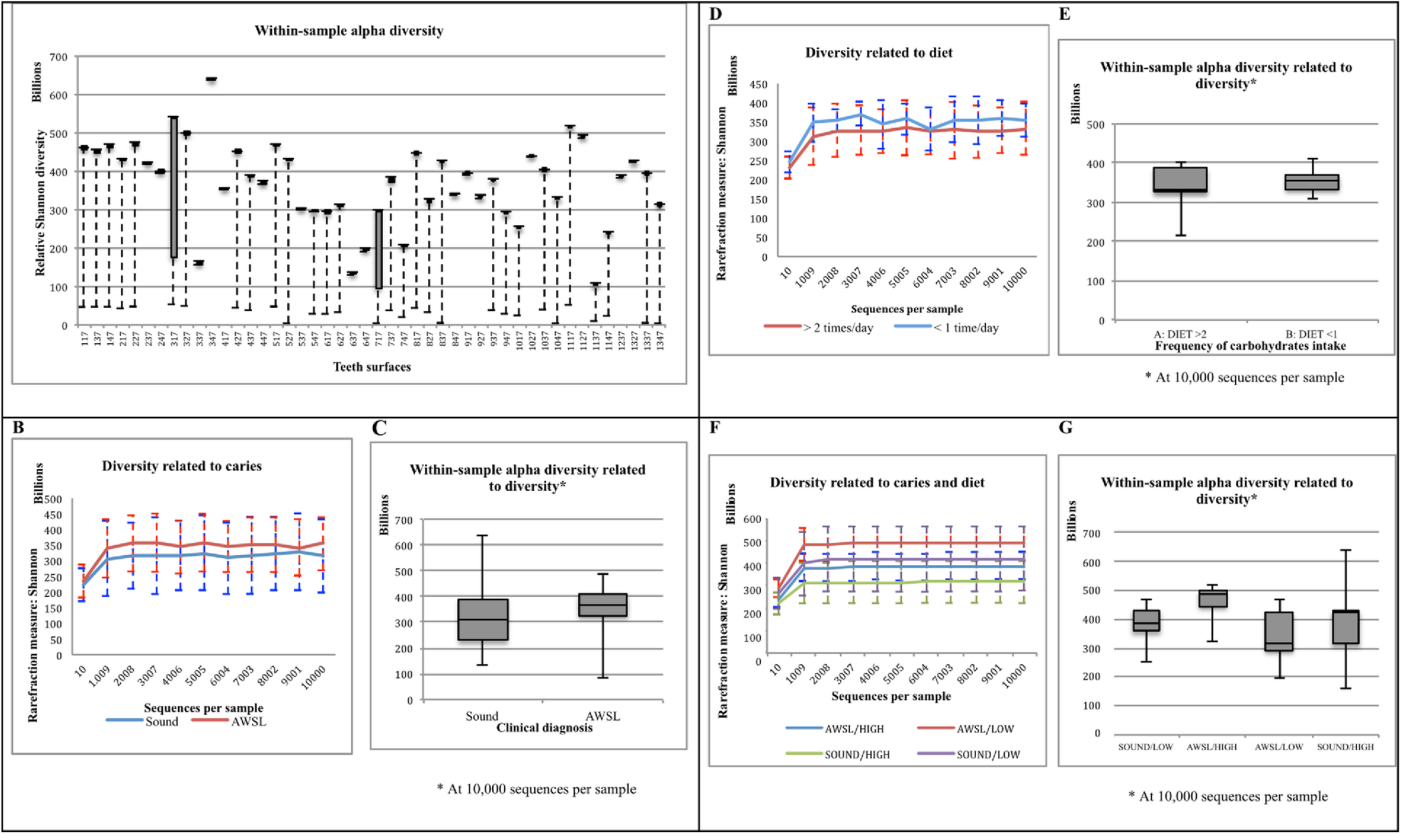
Alpha diversity (Shannon index) of the sequence reads from occlusal biofilm samples. (A) Total samples analyzed. (B and C) Considering the presence of active white spot lesion (AWSL) as a threshold. (D and E) Considering frequency of carbohydrates consumption between meals, as a threshold. (F and G) A comparison of alpha diversity (Shannon index) in relation to surface’s caries diagnosis (AWSL X SOUND) and frequency of fermentable carbohydrates between meals (HIGH X LOW).

Rarefaction analyses (Fig 2) revealed the highest richness in samples 347, 317 and 327 (patient 3; teeth 47, 17 and 27, respectively; Fig 2A). The samples with the lowest richness were 637 and 647 (patient 6) and 947 (patient 9). The Shannon diversity index calculated at 3% dissimilarity (Fig 2A) showed the lowest values of evenness (0.99 and 1.28) for the samples 1137 (patient 11) and 637 (patient 6). Only sample 347 (patient 3) showed an evenness value >6.0.

Comparison of samples based on presence or absence of AWSL suggested a lower Shannon diversity index for sound surfaces (2.16; Fig 2B); however, the difference was not statistically significant. Likewise, the comparison of alpha diversity at the species level according to the frequency of carbohydrates consumption between meals suggested differences in species diversity and richness between the two groups. Low frequency of carbohydrates consumption (≤ 1 time per day, between meals) trended toward higher values of diversity than high frequency of carbohydrates consumption (≥ 2 times per day, between meals) (Fig 2D and 2E), although again the differences were not statistically significant.

**Fig 3:**
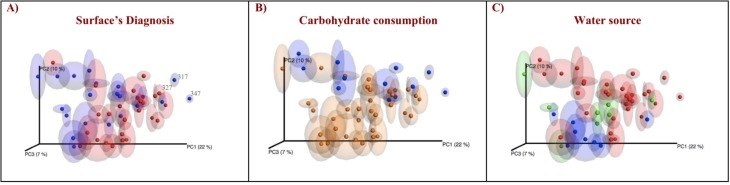
PCoA beta diversity analysis of samples according to surface’s diagnosis, carbohydrate consumption and fluoride source. (A) surface’s diagnosis: sound surfaces are represented in blue and surfaces with AWSL are represented in red; (B) carbohydrate consumption: low frequency are represented in blue and high frequency are represented in yellow. (C) fluoride source by water type: natural (ground) in blue, tap in red (no fluoride on both) and bottled in green.

Principal Coordinate Analysis (PCoA) of unweighted UniFrac showed samples from AWSL did not form a defined cluster away from sound surfaces (Fig 3A). Interestingly, the outliers among “diseased cluster” correspond to sound samples from the same patient and are closely related to the diseased surface. Similarly, we did not observe a clear separation between samples based on carbohydrate consumption (Fig 3B).

The dominant representations for each taxonomic level can be observed in S5 Table. The most abundant taxa at the genus level in both sound surfaces and AWSL were *Streptococcus*, *Pseudomonas*, *Granulicatella*, *Actinomyces*, *Prevotella* and *Veillonella*. *Klebsiella* was over-represented in sound surfaces. Nevertheless, the highest difference in relative abundance (at least 5 times different) when comparing AWSL versus sound surfaces was observed between *Actinobaculum* and *Porphyromonas* in AWSL and *Acinetobacter* in sound surfaces.

**Fig 4:**
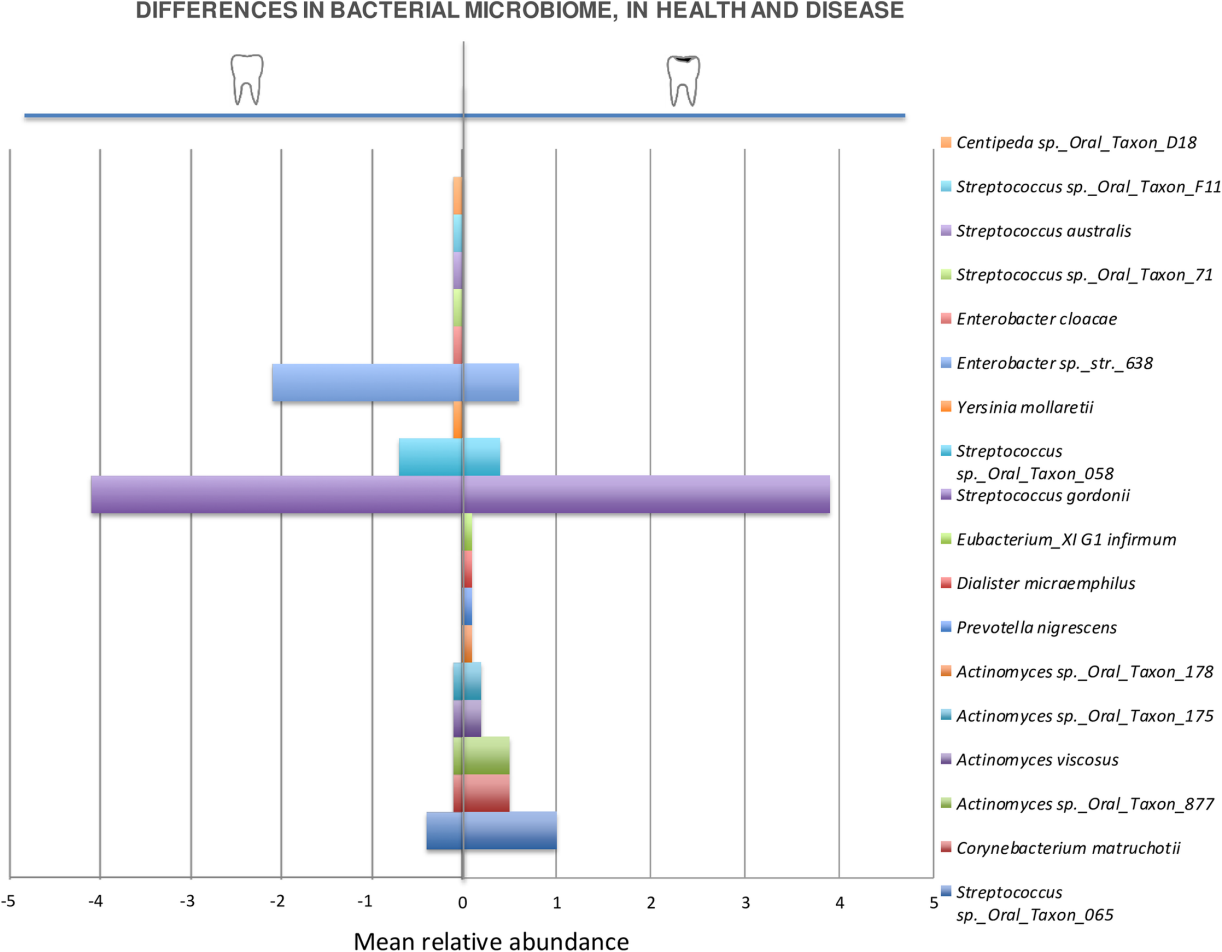
Distribution of bacterial species: Comparison between the sound surfaces (in the left) and the surfaces with caries (AWSL, in the right). *(*p*-values ≤0.05; Mixed effects regression model).

The analysis of a possible association between relative abundances of bacterial species and the surface’s clinical status (AWSL versus Sound) showed statistically significant differences in the relative abundances of 18 species (Table 1) according to clinical status. *Streptococcus sp_Oral_Taxon_065*, *Corynebacterium matruchotii*, *Actinomyces viscosus*, *Actinomyces sp_Oral_Taxon_175*, *Actinomyces sp_Oral_Taxon_178*, *Actinomyces sp_Oral_Taxon_877*, *Prevotella nigrescens*, *Dialister micraerophilus*, *Eubacterium_XI G 1 infirmum* were more abundant on surfaces with AWSL. Conversely, the abundance of *Streptococcus gordonii*, *Streptococcus sp*.*_Oral_Taxon_058*, *Enterobacter sp*.*_str*.*_638*, *Streptococcus australis*, *Yersinia mollaretii*, *Enterobacter cloacae*, *Streptococcus sp*.*_Oral_Taxon_71*, *Streptococcus sp*.*_Oral_Taxon_F11*, *Centipeda sp*.*_Oral_Taxon_D18* was higher on sound surfaces. The relative abundances of bacterial taxa from each surface clinical diagnosis can be observed in Fig 4. Both healthy and diseased sites showed high relative abundances of *Streptococcus mutans*, accounting for an average 7.2% of species abundance. In contrast, *Streptococcus sobrinus* was identified only in three patients (mean relative abundance of 0.6%). No statistically significant correlations were observed between abundances of these two species and caries activity (p = 0.53 and 0.66; respectively).

**Table 1:** Mean relative abundance (SD) comparison with surface diagnosis, at species level, between all patients. Representation of the total species. Only statistically significant P-values are shown (95% confidence interval)^a^.

Specie	Mean (SD) relative abundance—Sound		Mean (SD) relative abundance—AWSL		p-value
*Streptococcus gordonii*	4.1	(6.2)	3.9	(3.3)	0.01
*Streptococcus sp*.*_Oral_Taxon_058*	0.7	(1.3)	0.4	(0.6)	0.05
*Enterobacter sp*.*_str*.*_638*	2.1	(4.3)	0.6	(1.9)	0.05
*Streptococcus sp_Oral_Taxon_065*	0.4	(0.6)	1.0	(1.4)	0.02
*Corynebacterium matruchotii*	0.1	(0.2)	0.5	(0.6)	0.02
*Actinomyces viscosus*	0.1	(0.2)	0.5	(1.3)	0.00
*Actinomyces sp_Oral_Taxon_175*	0.1	(0.2)	0.2	(0.3)	0.00
*Actinomyces sp_Oral_Taxon_178*	0.0	(0.0)	0.0	(0.0)	0.02
*Streptococcus australis*	0.1	(0.3)	0.0	(0.0)	0.04
*Actinomyces sp_Oral_Taxon_877*	0.0	(0.0)	0.0	(0.0)	0.02
*Yersinia mollaretii*	0.1	(0.2)	0.0	(0.0)	0.01
*Enterobacter cloacae*	0.1	(0.2)	0.0	(0.0)	0.03
*Streptococcus sp*.*_Oral_Taxon_71*	0.1	(0.1)	0.0	(0.0)	0.03
*Streptococcus sp*.*_Oral_Taxon_F11*	0.1	(0.1)	0.0	(0.0)	0.00
*Prevotella nigrescens*	0.0	(0.0)	0.1	(0.1)	0.03
*Centipeda sp*.*_Oral_Taxon_D18*	0.1	(0.1)	0.0	(0.0)	0.04
*Dialister micraerophilus*	0.0	(0.0)	0.1	(0.1)	0.01
*Eubacterium_XI G 1 infirmum*	0.0	(0.0)	0.1	(0.1)	0.04

^a^ Mixed effects regression model, p≤0.05

The association between relative abundances of bacterial species and frequency of carbohydrate consumption (high vs low consumption) showed statistically significant differences in the relative abundances of 18 species (complete list on Table 2). Patients whose sugars and starch consumption was less than two times per day between meals (low frequency of carbohydrates consumption), showed statistically significant differences (p≤0.05) in the increased relative abundances of 16 species. Among patients with high frequency of carbohydrates consumption (more than two times between meals), statistically significant differences (p≤0.05) in the increased relative abundances where observed among *Yersinia mollaretti and Streptococcus sp*.*_Oral_Taxon_487*.

**Table 2:** Mean relative abundance (SD) comparison with diet, at species level, between all patients. Representation of the total species. Only statistically significant P-values are shown (95% confidence interval)^a^.

Specie	Mean (SD) relative abundance–Low^b^		Mean (SD) relative abundance–High^b^		p-value
*Porphyromonas sp*.*_Oral_Taxon_279*	0.8	(1.5)	0.1	(0.5)	0.01
*Gemella morbillorum*	0.7	(1.3)	0.0	(0.1)	0.04
*Yersinia mollaretti*	0.0	(0.0)	0.1	(0.2)	0.05
*Fusobacterium nucleatum_ss_polymorphum*	0.3	(0.5)	0.1	(0.2)	0.03
*Porphyromonas catoniae*	0.4	(0.5)	0.0	(0.0)	0.00
*Fusobacterium periodonticum*	0.1	(0.1)	0.0	(0.0)	0.03
*Conynebacterium durum*	0.1	(0.1)	0.0	(0.0)	0.00
*Aggregatibacter sp*.*_Oral_Taxon_458*	0.2	(0.3)	0.0	(0.1)	0.00
*Rothia mucilaginosa*	0.1	(0.2)	0.0	(0.1)	0.00
*Bergeyella sp*.*_Oral_Taxon_322*	0.1	(0.1)	0.0	(0.0)	0.03
*TM7_[G-1] sp*.*_Oral_Taxon_352*	0.0	(0.1)	0.0	(0.0)	0.02
*Alloprevotella sp*.*_Oral_Taxon_308*	0.0	(0.1)	0.0	(0.0)	0.05
*Streptococcus sp*.*_Oral_Taxon_487*	0.0	(0.0)	0.1	(0.1)	0.02
*Agregatibacter segnis*	0.0	(0.1)	0.0	(0.0)	0.04
*Haemophilus haemolyticus*	0.0	(0.1)	0.0	(0.0)	0.05
*Haemophilus sp*.*_Oral Taxon_035*	0.0	(0.1)	0.0	(0.0)	0.04
*Porphyromonas sp_Oral_Taxon_C34*	0.0	(0.1)	0.0	(0.0)	0.02
*Aggregatibacter sp*.*_Oral_Taxon_898*	0.0	(0.1)	0.0	(0.0)	0.04

^a^ Mixed effects regression model, p≤0.05

^b^ Dietary groups–Fermentable carbohydrates frequency consumption between meals (breakfast, lunch and dinner): Low–consumption ≤ 1 time; High–consumption ≥ 2 times

## Discussion

Dental caries continues to be a major public health problem worldwide. It has been shown that the human oral microbiota plays an important role in the health status of the host as the oral cavity contains hundreds of different bacterial species [33]. Cultivation-independent molecular methods, primarily using 16S rRNA gene-based cloning studies, identified approximately 700 species or phylotypes [34,35]. In the present study, by combining 16S rRNA amplicon sequencing and a BLASTN-based search algorithm, we were able to identify collectively 723 species and demonstrated a high bacterial diversity in biofilms collected from the occlusal surface. Considering all teeth, 25 species showed relative abundances higher than 1%. The threshold of relative abundance ≧ 0.1 was chosen because it represented more than 98% of the total sample by each patient, and the results were not influenced if less than 2% of the remaining species were considered in the analyses.

Although previous studies have focused on the oral microbiota of children with and without dental caries, our research is the first to combine NGS and the BLASTN-based search algorithm to determine the bacterial composition in both sound and active white spot lesions on occlusal surfaces at the species level, and to investigate its relationship to dietary factors such as frequency and composition of fermentable carbohydrates. Most of the read classifiers (e.g., the RDP 16S rDNA read classifier), typically use a standard analysis that involves clustering of reads into operational taxonomic units (OTUs), using a Bayesian classifier or BLAST to assign taxonomies to representative OTU sequences. Read alignments are not usually done to calculate the overall percent identity to the reference sequences, and are not performed because BLASTN approach is slow and the procedure would be computationally intensive, requiring significant time for NGS analysis. Hence, these classifiers use the oligonucleotide matching approach (8 mer in the case of RDP classifier) and “guess” the most likely matches using statistical methods. Our BLASTN approach was unique since it aligned each sequence read to a well-curated HOMD reference database and classified only those hits with > = 98% sequence identity with the best hits [19]. Hence, the method is very precise and accurate to achieve identification at species level. Other features of this approach are as follows: higher taxa, such as phylum and genus, levels determination, does not require LCA (lowest common ancestor) assessment, without the capability to accurately classify individual reads to the species level, which is likely more relevant to address, when investigating the link between bacteria at species level and the disease.

The studied population exhibited a high caries experience, confirmed by the indices DMFT-m/DMFS-m and SiC, which were proposed in year 2000 to define individuals with the highest caries scores in different populations [2,36]. The study proposed the oral health goal of a global SiC Index of less than 3 by 2015 among 12-year-olds. Nevertheless, dental caries remains a significant health issue among children worldwide, not restricted only to low income areas.

An important contributing factor for the high caries prevalence in our studied population is the high consumption of sugars and starch separated from main eating events such as lunch and dinner, contributing to acceleration of biofilm formation/maturation and acid production (as classically shown by Loesche [37]), and a decrease in bacterial microbiome diversity. A decreased diversity is due to selection events caused by the acidic environment, as a result of bacterial fermentation. Previous studies have demonstrated the impact of dietary habits on gut microbiome in infants [28] and adults [38]. The present study identified 34 species with low fermentable carbohydrate consumption and 10 other species with high fermentable carbohydrate consumption. Additionally, although not statistically significant, some of the acidogenic species typically involved in the decrease of pH biofilm were highly abundant in biofilm from patients with high carbohydrate consumption, such as *S*. *mutans*, *S*. *mitis*, *Lactobacillus johnsonii*, *Prevotella* spp., *Propionibacterium* spp. and *Actinomyces* spp. Culture techniques have been used extensively to characterize the influence of dietary sugars on aciduric and acidogenic bacteria behavior [39–43]. While these studies identified several bacterial taxa associated with acid production, they were mostly performed by using single species or defined mixed culture models. Our study, however, to the best of our knowledge, is the first to combine NGS with species identification to evaluate the influence of fermentable carbohydrates intake on the abundance of oral bacterial species. From this knowledge, future research will to evaluate the influence of dietary changes in the identified components of the bacterial microbiome.

Among the highly abundant species observed in biofilm from patients with high consumption on carbohydrates, mutans and non-mutans streptococci of several types, including the sanguinis and *S*. *salivarius*, are known to be extremely abundant in the mouth and to have acidogenic and acid tolerant properties [44,45]. However, in terms of its relation to caries development, some data suggest an inverse relationship of the abundance of *S*. *sanguinis* and the mutans streptococci [46]. The interaction between mutans streptococci and *Actinomyces sp*. (which are also carbohydrate users, but are neither acidogenic nor acid tolerant) has been also shown [47]. On the other hand, lactobacilli are characteristically highly acidogenic and extremely acid tolerant. Some lactobacilli are cariogenic in experimental animals and their cariogenicity is dependent upon consumption of carbohydrate rich diets [48]. *Lactobacillus* spp. showed higher counts in dental biofilms *in situ*, in the presence of glucose + fructose and sucrose, [49], and correlations were also found between intake of confectionery-eating events and lactobacillus levels among 12-year-old schoolchildren [50]. Moreover, it was shown that pits and fissures or partially erupted third molars provide a retentive environment favorable to the growth of lactobacilli [51,52].

Our objective was to compare samples from healthy and caries active sites without pooling samples by individualized sample collection to avoid discrepancies in the diversity of bacterial content according to biofilm amount. By comparing bacterial abundances, we demonstrated that sites varied not only between individuals, but also between caries involved samples from the same individual. Members of the genera *Streptococcus*, *Pseudomonas*, *Granulicatella*, *Actinomyces*, *Prevotella and Veillonella* were at the same levels on both sound surfaces and AWSL surfaces, whereas *Actinobaculum* and *Porphyromonas* were at higher percentage in AWSL and *Klebsiella and Acinetobacter* were at higher percentage in sound surface. In contrast, Simón-Soro *et al*. [16] using RNA-seq methods found that *Streptococcus*, *Rothia*, *Leptotrichia* and *Veillonella* were the dominant genera observed among AWSL. Nevertheless, it is important to address that depending on the geographic region and culture, different populations can exhibit differences in microbiome composition. [27]

A recent microbiome study performed on saliva samples from adults with dental caries reported that two bacterial taxa (*Streptococcus salivarius* and *Solobacterium moorei*) and three bacterial clusters (*Streptococcus parasanguinis I* and *II* and sp. clone BE024_ot057/411/721, *Streptococcus parasanguinis I* and *II* and sinensis_ot411/721/767, *S*. *salivarius* and sp. clone FO042_ot067/755) were found at higher levels in caries [53]. We identified 723 taxa and indicated the presence of nine bacterial taxa on carious sites (*Streptococcus sp_Oral_Taxon_065*, *Corynebacterium matruchotii*, *Actinomyces viscosus*, *Actinomyces sp_Oral_Taxon_175*, *Actinomyces sp_Oral_Taxon_178*, *Actinomyces sp_Oral_Taxon_877*, *Prevotella nigrescens*, *Dialister micraerophilus*, *Eubacterium_XI G 1 infirmum*). Nine bacterial taxa (*Streptococcus gordonii*, *Streptococcus sp*.*_Oral_Taxon_058*, *Enterobacter sp*.*_str*.*_638*, *Streptococcus australis*, *Yersinia mollaretii*, *Enterobacter cloacae*, *Streptococcus sp*.*_Oral_Taxon_71*, *Streptococcus sp*.*_Oral_Taxon_F11*, *Centipeda sp*.*_Oral_Taxon_D18*) were present at significantly higher proportions in the sound group. Given our unique approach, it is difficult to compare our results to previous studies, which relied on different technologies and pooled samples.

Both healthy and diseased sites showed high relative abundances of *Streptococcus mutans* and low abundance of *Streptococcus sobrinus*. No relation could be observed between these species and the presence of AWSL. These two species have been investigated for many years and are traditionally recognized as the most cariogenic species [37,54,55]. The role of these species as a primary caries pathogen has also been reinforced among populations without routine caries treatment and prevention strategies [56], similar to the population in our study. Nevertheless, our study corroborates findings from Aas *et al*. [12] and Simón-Soro *et al*. [16] showing that bacterial species other than *S*. *mutans* and *S*. *sobrinus*, e.g., species of the genera *Lactobacillus*, *Prevotella*, *Propionibacterium*, non-*S*. *mutans* streptococci and *Actinomyces* spp., may also play important roles in caries initiation and biofilm community interactions.

Our results involving surface diagnosis, diet and bacterial diversity supported the ecological plaque hypothesis and the influence of dietary habits on bacterial diversity [47,57]. We showed that when only surface clinical diagnosis was considered, a weak relation was observed with bacterial diversity. On the other hand, a higher and significant relation was verified when fermentable carbohydrate intake was correlated with lower bacterial diversity. Thus, it reinforces that dental biofilm is a dynamic and stable microbial ecosystem and dental caries is a biofilm-sucrose-dependent disease. Where, due to microbial metabolism of fermentable carbohydrates, the pH decreases in the biofilm leading to a change in the environment, with microbial acid-induced adaptation and subsequent selection of ‘low-pH’ bacteria. In turn, these ‘low-pH’ bacteria play a critical role in biofilm dysbiosis by facilitating a shift from the oral bacterial microbiome associated with health and demineralization/remineralization balance and ultimately to a consistent mineral loss (i.e., demineralization driven by an acidogenic state). This change in the ecological environment may enhance acidogenicity and acidurance of the non-mutans bacteria adaptively and hence enrich for those species able to survive in the acidic environment.

It should be noted that given the large number of bacteria examined and the sample size, it is likely that some of the bacteria identified in this study are false positives. Thus, the list of bacteria associated with AWSL or carbohydrate consumption should be interpreted cautiously. These lists should not be regarded as definitive but rather as preliminary findings that need to be confirmed in future studies.

In conclusion, our study showed that high consumption of fermentable carbohydrates was associated with a reduction in bacterial diversity. We also observed great variability in the diversity of biofilm microbiome among sound and caries active sites. These data provide a deeper understanding of the differences in bacterial composition associated with health and initial caries development in enamel. By using high-throughput sequencing and BLASTN, we were able to determine bacterial associations mostly at the species level. Consequently, we were able to generate new data of the bacterial community profiles of the occlusal surface, possibly related to a shift between health and disease. Finally, our study suggests that species other than mutans streptococci may also be associated with the initiation of dental caries on occlusal surfaces.

## Supporting information

S1 Fig Semi-quantitative food-frequency questionnaire (QFASQ) form. (PDF)

S2 Fig Relative abundance of (A) phyla, (B) classes, (C) orders, (D) families, (E) genera and (F) species. Left plots show sound surfaces, right plots show surfaces with active white spot lesions (AWSL).
(PDF)

S1 Table Sample distribution according to gender, teeth, oral health status, dietary habits and fluoride source. (PDF)

S2 Table Sample distribution according to gender, teeth, clinical diagnosis and number of sequences obtained. (PDF)

S3 Table Mean relative abundances (RA) of all species represented over 0.1%, by patient (Pat). (PDF)

S4 Table Mean relative abundances (RA) of all species represented over 0.1%, by individual biofilm sample. (PDF)

S5 Table Microbiome diversity measures in each taxonomic level, by clinical diagnosis categories. (PDF)
