# Supplementary material for: The oral bacterial microbiome of occlusal surfaces in children and its association with diet and caries
Source: PLoS One. 2017 Jul 5;12(7):e0180621. doi: 10.1371/journal.pone.0180621 (PMC5498058; doi:10.1371/journal.pone.0180621)
Supplement: S1 Fig — (PDF) [file pone.0180621.s001.pdf]

The Oral Bacterial Microbiome of Occlusal Surfaces in Children and its Association with Diet And Caries

S1 Fig. Semi-quantitative food-frequency questionnaire (QFASQ) form.

UNIVERSIDADE FEDERAL FLUMINENSE

PÓLO UNIVERSITÁRIO DE NOVA FRIBURGO

Faculdade de Odontologia – FOUFF/NF

Patient number: ____________

SEMI-QUANTITATIVE FOOD-FREQUENCY QUESTIONNAIRE (QFASQ)

How many times did you eat these foods, during the last 6 months?

FOODS	PORTION	INTAKE						
		Never	Less than once a month	1 to 3 times per month	Once a week	2 to 4 times weekly	Once daily	Twice or more times daily
**Rice, Bread, Pasta, Potato**								
Rice	3 soup spoons							
Mashed potato	1 soup spoon							
French fries	1 portion							
Bread	1 unit							
Pasta	1 portion							
Pizza	1 slice							
Lamen	1 portion							
“Pão de queijo”	3 units							
**Grains**								
Beans	3 soup spoons							
Soy	1 soup spoon							
Oatmeal	1 soup spoon							
Breakfast cereals	1 soup spoon							
**Sweets, salty snacks, goodies**								
Potato chips	1 bag							
Popcorn	1 bag							
Salty snacks	1 bag							
Chocolate	3 units							
Cookies	4 units							
Candy	1 unit							
Cake	1 slice							
Sugar	1 tea spoon							
Chocolate flavored powder	1 ½ spoon							
Ice cream	2 scoops/1 unit							
Hot dog	1 unit							
Burger	1 unit							
Dessert	1 portion							

**Drinks**								
Soft drink	1 cup							
Water	1 cup							
Natural juice	1 cup							
Industrialized juice	1 cup							
“Guaraná”	1 cup							
Ice Tea	1 cup							
Gatorade	1 cup							
Soy flavored drink	1 cup							
Milk	1 cup							
Milk with chocolate powder	1 cup							
Coffee (with sugar)	1 cup							
**Fruit**								
Apple	1 unit							
Orange	1 unit							
Melon	1 slice							
Pineapple	1 slice							
Banana	1 unit							
Pear	1 unit							
**Meat/eggs**								
Red meat	1 portion							
Ham	3 slices							
Fish	1 portion							
Chicken	1 portion							
Pork	1 portion							
**Vegetable**								
Carrot	1 portion							
“Chuchu”	1 portion							
Manioc	1 portion							
Green leafs	1 portion							
Tomato	1 portion							
Pumpkin	1 portion							
**Milk derivatives**								
Cheese	2 slices							
Yogurt	1 unit							
Pudding	1 unit							
Butter/ margarine	1 ½ tea spoon							
“Requeijão”	1 ½ tea spoon							
