# Supplementary material for: The oral bacterial microbiome of occlusal surfaces in children and its association with diet and caries
Source: PLoS One. 2017 Jul 5;12(7):e0180621. doi: 10.1371/journal.pone.0180621 (PMC5498058; doi:10.1371/journal.pone.0180621)
Supplement: S2 Fig — Relative abundance of (A) phyla, (B) classes, (C) orders, (D) families, (E) genera and (F) species. Left plots show sound surfaces, right plots show surfaces with active white spot lesions (AWSL). (PDF) [file pone.0180621.s002.pdf]

The Oral Bacterial Microbiome of Occlusal Surfaces in Children and its Association with Diet And Caries

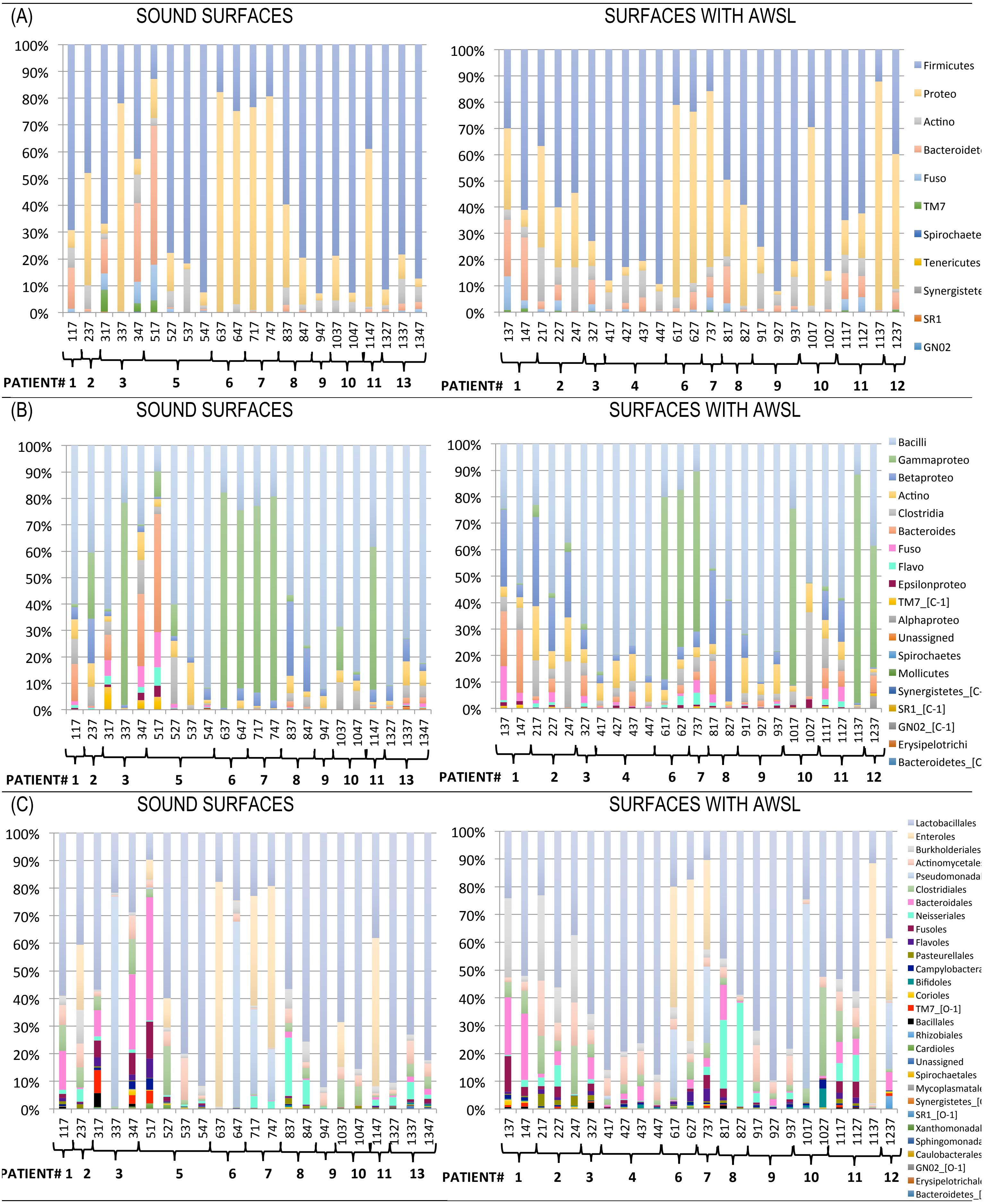

S2 Fig. Relative abundance of (A) phyla, (B) classes, (C) orders, (D) families, (E) genera and (F) species. Left plots show sound surfaces, right plots show surfaces with active white spot lesions (AWSL).

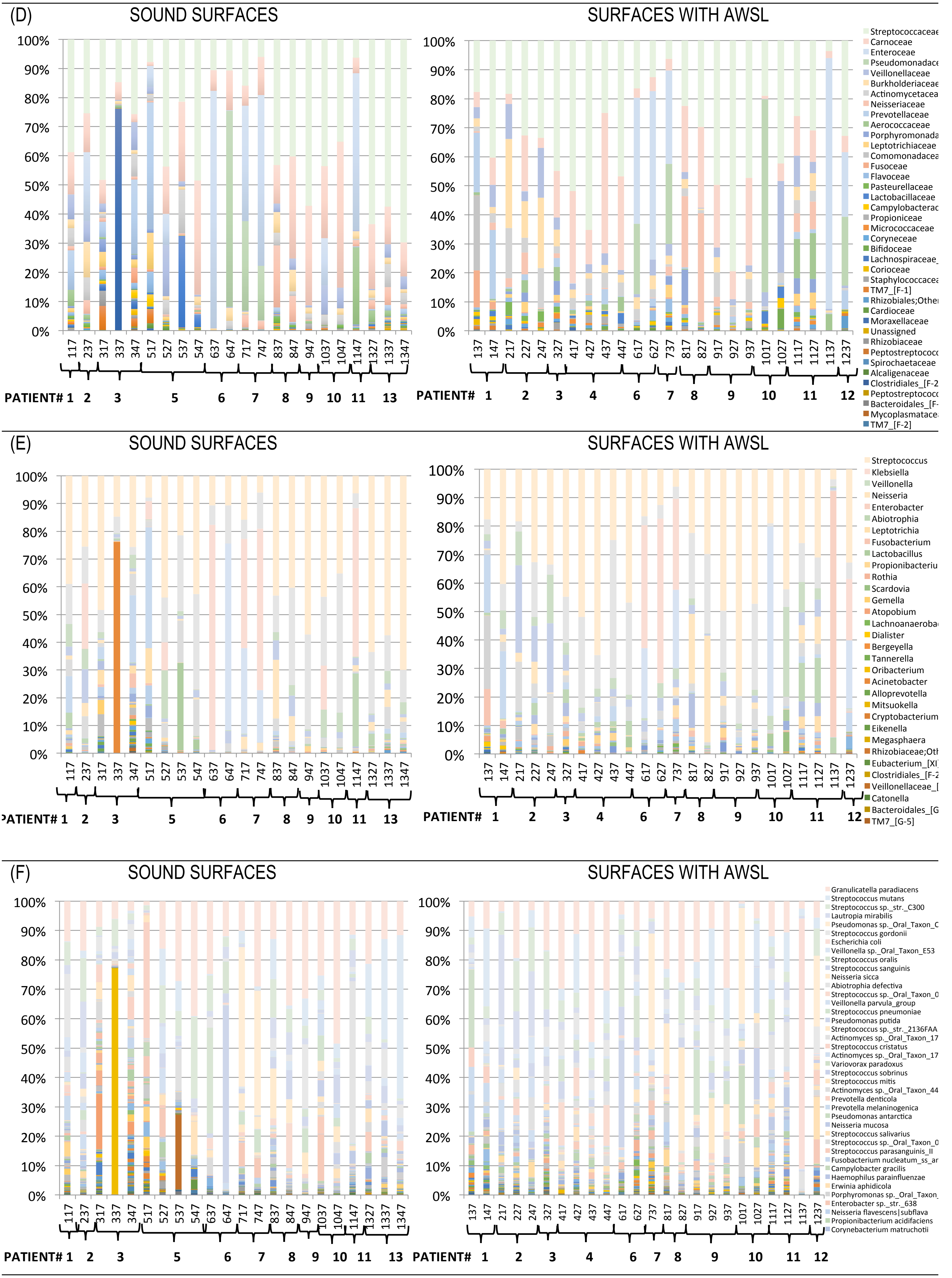
(D)
SOUND SURFACES
SURFACES WITH AWSL
PATIENT# 1 2 3 5 6 7 8 9 10 11 13
PATIENT# 1 2 3 4 6 7 8 9 10 11 12
Streptococcaceae
Carnoceae
Enteroceae
Pseudomonadace
Veillonellaceae
Burkholderiaceae
Actinomycetacea
Neisseriaceae
Prevotellaceae
Aerococcaceae
Porphyromonada
Leptotrichiaceae
Comomonadacea
Fusoceae
Flavoceae
Pasteurellaceae
Lactobacillaceae
Campylobacterac
Propioniceae
Micrococcaceae
Coryneceae
Bifidoceae
Lachnospiraceae_
Corioceae
Staphylococcacea
TM7_[F-1]
Rhizobiales;Other
Cardioceae
Moraxellaceae
Unassigned
Rhizobiaceae
Peptostreptococc
Spirochaetaceae
Alcaligenaceae
Clostridiales_[F-2
Peptostreptococc
Bacteroidales_[F-
Mycoplasmatacea
TM7_[F-2]
(E)
SOUND SURFACES
SURFACES WITH AWSL
PATIENT# 1 2 3 5 6 7 8 9 10 11 13
PATIENT# 1 2 3 4 6 7 8 9 10 11 12
Streptococcus
Klebsiella
Veillonella
Neisseria
Enterobacter
Abiotrophia
Leptotrichia
Fusobacterium
Lactobacillus
Propionibacteriu
Rothia
Scardovia
Gemella
Atopobium
Lachnoanaeroba
Dialister
Bergeyella
Tannerella
Oribacterium
Acinetobacter
Alloprevotella
Mitsuokella
Cryptobacterium
Eikenella
Megasphaera
Rhizobiaceae;Oth
Eubacterium_[XI
Clostridiales_[F-2
Veillonellaceae_[
Catonella
Bacteroidales_[G
TM7_[G-5]
(F)
SOUND SURFACES
SURFACES WITH AWSL
PATIENT# 1 2 3 5 6 7 8 9 10 11 13
PATIENT# 1 2 3 4 6 7 8 9 10 11 12
Granulicatella paradiacens
Streptococcus mutans
Streptococcus sp._str._C300
Lautropia mirabilis
Pseudomonas sp._Oral_Taxon_C
Streptococcus gordonii
Escherichia coli
Veillonella sp._Oral_Taxon_E53
Streptococcus oralis
Streptococcus sanguinis
Neisseria sicca
Abiotrophia defectiva
Streptococcus sp._Oral_Taxon_0
Veillonella parvula_group
Streptococcus pneumoniae
Pseudomonas putida
Streptococcus sp._str._2136FAA
Actinomyces sp._Oral_Taxon_17
Streptococcus cristatus
Actinomyces sp._Oral_Taxon_17
Variovorax paradoxus
Streptococcus sobrinus
Streptococcus mitis
Actinomyces sp._Oral_Taxon_44
Prevotella denticola
Prevotella melaninogenica
Pseudomonas antarctica
Neisseria mucosa
Streptococcus salivarius
Streptococcus sp._Oral_Taxon_0
Streptococcus parasanguinis_II
Fusobacterium nucleatum_ss_ar
Campylobacter gracilis
Haemophilus parainfluenzae
Erwinia aphidicola
Porphyromonas sp._Oral_Taxon_
Enterobacter sp._str._638
Neisseria flavescens|subflava
Propionibacterium acidifaciens
Corynebacterium matruchotii
