# Supplementary material for: The oral bacterial microbiome of occlusal surfaces in children and its association with diet and caries
Source: PLoS One. 2017 Jul 5;12(7):e0180621. doi: 10.1371/journal.pone.0180621 (PMC5498058; doi:10.1371/journal.pone.0180621)
Supplement: S1 Table — (PDF) [file pone.0180621.s003.pdf]

The Oral Bacterial Microbiome of Occlusal Surfaces in Children and its Association with Diet And Caries

S1 Table: Sample distribution according to gender, teeth, oral health status, dietary habits and fluoride source

	Oral Health status					
		Tooth identification number				
Gender		17	27	37	47	Total
Boys N=6	AWSL*	2	3	2	1	8
	Sound	3	2	4	5	14
Girls N=7	AWSL*	5	5	4	2	16
	Sound	1	0	3	4	8
Total (N=13)		11	10	13	12	46
DMFT**		6.31 ± 4.25				
DMFS**		8.31 ± 7.15				
SiC***		11.75				

	Dietary habits					
Gender		Carbohydrate	Sugar	Soft drink/ artificial juice	Water	Snack
Boys N=6	<3X/month	1	1	1	-	2
	1-4X/week	1	1	1	-	1
	1X/day	-	2	2	2	2
	≥2X/day	4	2	2	4	1
Girls N=7	<3X/month	-	-	1	-	-
	1-4X/week	-	2	3	-	2
	1X/day	2	1	3	1	3
	≥2X/day	5	4	-	6	2

	Fluoride/water consumed source			
Gender	Toothpaste	Tap water$	Natural source	Water bottle
Boys N=6	5	5	1	-
Girls N=7	7	4	1	2

* Active white spot lesions were considered caries. Total based only occlusal surface.

** DMFT for Decayed, Missing, Filled Teeth; DMFS for Decayed, Missing, Filled Surface. Mean based on all erupted teeth.

*** Significant Caries Index.

$ No fluoride content.

Order, Family, Genus and Specie, respectively.
