# Supplementary material for: The oral bacterial microbiome of occlusal surfaces in children and its association with diet and caries
Source: PLoS One. 2017 Jul 5;12(7):e0180621. doi: 10.1371/journal.pone.0180621 (PMC5498058; doi:10.1371/journal.pone.0180621)
Supplement: S2 Table — (PDF) [file pone.0180621.s004.pdf]

The Oral Bacterial Microbiome of Occlusal Surfaces in Children and its Association with Diet And Caries

S2 Table: Sample distribution according to gender, teeth, clinical diagnosis and number of sequences obtained.

Patient #	Gender A: fem; B: masc	# of teeth	DMFT*	DMFS*	Teeth#	Sample ID	Eruption stage¶	Occlusal surface diagnosis	Total reads number	Total reads matched unique species	Total reads matched multiple species of the same Genus	Match percent age a species level
1	A	23	9	12	17	117	Partial	Sound	200.158	184.932	1.218	92.39%
					37	137	Total	AWSL	306.685	292.734	984	95.45%
					47	147	Total	AWSL	303.798	262.690	7.093	86.47%
2	A	28	5	5	17	217	Total	AWSL	288.487	268.134	6.082	92.94%
					27	227	Total	AWSL	253.134	239.436	2.747	94.59%
					37	237	Total	Sound	304.090	237.367	2.837	78.06%
					47	247	Total	AWSL	246.195	234.643	1.231	95.31%
3	B	22	5	8	17	317	Total	Sound	98.984	88.308	956	89.21%
					27	327	Total	AWSL	135.180	124.760	3.199	92.29%
					37	337	Total	Sound	182.961	177.834	914	97.20%
					47	347	Total	Sound	237.947	214.599	8.658	90.19%
4	B	28	16	29	17	417	Total	AWSL	290.478	270.565	7.726	93.14%
					27	427	Total	AWSL	167.783	142.419	1.593	84.88%
					37	437	Total	AWSL	347.104	330.460	3.799	95.20%
					47	447	Total	AWSL	299.994	274.436	11.805	91.48%
5	B	28	0	0	17	517	Total	Sound	263.013	161.779	2.631	61.51%
					27	527	Partial	Sound	195.197	102.566	1.051	52.54%
					37	537	Partial	Sound	95.578	83.474	5.325	87.34%
					47	547	Total	Sound	151.546	112.782	722	74.42%
6	B	28	2	2	17	617	Total	AWSL	195.146	114.230	300	58.54%
					27	627	Total	AWSL	150.884	65.620	357	43.49%
					37	637	Total	Sound	99.630	24.805	86	24.90%
					47	647	Total	Sound	145.338	141.057	296	97.05%
7	B	27	5	5	17	717	Partial	Sound	225.488	121.704	797	53.97%
					37	737	Total	AWSL	160.199	98.818	971	61.68%
					47	747	Total	Sound	126.714	50.033	375	39.48%
8	A	28	4	4	17	817	Total	AWSL	172.883	164.221	976	94.99%
					27	827	Partial	AWSL	153.646	147.069	952	95.72%
					37	837	Total	Sound	219.075	192.388	895	87.82%
					47	847	Total	Sound	172.623	162.681	3.586	94.24%
9	A	28	13	15	17	917	Total	AWSL	266.986	250.344	1.446	93.77%
					27	927	Partial	AWSL	123.175	115.451	345	93.73%
					37	937	Partial	AWSL	119.780	113.793	553	95.00%
					47	947	Total	Sound	132.232	124.851	573	94.42%
10	A	28	9	9	17	1017	Total	AWSL	362.829	346.161	5.875	95.41%
					27	1027	Total	AWSL	83.797	76.571	2.813	91.38%
					37	1037	Total	Sound	302.614	181.080	5.568	59.84%

					47	1047	Total	Sound	188.643	178.989	1.730	94.88%
11	A	28	3	3	17	1117	Partial	AWSL	191.233	179.238	1.348	93.73%
					27	1127	Total	AWSL	262.467	245.491	1.475	93.53%
					37	1137	Total	AWSL	281.283	245.257	212	87.19%
					47	1147	Total	Sound	129.023	56.187	155	43.55%
12	A	22	5	8	37	1237	Partial	AWSL	189.871	102.723	784	54.10%
13	B	27	6	8	27	1327	Partial	Sound	320.396	303.310	2.165	94.67%
					37	1337	Total	Sound	212.271	105.376	477	49.64%
					47	1347	Total	Sound	232.880	156.723	682	67.30%

* Indexes included active white spot lesions (AWSL) in caries component.

¶ Eruption stage: Total when tooth had reached the occlusion line in the arch. Partial when tooth was in infra-occlusion.
