# Supplementary material for: The oral bacterial microbiome of occlusal surfaces in children and its association with diet and caries
Source: PLoS One. 2017 Jul 5;12(7):e0180621. doi: 10.1371/journal.pone.0180621 (PMC5498058; doi:10.1371/journal.pone.0180621)
Supplement: S3 Table — (PDF) [file pone.0180621.s005.pdf]

The Oral Bacterial Microbiome of Occlusal Surfaces in Children and its Association with Diet And Caries

S3 Table. Mean relative abundances (RA) of all species represented over 0.1%, by patient (Pat).

Relative abundance ≥ 0.1		Patient # (DMFT; # of AWSL/ # of second molars)													
**Taxon_ID**	**Species**	**Pat 1 (9; 2/3)**	**Pat 2 (5; 3/4)**	**Pat 3 (5; 1/4)**	**Pat 4 (16; 4/4)**	**Pat 5 (0; 0/4)**	**Pat 6 (2; 2/4)**	**Pat 7 (5; 1/3)**	**Pat 8 (4; 2/4)**	**Pat 9 (13; 3/4)**	**Pat 10 (9; 2/4)**	**Pat 11 (3; 3/4)**	**Pat 12 (5; 1/1)**	**Pat 13 (6; 0/3)**	**Mean RA**
GGT673;HOT736	*Granulicatella paradiacens*	8.7	10.2	7.4	33.2	18.3	10.0	11.9	24.1	22.7	17.9	8.2	5.5	16.8	**15.1**
GGT1791;HOT578	*Streptococcus mutans*	12.1	2.3	0.4	8.5	4.7	0.7	2.2	5.5	11.3	2.7	6.3	3.7	31.1	**7.1**
GGT1534;HOT536	*Pseudomonas sp._Oral_Taxon_C61*	0.0	0.0	0.0	0.0	0.0	0.0	43.3	0.0	0.0	17.4	0.0	23.7	0.0	**6.5**
HOTB66	*Streptococcus sp._str._C300*	4.3	13.1	7.8	4.9	11.5	4.9	0.4	9.2	4.9	1.3	2.4	9.1	3.6	**6.0**
GGT1758	*Streptococcus gordonii*	7.9	1.0	3.9	6.1	0.0	1.4	0.2	3.5	10.2	0.1	2.0	9.0	7.2	**4.1**
GGT866;HOT718; HOT826	*Lautropia mirabilis*	2.7	20.2	2.0	2.6	1.3	5.5	2.1	4.8	3.1	0.6	3.7	0.1	2.2	**3.9**
GGT1806;HOT707	*Streptococcus sanguinis*	0.9	4.5	2.2	3.9	1.9	5.5	1.4	5.0	3.0	2.8	1.3	0.2	4.5	**2.9**
GGT1352;HOT544	*Pseudomonas putida*[a]	0.0	0.0	0.0	0.0	0.0	36.6	0.0	0.0	0.0	0.0	0.0	0.0	0.0	**2.8**
HOT660	*Escherichia coli*[a]	0.0	0.0	0.0	0.0	0.0	0.0	0.0	0.0	0.0	0.0	28.7	0.0	0.0	**2.3**
GGT1820	*Veillonella sp._Oral_Taxon_E53*	3.2	5.9	1.9	1.7	2.2	1.1	1.6	1.7	1.6	3.3	2.7	0.3	1.3	**2.2**
GGT1803;HOT622	*Streptococcus oralis*	0.0	0.3	0.1	4.7	0.0	15.2	0.1	0.1	3.0	0.0	2.9	0.0	0.0	**2.1**
HOT279	*Prevotella denticola*	4.8	0.0	2.3	0.4	12.5	0.0	0.1	0.0	0.4	0.0	0.3	5.1	0.0	**2.0**
HOTG60	*Abiotrophia defectiva*	1.7	1.8	1.4	2.5	0.1	0.0	0.1	0.3	0.4	0.1	14.1	0.1	2.7	**2.0**
GGT1304;HOT598	*Neisseria sicca*	0.1	1.8	0.0	0.0	0.0	0.0	4.1	18.8	0.0	0.0	0.0	0.1	0.0	**1.9**
HOT215	*Acinetobacter baumannii*[a]	0.0	0.0	22.6	0.0	0.0	0.0	0.0	0.0	0.0	0.0	0.0	0.0	0.0	**1.8**
GGT397;HOT633	*Enterobacter sp._str._638*	0.0	0.0	0.0	0.0	4.4	0.1	6.7	0.0	0.0	3.7	0.0	8.0	0.0	**1.8**
GGT1787;HOT398; HOT677	*Streptococcus pneumoniae*	0.2	0.9	0.2	0.4	0.0	0.2	3.5	0.4	12.7	3.1	0.1	0.1	0.0	**1.7**
GGT1822	*Veillonella parvula_group*	1.9	3.8	0.9	0.8	2.1	1.3	0.7	0.6	0.5	5.4	1.2	0.1	1.0	**1.6**
HOT768	*Streptococcus sp._Oral_Taxon_064*	0.1	0.1	0.1	10.4	0.1	1.1	0.0	6.0	0.1	0.0	0.0	0.6	0.2	**1.5**
HOT540	*Erwinia aphidicola*[a]	0.0	0.0	0.0	0.0	0.0	0.0	0.0	0.0	0.0	0.0	0.0	14.2	0.0	**1.1**
GGT1676;HOT151	*Streptococcus cristatus*	0.6	1.9	2.6	0.6	0.4	0.3	1.4	0.6	1.3	0.1	2.0	0.3	1.9	**1.1**
GGT1799;HOT686	*Streptococcus salivarius*	0.0	0.2	0.4	2.1	3.5	0.1	0.3	0.1	0.1	2.2	0.2	1.4	2.6	**1.0**
GGT1788;HOT576	*Streptococcus mitis*	0.2	0.3	2.9	0.6	1.2	1.2	0.5	1.1	0.6	0.6	1.4	2.3	0.3	**1.0**

HOT431	*Streptococcus sp._str._2136FAA*	0.3	0.0	0.0	0.0	0.0	0.0	1.3	0.0	7.7	3.2	0.0	0.0	0.0	**1.0**
HOT170	*Actinomyces sp._Oral_Taxon_171*	0.1	4.8	0.1	1.4	2.6	0.1	0.0	0.0	0.1	2.6	0.5	0.1	0.2	**1.0**
HOT058	*Streptococcus sp._Oral_Taxon_071*	0.9	0.2	1.9	0.3	0.3	0.9	0.1	0.0	1.4	1.0	0.4	2.0	2.2	**0.9**
GGT1484;HOT291	*Prevotella melaninogenica*	7.5	0.3	1.1	0.8	0.1	0.2	0.1	0.1	0.1	0.0	0.1	0.1	0.2	**0.8**
GGT1808;HOT644	*Streptococcus parasanguinis_II*	0.0	0.1	0.5	0.8	0.3	0.1	0.2	0.9	0.2	3.2	0.1	0.3	3.9	**0.8**
GGT1536;HOT612	*Variovorax paradoxus*[a]	10.3	0.0	0.0	0.0	0.0	0.0	0.0	0.0	0.0	0.0	0.0	0.0	0.0	**0.8**
HOT169	*Actinomyces sp._Oral_Taxon_170*	0.1	5.3	0.8	0.2	0.1	2.6	0.0	0.0	0.0	0.1	0.6	0.0	0.0	**0.8**
GGT1002;HOT731	*Neisseria mucosa*	0.1	0.2	0.1	0.0	0.0	0.0	0.1	0.1	1.8	0.0	3.8	0.1	2.7	**0.7**
GGT1300;HOT682	*Pseudomonas antarctica*[a]	0.0	0.0	0.0	0.0	0.0	0.0	0.0	0.0	0.0	8.8	0.0	0.0	0.0	**0.7**
GGT1795;HOT755	*Streptococcus sp._Oral_Taxon_058*	0.0	0.5	0.5	0.1	1.6	0.0	1.4	0.2	0.3	0.0	0.8	0.4	2.3	**0.6**
HOT446	*Actinomyces sp._Oral_Taxon_448*	0.1	0.3	0.6	2.5	0.0	0.2	0.3	0.2	1.8	1.1	0.6	0.0	0.0	**0.6**
GGT1811;HOT721	*Streptococcus sobrinus*	0.0	0.6	0.0	0.0	0.0	0.0	0.0	0.0	0.0	6.0	0.0	1.0	0.0	**0.6**
GGT1818;HOT021	*Fusobacterium nucleatum_ss_animalis*	5.8	0.0	1.2	0.0	0.1	0.0	0.0	0.0	0.0	0.0	0.0	0.1	0.0	**0.6**
HOT893	*Actinomyces sp._Oral_Taxon_169*	0.6	0.5	0.0	0.1	0.6	0.1	0.0	0.9	1.0	0.5	0.4	0.1	2.1	**0.5**
HOTA58	*Campylobacter gracilis*	1.2	0.1	0.9	0.3	1.6	0.7	0.3	0.2	0.6	0.5	0.1	0.1	0.0	**0.5**
GGT706;HOT577	*Haemophilus parainfluenzae*	0.5	2.7	0.4	0.0	0.0	0.1	0.2	1.0	0.1	0.4	0.4	0.0	0.2	**0.5**
HOTC21	*Neisseria flavescens|subflava*	0.0	1.1	0.4	0.1	0.0	0.0	1.1	2.5	0.0	0.0	0.2	0.0	0.2	**0.4**
HOT057	*Streptococcus sp._Oral_Taxon_070*	0.4	0.0	4.1	0.1	0.1	0.0	0.0	0.2	0.0	0.0	0.0	0.4	0.3	**0.4**
HOT056	*Streptococcus sp._Oral_Taxon_065*	0.0	0.4	0.4	0.3	0.1	0.1	0.9	0.6	0.0	0.2	0.1	0.0	2.4	**0.4**
GGT1;HOT534	*Lactobacillus delbrueckii*[a]	0.0	0.0	0.0	0.0	0.0	0.0	0.0	0.0	0.0	5.0	0.0	0.0	0.0	**0.4**
GGT1023	*Lactobacillus paracasei*	0.0	0.0	0.0	0.0	4.6	0.0	0.0	0.1	0.0	0.0	0.0	0.0	0.2	**0.4**
HOT283	*Porphyromonas sp._Oral_Taxon_279*	0.1	0.1	0.5	0.0	0.0	0.1	1.2	1.5	0.0	0.0	0.7	0.0	0.1	**0.3**
HOTD12	*Propionibacterium acidifaciens*	0.0	0.1	0.1	1.5	0.0	0.0	0.1	0.1	2.2	0.3	0.0	0.0	0.0	**0.3**
HOT175	*Actinomyces sp._Oral_Taxon_180*	0.7	1.2	1.4	0.0	0.2	0.1	0.0	0.4	0.0	0.0	0.1	0.1	0.0	**0.3**
GGT155;HOT723	*Corynebacterium matruchotii*	0.0	1.0	0.3	0.5	0.2	0.0	0.8	0.3	0.2	0.0	0.7	0.0	0.2	**0.3**
HOTC61	*TM7_[G-1] sp._Oral_Taxon_346*	0.4	0.1	0.8	0.0	1.6	0.0	0.3	0.0	0.0	0.0	0.0	0.9	0.0	**0.3**
HOT284	*Prevotella histicola*	1.8	0.1	0.0	0.3	0.0	0.1	0.0	0.4	0.1	0.1	0.3	0.0	0.8	**0.3**
HOT203	*Leptotrichia sp._AF189244.1*	0.1	0.4	0.2	0.3	0.4	0.1	0.1	0.2	0.1	0.0	1.9	0.0	0.2	**0.3**
GGT1485;HOT311	*Prevotella salivae*	0.8	0.1	0.8	0.3	1.2	0.0	0.0	0.1	0.0	0.0	0.1	0.3	0.0	**0.3**
GGT1498;HOT466	*Capnocytophaga gingivalis*	0.1	0.2	0.2	0.0	1.5	0.4	0.8	0.1	0.0	0.0	0.2	0.0	0.0	**0.3**
HOT183	*Actinobaculum sp._Oral_Taxon_183*	0.0	0.0	0.1	0.9	0.0	0.8	0.1	0.0	1.6	0.0	0.0	0.0	0.0	**0.3**
GGT1078;HOT224	*Leptotrichia sp._Oral_Taxon_417*	0.2	0.4	0.9	0.0	0.2	0.1	1.1	0.1	0.0	0.0	0.5	0.0	0.1	**0.3**
HOT065	*Streptococcus sp._Oral_Taxon_B66*	0.0	0.7	0.0	0.1	1.7	0.0	0.2	0.0	0.0	0.0	0.7	0.0	0.0	**0.3**

HOTE75	*Actinomyces viscosus*	1.4	0.2	0.2	0.4	0.1	0.0	0.1	0.0	0.5	0.0	0.3	0.0	0.2	**0.3**
GGT1075	*Leptotrichia wadei*	0.2	0.4	0.5	0.1	1.6	0.0	0.0	0.0	0.1	0.0	0.3	0.0	0.0	**0.2**
GGT1681;HOT130	*Streptococcus anginosus*	1.9	0.0	0.5	0.0	0.0	0.1	0.0	0.0	0.0	0.0	0.0	0.5	0.0	**0.2**
GGT1625;HOT188	*Scardovia wiggsiae*	0.2	0.1	0.0	0.6	0.1	0.1	0.0	0.2	0.2	1.0	0.0	0.2	0.3	**0.2**
HOTE63	*Actinomyces sp._Oral_Taxon_E75*	0.3	0.0	0.0	0.0	0.0	0.1	0.0	0.7	0.0	0.0	0.0	1.8	0.0	**0.2**
GGT53;HOT701	*Actinomyces oris*	0.0	2.3	0.0	0.1	0.0	0.0	0.0	0.0	0.0	0.0	0.2	0.0	0.0	**0.2**
GGT1077;HOT214	*Leptotrichia sp._Oral_Taxon_498*	0.0	0.0	0.2	0.0	2.4	0.0	0.0	0.0	0.0	0.0	0.0	0.0	0.0	**0.2**
HOT191	*Rothia dentocariosa*	0.0	0.2	0.3	0.6	0.0	0.0	0.0	0.1	0.5	0.3	0.3	0.0	0.2	**0.2**
GGT999;HOT706	*Neisseria flava*	1.2	0.1	0.0	0.1	0.0	0.0	0.0	1.1	0.0	0.0	0.0	0.0	0.0	**0.2**
HOT121	*Gemella morbillorum*	0.1	0.0	1.4	0.0	0.0	0.0	0.0	0.3	0.0	0.0	0.3	0.0	0.0	**0.2**
HOT885	*Prevotella sp._Oral_Taxon_317*	0.0	0.0	1.0	0.0	0.4	0.8	0.0	0.0	0.0	0.0	0.0	0.0	0.0	**0.2**
HOT275	*Porphyromonas sp._Oral_Taxon_284*	0.0	0.6	0.3	0.0	0.0	0.0	0.0	0.0	0.0	0.0	1.1	0.0	0.1	**0.2**
GGT1789;HOT758	*Streptococcus sp._Oral_Taxon_061*	0.0	0.1	0.1	0.1	0.1	0.1	0.5	0.1	0.2	0.0	0.1	0.1	0.7	**0.2**
GGT1989;HOT524	*Fusobacterium nucleatum_ss_polymorphum*	0.0	0.1	0.3	0.0	0.3	0.2	0.3	0.5	0.2	0.0	0.1	0.0	0.1	**0.2**
GGT1815;HOT073	*Streptococcus intermedius*	0.4	0.0	0.0	0.0	0.7	0.0	0.0	0.0	0.0	0.1	0.0	0.8	0.0	**0.2**
HOT494	*Lactobacillus fermentum*	0.0	0.0	0.0	0.2	0.0	0.0	0.0	0.0	0.0	1.4	0.0	0.0	0.3	**0.2**
GGT62;HOT171	*Actinomyces sp._Oral_Taxon_175*	1.0	0.2	0.2	0.0	0.1	0.0	0.0	0.1	0.1	0.0	0.2	0.0	0.0	**0.1**
GGT1819;HOT411	*Streptococcus sp._Oral_Taxon_056*	0.3	0.0	0.1	0.0	0.0	0.9	0.0	0.0	0.0	0.1	0.3	0.1	0.0	**0.1**
GGT675;HOT118	*Granulicatella elegans*	0.2	0.0	0.1	0.2	0.0	0.0	0.1	0.1	0.1	0.1	0.2	0.1	0.6	**0.1**
GGT1361;HOT807	*Rothia aeria*	0.2	0.3	0.0	0.1	0.0	0.0	0.5	0.0	0.1	0.0	0.4	0.0	0.1	**0.1**
GGT712	*Kingella oralis*	0.1	0.1	0.1	0.0	0.3	0.0	0.0	0.1	0.0	0.0	1.0	0.0	0.0	**0.1**
HOT458	*Cardiobacterium hominis*	0.0	0.0	0.1	0.0	1.0	0.3	0.1	0.0	0.1	0.0	0.0	0.0	0.0	**0.1**
HOT412	*Porphyromonas sp._Oral_Taxon_275*	0.0	0.2	0.0	0.0	0.0	0.0	0.0	1.4	0.0	0.0	0.0	0.0	0.0	**0.1**
GGT1486;HOT714	*Prevotella sp._Oral_Taxon_300*	0.5	0.0	0.4	0.0	0.6	0.0	0.0	0.0	0.0	0.0	0.0	0.2	0.0	**0.1**
HOT300	*Prevotella veroralis*	1.5	0.0	0.1	0.0	0.0	0.0	0.0	0.0	0.0	0.0	0.0	0.0	0.0	**0.1**
HOT286	*Dialister invisus*	1.1	0.0	0.4	0.0	0.0	0.0	0.0	0.0	0.0	0.0	0.0	0.0	0.0	**0.1**
GGT1488;HOT299	*Prevotella sp._Oral_Taxon_G60*	0.0	0.1	0.7	0.1	0.4	0.1	0.0	0.0	0.0	0.0	0.0	0.1	0.0	**0.1**
HOT618	*Actinomyces gerencseriae*	0.0	0.0	0.0	0.0	1.0	0.0	0.1	0.0	0.4	0.0	0.0	0.0	0.0	**0.1**
GGT60;HOT180	*Actinomyces sp._Oral_Taxon_446*	0.0	0.0	0.0	0.0	0.0	0.0	0.0	0.1	0.4	0.2	0.2	0.0	0.6	**0.1**
HOT336	*Porphyromonas catoniae*	0.1	0.0	0.7	0.0	0.0	0.0	0.0	0.3	0.0	0.0	0.3	0.0	0.1	**0.1**
GGT738	*Klebsiella pneumoniae*	0.0	1.4	0.0	0.0	0.0	0.0	0.0	0.0	0.0	0.0	0.0	0.0	0.0	**0.1**
HOT849	*Actinomyces johnsonii*	0.5	0.4	0.0	0.0	0.0	0.0	0.0	0.4	0.0	0.0	0.0	0.0	0.0	**0.1**

GGT816;HOT626	*Lachnoanaerobaculum saburreum*	0.0	0.1	0.2	0.0	0.2	0.2	0.6	0.0	0.0	0.0	0.1	0.0	0.0	**0.1**
GGT54;HOT179	*Atopobium rimae*	0.7	0.0	0.2	0.0	0.1	0.1	0.0	0.0	0.0	0.0	0.0	0.2	0.0	**0.1**
GGT46;HOT176	*Actinomyces naeslundii*	0.0	0.1	0.0	0.2	0.6	0.0	0.0	0.0	0.2	0.1	0.0	0.0	0.0	**0.1**
HOT097	*Selenomonas noxia*	0.0	0.0	0.3	0.0	0.6	0.2	0.1	0.0	0.0	0.0	0.0	0.0	0.0	**0.1**
HOTF78	*Atopobium parvulum*	0.1	0.2	0.3	0.1	0.1	0.0	0.0	0.1	0.0	0.0	0.0	0.2	0.1	**0.1**
GGT808;HOT202	*Leptotrichia hofstadii*	0.0	0.0	0.1	0.0	0.1	0.0	0.8	0.0	0.0	0.0	0.0	0.0	0.1	**0.1**
GGT1988;HOT717	*TM7_[G-1] sp._Oral_Taxon_348*	0.0	0.0	0.7	0.0	0.1	0.0	0.2	0.1	0.0	0.0	0.2	0.0	0.0	**0.1**
HOT313	*Tannerella sp._Oral_Taxon_286*	0.0	0.0	0.3	0.0	0.3	0.2	0.2	0.0	0.1	0.0	0.0	0.0	0.0	**0.1**
GGT1490;HOT289	*Prevotella oris*	1.0	0.0	0.2	0.0	0.0	0.0	0.0	0.0	0.0	0.0	0.0	0.0	0.0	**0.1**
GGT530;HOT595	*Dietzia sp._Oral_Taxon_D12*	0.0	0.0	0.0	0.0	0.0	0.0	0.0	0.0	0.0	0.0	0.0	1.2	0.0	**0.1**
GGT1022;HOT817	*Lactobacillus johnsonii*	0.0	0.0	0.0	0.0	0.9	0.1	0.0	0.0	0.0	0.1	0.0	0.0	0.0	**0.1**
GGT1794;HOT543	*Streptococcus infantis*	0.0	0.1	0.1	0.2	0.0	0.4	0.1	0.0	0.0	0.0	0.1	0.0	0.1	**0.1**
GGT376;HOT575	*Defluvibacter lusatiensis*	0.0	0.0	0.0	0.0	0.0	0.0	0.0	0.0	0.0	0.0	0.0	1.1	0.0	**0.1**
GGT1483;HOT469	*Prevotella oulorum*	0.3	0.0	0.3	0.0	0.2	0.1	0.0	0.0	0.0	0.0	0.0	0.2	0.0	**0.1**
HOTE20	*Streptococcus australis*	0.0	0.0	0.0	0.1	0.0	0.1	0.6	0.0	0.0	0.1	0.0	0.0	0.1	**0.1**
GGT1477;HOT307	*Prevotella sp._Oral_Taxon_313*	0.0	0.0	1.0	0.0	0.0	0.0	0.0	0.0	0.0	0.0	0.0	0.0	0.0	**0.1**
GGT32;HOT554	*Campylobacter concisus*	0.0	0.2	0.2	0.1	0.0	0.0	0.1	0.1	0.0	0.1	0.1	0.0	0.1	**0.1**
HOT064	*Streptococcus sp._Oral_Taxon_431*	0.0	0.1	0.2	0.3	0.0	0.1	0.1	0.0	0.0	0.0	0.0	0.0	0.1	**0.1**
HOT096	*Lactobacillus gasseri*	0.1	0.0	0.0	0.4	0.0	0.0	0.0	0.0	0.0	0.4	0.0	0.0	0.0	**0.1**
HOT322	*Capnocytophaga granulosa*	0.0	0.0	0.3	0.0	0.0	0.1	0.4	0.0	0.0	0.0	0.0	0.0	0.0	**0.1**
HOT389	*Gemella haemolysans*	0.5	0.0	0.3	0.0	0.0	0.0	0.0	0.0	0.0	0.0	0.1	0.0	0.0	**0.1**
HOT900	*Capnocytophaga leadbetteri*	0.0	0.1	0.4	0.0	0.0	0.1	0.3	0.0	0.0	0.0	0.0	0.0	0.0	**0.1**
GGT1479;HOT794	*Prevotella pallens*	0.0	0.0	0.8	0.0	0.0	0.0	0.0	0.0	0.0	0.0	0.0	0.1	0.0	**0.1**
HOT278	*Porphyromonas sp._Oral_Taxon_B43*	0.0	0.1	0.0	0.0	0.0	0.0	0.0	0.0	0.0	0.0	0.7	0.0	0.0	**0.1**
HOT498	*Aggregatibacter sp._Oral_Taxon_458*	0.0	0.1	0.2	0.0	0.0	0.0	0.1	0.1	0.0	0.0	0.3	0.0	0.0	**0.1**
HOTH23	*Streptococcus constellatus*	0.3	0.0	0.5	0.0	0.0	0.0	0.0	0.0	0.0	0.0	0.0	0.0	0.0	**0.1**
HOTA88	*Yersinia mollaretii*	0.0	0.0	0.0	0.0	0.3	0.0	0.2	0.0	0.0	0.1	0.0	0.2	0.0	**0.1**
HOT739	*Rothia mucilaginosa*	0.0	0.1	0.2	0.0	0.0	0.0	0.0	0.1	0.0	0.0	0.1	0.0	0.2	**0.1**
GGT78;HOT485	*Cardiobacterium valvulum*	0.0	0.0	0.1	0.0	0.0	0.6	0.1	0.0	0.0	0.0	0.0	0.0	0.0	**0.1**
HOT326	*Capnocytophaga sputigena*	0.3	0.1	0.0	0.0	0.0	0.1	0.0	0.0	0.0	0.0	0.2	0.0	0.0	**0.1**
HOT912	*Bergeyella sp._Oral_Taxon_322*	0.0	0.0	0.1	0.0	0.0	0.0	0.1	0.1	0.0	0.0	0.3	0.0	0.0	**0.1**
HOT461	*Moryella sp._Oral_Taxon_419*	0.2	0.0	0.4	0.0	0.0	0.0	0.0	0.0	0.0	0.0	0.0	0.0	0.0	**0.1**
HOTE53	*Fusobacterium sp._Oral_Taxon_H27*	0.1	0.0	0.5	0.0	0.0	0.0	0.0	0.0	0.0	0.0	0.0	0.0	0.0	**0.1**

HOT688	*Actinomyces odontolyticus*	0.0	0.1	0.0	0.1	0.0	0.0	0.1	0.1	0.0	0.0	0.1	0.0	0.1	**0.0**
HOT596	*actinoacillus crispatus*	0.0	0.0	0.0	0.0	0.4	0.1	0.0	0.0	0.0	0.0	0.0	0.0	0.0	**0.0**
GGT1026;HOT749	*Mitsuokella sp._Oral_Taxon_131*	0.0	0.0	0.2	0.0	0.0	0.0	0.0	0.0	0.1	0.0	0.0	0.3	0.0	**0.0**
HOT348	*TM7_[G-3] sp._Oral_Taxon_351*	0.4	0.0	0.1	0.0	0.0	0.0	0.0	0.0	0.0	0.0	0.0	0.0	0.0	**0.0**
GGT378;HOT623	*Eikenella corrodens*	0.0	0.0	0.2	0.0	0.2	0.1	0.0	0.0	0.1	0.0	0.0	0.0	0.0	**0.0**
HOT324	*Capnocytophaga sp._Oral_Taxon_412*	0.0	0.0	0.0	0.0	0.0	0.0	0.3	0.0	0.0	0.0	0.2	0.0	0.0	**0.0**
HOT888	*Actinomyces dentalis*	0.0	0.0	0.1	0.0	0.2	0.0	0.0	0.0	0.0	0.0	0.0	0.1	0.0	**0.0**
GGT157;HOT750	*Cryptobacterium curtum*	0.1	0.0	0.1	0.0	0.1	0.0	0.0	0.0	0.0	0.0	0.0	0.2	0.0	**0.0**
HOT061	*Streptococcus sp._Oral_Taxon_074*	0.0	0.1	0.1	0.0	0.0	0.1	0.0	0.0	0.0	0.1	0.0	0.0	0.2	**0.0**
GGT1027;HOT819	*Lactobacillus sp._Oral_Taxon_461*	0.5	0.0	0.0	0.0	0.0	0.0	0.0	0.0	0.0	0.0	0.0	0.0	0.0	**0.0**
GGT573;HOT579	*Olsenella sp._Oral_Taxon_807*	0.0	0.0	0.1	0.0	0.1	0.0	0.2	0.0	0.0	0.0	0.0	0.0	0.0	**0.0**
GGT1025;HOT716	*Lactobacillus vaginalis*	0.0	0.0	0.0	0.0	0.0	0.0	0.0	0.0	0.0	0.5	0.0	0.0	0.0	**0.0**
GGT1807;HOT638	*Streptococcus parasanguinis_I*	0.0	0.0	0.1	0.1	0.0	0.0	0.0	0.2	0.0	0.0	0.0	0.0	0.0	**0.0**
GGT1043;HOT756	*Moryella sp._Oral_Taxon_097*	0.1	0.1	0.2	0.0	0.0	0.0	0.0	0.0	0.0	0.0	0.0	0.0	0.0	**0.0**
GGT1570;HOT854	*TM7_[G-1] sp._Oral_Taxon_347*	0.0	0.0	0.0	0.0	0.3	0.1	0.0	0.0	0.0	0.0	0.0	0.0	0.0	**0.0**
GGT380;HOT763	*Enterobacter cloacae*	0.0	0.0	0.0	0.0	0.0	0.0	0.3	0.0	0.0	0.0	0.0	0.0	0.0	**0.0**
GGT1546	*Ralstonia pickettii*	0.0	0.0	0.0	0.0	0.2	0.0	0.0	0.0	0.0	0.0	0.0	0.0	0.0	**0.0**
GGT711	*Kingella denitrificans*	0.1	0.0	0.0	0.0	0.0	0.0	0.1	0.0	0.0	0.0	0.0	0.0	0.1	**0.0**
GGT807;HOT420	*Leptotrichia buccalis*	0.0	0.0	0.1	0.0	0.1	0.0	0.1	0.0	0.0	0.0	0.0	0.0	0.0	**0.0**
HOT323	*Capnocytophaga sp._Oral_Taxon_336*	0.0	0.1	0.1	0.0	0.0	0.0	0.1	0.0	0.0	0.0	0.0	0.0	0.0	**0.0**
GGT1037;HOT818	*Megasphaera micronuciformis*	0.0	0.0	0.1	0.0	0.1	0.0	0.0	0.0	0.0	0.0	0.0	0.0	0.0	**0.0**
GGT387;HOT700	*Capnocytophaga sp._Oral_Taxon_326*	0.1	0.0	0.1	0.0	0.1	0.1	0.0	0.0	0.0	0.0	0.0	0.0	0.0	**0.0**
HOT221	*Acinetobacter sp._Oral_Taxon_A58*	0.0	0.0	0.0	0.0	0.4	0.0	0.0	0.0	0.0	0.0	0.0	0.0	0.0	**0.0**
GGT1432;HOT111	*Staphylococcus equorum*	0.0	0.0	0.4	0.0	0.0	0.0	0.0	0.0	0.0	0.0	0.0	0.0	0.0	**0.0**
GGT306	*Gemella sanguinis*	0.0	0.0	0.0	0.0	0.0	0.0	0.0	0.0	0.0	0.0	0.0	0.0	0.2	**0.0**
GGT550;HOT666	*Olsenella profusa*	0.0	0.1	0.1	0.0	0.0	0.0	0.0	0.0	0.0	0.0	0.0	0.0	0.0	**0.0**
GGT1312;HOT764	*Pseudomonas mosselii_Oral_Taxon_A88*	0.0	0.0	0.0	0.0	0.0	0.4	0.0	0.0	0.0	0.0	0.0	0.0	0.0	**0.0**
GGT1041;HOT051	*Oribacterium sinus*	0.0	0.1	0.1	0.0	0.0	0.0	0.0	0.0	0.0	0.0	0.0	0.0	0.0	**0.0**
GGT1302;HOT729	*Pseudomonas fluorescens*	0.0	0.0	0.0	0.0	0.0	0.0	0.2	0.0	0.0	0.1	0.0	0.1	0.0	**0.0**
HOT329	*Capnocytophaga sp._Oral_Taxon_324*	0.0	0.1	0.0	0.0	0.2	0.0	0.0	0.0	0.0	0.0	0.0	0.0	0.0	**0.0**
GGT1114;HOT122	*Oribacterium sp._Oral_Taxon_078*	0.2	0.0	0.0	0.0	0.0	0.0	0.0	0.0	0.0	0.0	0.0	0.0	0.0	**0.0**
GGT1362;HOT806	*Propionibacterium propionicum*	0.0	0.0	0.0	0.0	0.0	0.0	0.0	0.0	0.1	0.0	0.2	0.0	0.0	**0.0**

HOT887	*Fusobacterium periodonticum*	0.0	0.0	0.1	0.0	0.0	0.0	0.0	0.0	0.0	0.0	0.0	0.0	0.0	**0.0**
GGT56	*Corynebacterium durum*	0.0	0.0	0.0	0.0	0.0	0.0	0.0	0.0	0.0	0.0	0.1	0.0	0.1	**0.0**
HOTF11	*Streptococcus vestibularis*	0.1	0.0	0.0	0.0	0.0	0.0	0.0	0.0	0.1	0.1	0.0	0.0	0.0	**0.0**
HOT070	*Streptococcus sp._Oral_Taxon_E78*	0.0	0.0	0.0	0.0	0.0	0.1	0.0	0.0	0.0	0.0	0.0	0.0	0.0	**0.0**
HOTB43	*Prevotella maculosa*	0.2	0.0	0.0	0.0	0.1	0.0	0.0	0.0	0.0	0.0	0.0	0.0	0.0	**0.0**
GGT1624;HOT587	*Alloprevotella sp._Oral_Taxon_473*	0.1	0.0	0.1	0.0	0.0	0.0	0.0	0.0	0.0	0.0	0.0	0.0	0.0	**0.0**
HOT473	*Alloprevotella tannerae*	0.0	0.0	0.3	0.0	0.0	0.0	0.0	0.0	0.0	0.0	0.0	0.0	0.0	**0.0**
GGT1000;HOT582	*Neisseria elongata*	0.0	0.0	0.0	0.0	0.0	0.0	0.1	0.0	0.0	0.0	0.0	0.0	0.0	**0.0**
HOT874	*TM7_[G-1] sp._Oral_Taxon_352*	0.0	0.0	0.1	0.0	0.0	0.0	0.0	0.0	0.0	0.0	0.0	0.0	0.1	**0.0**
GGT1308;HOT476; HOT610	*Pseudomonas aeruginosa*	0.0	0.0	0.0	0.0	0.0	0.0	0.0	0.0	0.0	0.0	0.0	0.2	0.0	**0.0**
HOTG62	*Veillonella atypica*	0.0	0.0	0.1	0.0	0.0	0.0	0.0	0.0	0.0	0.1	0.0	0.0	0.0	**0.0**
GGT815;HOT046	*Lachnospiraceae_[G-2] sp._Oral_Taxon_096*	0.0	0.1	0.1	0.0	0.0	0.0	0.0	0.0	0.0	0.0	0.0	0.0	0.0	**0.0**
GGT1784;HOT734	*Streptococcus sp._Oral_Taxon_057*	0.0	0.0	0.0	0.0	0.0	0.1	0.0	0.0	0.0	0.1	0.0	0.0	0.0	**0.0**
GGT1034	*Lactobacillus salivarius*	0.0	0.0	0.0	0.0	0.0	0.0	0.0	0.0	0.0	0.2	0.0	0.0	0.0	**0.0**
HOT305	*Riemerella anatipestifer*	0.0	0.0	0.0	0.0	0.0	0.0	0.0	0.0	0.0	0.0	0.0	0.0	0.2	**0.0**
HOT078	*Selenomonas sputigena*	0.0	0.0	0.0	0.0	0.0	0.0	0.0	0.0	0.0	0.0	0.0	0.1	0.0	**0.0**
HOTE78	*Streptococcus sp._str._M334*	0.0	0.0	0.0	0.1	0.0	0.0	0.0	0.0	0.0	0.0	0.0	0.0	0.0	**0.0**
HOT222	*Agrobacterium tumefaciens*	0.0	0.0	0.0	0.0	0.0	0.0	0.0	0.0	0.0	0.0	0.0	0.2	0.0	**0.0**
GGT1473;HOT288	*Prevotella sp._Oral_Taxon_299*	0.0	0.1	0.0	0.0	0.0	0.0	0.0	0.0	0.0	0.0	0.1	0.0	0.0	**0.0**
GGT1809	*Veillonella denticariosi*	0.0	0.0	0.2	0.0	0.0	0.0	0.0	0.0	0.0	0.0	0.0	0.0	0.0	**0.0**
GGT392;HOT775	*Porphyromonas sp._Oral_Taxon_278*	0.0	0.0	0.2	0.0	0.0	0.0	0.0	0.0	0.0	0.0	0.0	0.0	0.0	**0.0**
HOT913	*Bergeyella sp._Oral_Taxon_900*	0.0	0.0	0.0	0.0	0.1	0.0	0.1	0.0	0.0	0.0	0.0	0.0	0.0	**0.0**
HOT419	*Selenomonas sp._Oral_Taxon_E20*	0.0	0.0	0.2	0.0	0.0	0.0	0.0	0.0	0.0	0.0	0.0	0.0	0.0	**0.0**
HOT347	*TM7_[G-1] sp._Oral_Taxon_870*	0.0	0.0	0.2	0.0	0.0	0.0	0.0	0.0	0.0	0.0	0.0	0.0	0.0	**0.0**
HOT349	*Treponema socranskii_ss_socranskii*	0.1	0.0	0.1	0.0	0.0	0.0	0.0	0.0	0.0	0.0	0.0	0.0	0.0	**0.0**
GGT33	*Campylobacter showae*	0.0	0.0	0.1	0.0	0.0	0.0	0.0	0.0	0.0	0.0	0.0	0.0	0.0	**0.0**
GGT1074;HOT563	*Leptotrichia sp._Oral_Taxon_221*	0.0	0.1	0.0	0.0	0.0	0.0	0.0	0.0	0.0	0.0	0.0	0.0	0.0	**0.0**
HOT100	*Lactobacillus helveticus*	0.0	0.0	0.0	0.0	0.1	0.0	0.0	0.0	0.0	0.0	0.0	0.0	0.0	**0.0**
GGT2038	*TM7_[G-1] sp._Oral_Taxon_349*	0.0	0.0	0.1	0.0	0.0	0.0	0.0	0.0	0.0	0.0	0.0	0.0	0.0	**0.0**
HOT298	*Prevotella multisaccharivorax*	0.0	0.0	0.2	0.0	0.0	0.0	0.0	0.0	0.0	0.0	0.0	0.0	0.0	**0.0**
GGT1367;HOT457	*Selenomonas sp._Oral_Taxon_H23*	0.0	0.0	0.0	0.0	0.1	0.0	0.0	0.0	0.0	0.0	0.0	0.0	0.0	**0.0**

HOTH27	*Leptotrichia sp._Oral_Taxon_215*	0.0	0.0	0.1	0.0	0.0	0.0	0.0	0.0	0.0	0.0	0.0	0.0	0.0	**0.0**
GGT813;HOT757	*Lachnospiraceae_[G-3] sp._Oral_Taxon_100*	0.0	0.0	0.1	0.0	0.0	0.0	0.0	0.0	0.0	0.0	0.0	0.0	0.0	**0.0**
HOT074	*Streptococcus sp._Oral_Taxon_G62*	0.0	0.0	0.0	0.0	0.0	0.0	0.0	0.0	0.0	0.0	0.0	0.1	0.0	**0.0**
HOT131	*Parvimonas micra*	0.1	0.0	0.0	0.0	0.0	0.0	0.0	0.0	0.0	0.0	0.0	0.0	0.0	**0.0**
GGT388;HOT325	*Capnocytophaga sp._Oral_Taxon_323*	0.0	0.0	0.0	0.0	0.1	0.0	0.0	0.0	0.0	0.0	0.0	0.0	0.0	**0.0**
HOT448	*Actinomyces sp._Oral_Taxon_E63*	0.0	0.0	0.1	0.0	0.0	0.0	0.0	0.0	0.0	0.0	0.0	0.0	0.0	**0.0**
HOT417	*Acinetobacter sp._str._DR1*	0.0	0.0	0.0	0.0	0.0	0.0	0.0	0.0	0.0	0.0	0.0	0.0	0.1	**0.0**
HOT317	*Tannerella sp._Oral_Taxon_808*	0.0	0.0	0.1	0.0	0.0	0.0	0.0	0.0	0.0	0.0	0.0	0.0	0.0	**0.0**
GGT806;HOT201	*Leptotrichia shahii*	0.0	0.0	0.1	0.0	0.0	0.0	0.0	0.0	0.0	0.0	0.0	0.0	0.0	**0.0**
HOT195	*Alloprevotella sp._Oral_Taxon_913*	0.0	0.0	0.1	0.0	0.0	0.0	0.0	0.0	0.0	0.0	0.0	0.0	0.0	**0.0**
HOT609	*Ochrobactrum anthropi*	0.0	0.0	0.0	0.0	0.0	0.0	0.0	0.0	0.0	0.0	0.0	0.1	0.0	**0.0**
GGT1991;HOT158; HOT160;HOT161	*Fusobacterium sp._Oral_Taxon_203*	0.0	0.0	0.1	0.0	0.0	0.0	0.0	0.0	0.0	0.0	0.0	0.0	0.0	**0.0**
GGT391;HOT337	*Capnocytophaga ochracea*	0.0	0.0	0.0	0.0	0.0	0.1	0.0	0.0	0.0	0.0	0.0	0.0	0.0	**0.0**
HOT071	*Streptococcus sp._Oral_Taxon_F11*	0.0	0.0	0.0	0.0	0.0	0.0	0.0	0.0	0.0	0.0	0.0	0.0	0.1	**0.0**
GGT1031;HOT615	*Lactobacillus rhamnosus*	0.0	0.0	0.0	0.0	0.0	0.0	0.0	0.0	0.0	0.0	0.0	0.0	0.1	**0.0**
GGT1035;HOT608	*Lactobacillus reuteri*	0.0	0.0	0.0	0.0	0.1	0.0	0.0	0.0	0.0	0.0	0.0	0.0	0.0	**0.0**
GGT1051;HOT022	*Neisseria pharyngis*	0.0	0.0	0.0	0.0	0.0	0.0	0.0	0.0	0.0	0.0	0.0	0.0	0.1	**0.0**
GGT1608	*Brevibacillus brevis*	0.0	0.0	0.0	0.0	0.1	0.0	0.0	0.0	0.0	0.0	0.0	0.0	0.0	**0.0**
GGT731	*Kingella sp._Oral_Taxon_C21*	0.0	0.0	0.0	0.0	0.0	0.0	0.0	0.0	0.0	0.0	0.1	0.0	0.0	**0.0**
GGT1533	*SR1_[G-1] sp._Oral_Taxon_874*	0.0	0.0	0.1	0.0	0.0	0.0	0.0	0.0	0.0	0.0	0.0	0.0	0.0	**0.0**
HOT303	*Prevotella sp._Oral_Taxon_305*	0.0	0.0	0.0	0.1	0.0	0.0	0.0	0.0	0.0	0.0	0.0	0.0	0.0	**0.0**
GGT1478;HOT693	*Prevotella pleuritidis*	0.0	0.0	0.1	0.0	0.0	0.0	0.0	0.0	0.0	0.0	0.0	0.0	0.0	**0.0**
GGT1476;HOT643	*Prevotella nigrescens*	0.1	0.0	0.0	0.0	0.0	0.0	0.0	0.0	0.0	0.0	0.0	0.0	0.0	**0.0**
GGT1482;HOT572	*Anaeroglobus geminatus*	0.0	0.0	0.1	0.0	0.0	0.0	0.0	0.0	0.0	0.0	0.0	0.0	0.0	**0.0**
GGT1627;HOT681	*Alloprevotella sp._Oral_Taxon_912*	0.0	0.0	0.1	0.0	0.0	0.0	0.0	0.0	0.0	0.0	0.0	0.0	0.0	**0.0**
HOT808	*Dialister pneumosintes*	0.0	0.0	0.1	0.0	0.0	0.0	0.0	0.0	0.0	0.0	0.0	0.0	0.0	**0.0**
HOT346	*TM7_[G-1] sp._Oral_Taxon_353*	0.0	0.0	0.1	0.0	0.0	0.0	0.0	0.0	0.0	0.0	0.0	0.0	0.0	**0.0**
	**Column Sum**	**99.0**	**99.6**	**98.1**	**99.7**	**99.4**	**98.5**	**99.4**	**99.5**	**99.8**	**99.8**	**99.7**	**99.3**	**99.7**	**100**

[a]*Species observed in just one of the total patients, not representing a common data among the studied population*
