# Supplementary material for: The oral bacterial microbiome of occlusal surfaces in children and its association with diet and caries
Source: PLoS One. 2017 Jul 5;12(7):e0180621. doi: 10.1371/journal.pone.0180621 (PMC5498058; doi:10.1371/journal.pone.0180621)
Supplement: S4 Table — (PDF) [file pone.0180621.s006.pdf]

The Oral Bacterial Microbiome of Occlusal Surfaces in Children and its Association with Diet And Caries

S4 Table. Mean relative abundances (RA) of all species represented over 0.1%, by individual biofilm sample.

Relative abundance ≧0.1		Sound occlusal surfaces (bacterial microbiome from each surface sampled)																					
**Taxon_ID**	**Species**	**117**	**237**	**317**	**337**	**347**	**517**	**527**	**537**	**547**	**637**	**647**	**717**	**747**	**837**	**847**	**947**	**1037**	**1047**	**1147**	**1327**	**1337**	**1347**
**GGT1;HOT534**	*Granulicatella paradiacens*	5.2	9.2	3.5	19.3	3.1	16.6	29.7	11.1	47.7	30.5	4.5	9.8	5.7	23	27.6	20.6	9.3	29.6	0.9	5.8	11.9	11.4
**GGT1799;HOT686**	*Streptococcus mutans*	6.9	26.2	0.6	1	5.8	0.3	3.9	19.8	5.3	11	1.5	0.4	0.3	2.8	2.8	5.7	26.1	10.8	0.9	0.8	3.6	55.2
**GGT1822**	*Streptococcus sp._str._C300*	2.4	4.1	7.4	17	14.9	19.6	7.3	4.9	4.2	3.3	5.3	5.1	0.7	4.2	11.1	6	5.5	2.8	0.4	1.1	3.3	0.8
**GGT1051;HOT022**	*Lautropia mirabilis*	2.8	2.2	30.4	12.7	21.9	6	2.8	1.8	2.7	2.5	5.8	9.5	4.1	3.3	0.6	5	0.5	2.5	0.2	0	4.1	1.2
**HOTC61**	*Pseudomonas sp._Oral_Taxon_C61*	0	0	0	0	0	0	0	0	0	0	0	0	39.1	0	0	0	0	0	39.3	0	0	0
**GGT1803;HOT622**	*Streptococcus gordonii*	1.7	5.5	0.6	1.5	0.4	6.7	8.7	7.2	4	5.4	1.1	2.2	0.1	3.3	4.1	4.8	11.4	8.6	0	0.1	3.9	4.46
**GGT1789;HOT758**	*Streptococcus sanguinis*	0.7	0.3	5.8	2.6	4.6	3.3	6	4	2.6	3.3	9.7	4.5	1.7	3.2	3.2	1.9	5.8	2.6	0.7	0.4	1.2	2.21
**HOT389**	*Abiotrophia defectiva*	0.8	0.4	0.9	4.3	0.3	4.8	1.2	1.7	2	4.7	0	0	0	0.3	0.9	0.2	0.4	0.5	0	0.1	11.5	0.43
**GGT1806;HOT707**	*Streptococcus oralis*	0	0	0.1	0.6	0.1	0.1	7.5	3.7	0.5	7.7	16.8	14.1	0	0	0.1	3.1	4.2	1.7	0	0	10.8	0
**HOTE53**	*Veillonella sp._Oral_Taxon_E53*	1.4	4.1	7.1	1.6	8.8	3.1	1.2	1.9	2.7	1	0.6	4.8	3.7	1.6	0.1	2	1.5	1.7	1.7	14.2	6.6	2.46
**GGT1533**	*Pseudomonas putida*	0	0	0	0	0	0	0	0	0	0	36.2	0	0	0	0	0	0	0	0	0	0	0
**GGT1312;HOT764**	*Neisseria sicca*	0.1	0.1	0.6	4.1	0	0.1	0	0	0	0	0	0	2	17.2	35	0	0	0	0	0	0	0
**GGT1991;HOT158;HOT160;HOT161**	*Veillonella parvula_group*	0.7	2.4	4.6	1.6	6.5	1	0.6	0.8	1.1	0.5	0.7	5.5	1.7	0.7	0.1	0.9	0.4	0.3	2.6	15.5	2.8	1.82
**GGT738**	*Escherichia coli*	0	0	0	0	0	0	0	0	0	0	0.1	0.1	0	0	0	0	0	0	0	0	0	0
**GGT32;HOT554**	*Acinetobacter baumannii*	0	0	0	0	0	0	1.2	0	0	0	0	0	0	0	0	0	0	0	0	0	0	0
**HOT064**	*Streptococcus sp._Oral_Taxon_064*	0	0.1	0.1	0.1	0.1	0.2	14.7	9.6	4.4	13.8	1	1.5	0	3.9	3.1	0.2	0.1	0	0	0	0.1	0.16

**GGT1784;HOT734**	*Streptococcus pneumoniae*	0	0.3	0.1	0.5	0.1	0.5	0.4	0.8	0.2	0.7	0.1	0.3	0.1	0.2	0.7	22.1	1.9	7.9	0.9	0.1	0.3	0.02
**GGT1484;HOT291**	*Prevotella denticola*	6.5	5.6	0	0	0	0	0	0.5	0.9	0	0	0	0.2	0	0	0	2	0.3	0	0	1.4	0.01
**GGT712**	*Enterobacter sp._str._638*	0	0	0	0	0	0	0	0	0	0	0	0.6	4.9	0	0	0	0	0	0	0	0	0
**GGT62;HOT171**	*Actinomyces sp._Oral_Taxon_171*	0	0	4.4	0.2	11	0.2	0.7	2.3	1.9	1	0.2	0.2	0.1	0	0	0.2	0.1	0.1	0.9	8.4	1.1	0.59
**GGT1809**	*Streptococcus sp._str._2136FAA*	0.1	0.1	0	0	0	0	0	0	0	0	0	0	0	0	0	2.1	18.7	9.3	2	3.3	0.1	0
**GGT1791;HOT578**	*Streptococcus cristatus*	0	0.1	1.1	5.5	0	5.7	0.4	0.8	0.6	0.6	0.5	1	2.1	1.7	0.8	0.9	2	1.3	0.1	0.1	4	3.18
**GGT1787;HOT398;HOT677**	*Streptococcus mitis*	0	0.4	0.4	0.4	0.1	2	0.7	1.2	0.5	0.3	0.8	4.2	0.6	2.4	1.2	0.3	1.1	1.3	0.3	0.3	3.7	0.19
**GGT1795;HOT755**	*Streptococcus salivarius*	0	0	0.1	0.1	0.1	0.4	2.7	4.8	0.5	2	0	0.1	0.1	0	0.1	0	0.4	0	1.3	3.4	0.1	0.19
**HOT071**	*Streptococcus sp._Oral_Taxon_071*	1.1	1.3	0	0.9	0	0.7	0.2	0.2	0.3	0.4	0.1	2.4	0.3	0	0	3.2	0.2	0.1	1.1	1.7	0.7	1.45
**GGT1819;HOT411**	*Streptococcus parasanguinis_II*	0	0	0	0	0.1	0.6	0.6	2.6	0.3	0.7	0	0.3	0.1	0.2	0.7	0	0.8	0.3	2.5	6.1	0.1	0.46
**HOT170**	*Actinomyces sp._Oral_Taxon_170*	0	0.1	9.8	1.9	4.1	0.3	0.2	0.2	0.2	0.1	2.5	4.6	0	0	0	0	0.1	0	0	0	1.4	0.02
**HOT768**	*Streptococcus sobrinus*	0	0	0	0.3	2.1	0	0	0	0	0	0	0	0	0	0	0	0	0	4.7	17.5	0	0
**GGT1483;HOT469**	*Prevotella melaninogenica*	8.1	6.4	0.2	0.7	0	1.8	0	0.6	2.2	0	0	0.9	0.1	0.1	0.1	0.1	0	0	0	0.1	0.1	0.23
**GGT1300;HOT682**	*Neisseria mucosa*	0	0.1	0.1	0.4	0	0.5	0	0.1	0	0	0	0	0.1	0.1	0.2	3.1	0.2	2	0	0	4.6	0.8
**HOT448**	*Actinomyces sp._Oral_Taxon_448*	0	0.2	0.2	0	0.2	0.2	2.1	3	1.9	3.5	0	0.5	0.4	0.1	0	0.8	0.9	5.6	0.4	1.2	1.3	0.03
**HOT169**	*Actinomyces sp._Oral_Taxon_169*	0.1	0.8	0.9	0.3	0.5	0.2	0.4	0	0	0	0	0	0	0.2	0.4	1.9	0.2	0.3	0.2	0.4	0.4	2.19
**GGT1025;HOT716**	*Lactobacillus paracasei*	0.1	0	0.2	0	0	0	0	0	0	0	0	0	0	0	0.1	0	0	0	0	0	0	0.04
**GGT1988;HOT717**	*Variovorax paradoxus*	26	0	0	0	0	0	0	0	0	0	0	0	0	0	0	0	0	0	0	0	0	0

**HOT070**	*Streptococcus sp._Oral_Taxon_070*	0	0.8	0	0	0	0.7	0.1	0.3	0.1	0.1	0	0	0	0	0.2	0	0	0	0	0	0	0.01
**HOT058**	*Streptococcus sp._Oral_Taxon_058*	0	0	0.4	0.9	0.1	1.6	0.3	0.1	0	0	0	0	1.1	0.5	0.2	0.2	1	0.2	0.1	0	0.3	0.48
**GGT378;HOT623**	*Campylobacter gracilis*	1.3	1.3	0.1	0.2	0	0.1	0	0.7	0.6	0.1	0.5	2.4	0.7	0.5	0	1	0.4	0.9	0.5	3	0.2	0.07
**GGT866;HOT718;HOT826**	*Haemophilus parainfluenzae*	0.3	0.4	4.4	1.5	3.2	1.2	0	0.2	0	0	0.1	0.4	0.3	0.4	0.3	0.1	0.1	0.1	0.2	0.2	0.5	0.14
**GGT1308;HOT476;HOT610**	*Neisseria flavescens|subflava*	0	0.1	0.2	2.5	0.1	0.1	0	0.1	0.1	0	0	0.1	1.4	6.6	1.7	0.1	0	0	0	0	0.3	0.37
**GGT1546**	*Pseudomonas antarctica*	0	0	0	0	0	0	0	0	0	0	0	0	0	0	0	0	0	0	19.8	0	0	0
**GGT807;HOT420**	*Fusobacterium nucleatum_ss_animalis*	12.4	2.4	0	0.1	0	0.1	0	0.2	0	0	0	0	0	0	0	0.1	0	0	0.1	0	0.1	0
**HOT191**	*Propionibacterium acidifaciens*	0	0	0.2	0	0.3	0	0.2	2.7	2.6	0.9	0	0	0.1	0	0	3.1	0.5	2.1	0.1	0.2	0	0
**HOT065**	*Streptococcus sp._Oral_Taxon_065*	0	0	0.4	0.3	0.4	0.8	0	1	0.3	0.1	0.1	0.3	0.1	0.1	0.8	0	0	0	0	0.3	0.1	0.34
**HOT279**	*Porphyromonas sp._Oral_Taxon_279*	0	0	0.1	0.3	0	1.5	0	0	0	0	0.1	0.1	3	5.8	0.2	0	0.1	0.1	0	0	1.3	0.06
**GGT550;HOT666**	*Corynebacterium matruchotii*	0	0	2	1.2	0.1	0.7	0.8	0.8	0.5	0.2	0	0.1	2.1	1	0.1	0.4	0.1	0.2	0	0	1.2	0.53
**GGT60;HOT180**	*Actinomyces sp._Oral_Taxon_180*	0.4	0.4	2.1	1.1	1.1	0.7	0	0.1	0	0	0.1	0.1	0.1	0.1	0.1	0	0	0	0	0	0.1	0.01
**HOT195**	*Scardovia wiggsiae*	0.3	0.2	0.2	0	0.3	0	0.2	0.3	1.3	0.5	0.1	0.6	0	0	0	0.1	0.1	0.6	0.7	6.7	0	0.61
**GGT1075**	*Leptotrichia sp._AF189244.1*	0	0.1	0.6	0.6	0	0.2	0.4	0.5	0.2	0.3	0.1	0.5	0.3	0.3	0.1	0.1	0	0.1	0	0.1	2.4	0.35
**GGT731**	*Erwinia aphidicola*	0	0	0	0	0	0	0	0	0	0	0	0	0	0	0	0	0	0	0	0	0	0
**HOTB66**	*Streptococcus sp._Oral_Taxon_B66*	0	0	0.7	0.8	0.4	0	0	0.1	0.1	0	0	0.1	0.2	0	0	0	0	0	0	0	0.5	0
**HOT298**	*Prevotella histicola*	1.9	2.7	0.1	0.3	0.1	0	0.5	0.1	0.5	0.1	0	0.2	0	0.1	0.1	0.2	0	0.1	0	0.5	0.3	1.49
**HOT417**	*Leptotrichia*	0	0	0.2	1.3	0	1	0	0.1	0	0	0	0.4	2.9	0.3	0	0	0	0.1	0	0	0.6	0.22

	sp._Oral_Taxon _417																						
**GGT56**	*Actinomyces viscosus*	0	0.4	0.3	0.2	0.1	0	0.6	0.6	0.2	0.3	0	0	0	0	0	1	0.1	0.3	0	0	0.3	0.14
**GGT391;HOT337**	*Capnocytophaga gingivalis*	0	0	0.6	0.2	0	0.2	0	0	0	0	0.3	1.3	2.3	0.3	0	0	0	0.1	0	0	0.4	0.01
**GGT1023**	*Lactobacillus delbrueckii*	0	0	0	0	0	0	0	0	0	0	0	0	0	0	0	0	0	0	11.2	0.2	0	0
**HOT222**	*Leptotrichia wadei*	0	0.4	0.1	1.4	0	0.5	0	0.2	0.1	0.1	0	0	0.1	0.1	0	0.1	0.1	0.3	0	0.1	0.4	0
**HOT183**	*Actinobaculum sp._Oral_Taxon _183*	0	0	0	0	0	0	0.3	0.6	2.3	0.1	2.3	0.2	0.2	0	0	3.2	1	0.7	0	0.1	0	0
**GGT815;HOT046**	*Gemella morbillorum*	0.2	0.1	0	0.1	0	1.5	0	0	0	0	0	0	0	1.2	0.1	0	0	0	0	0	0.5	0.02
**GGT1477;HOT307**	*Prevotella salivae*	0.1	0.7	0	0.2	0	0	0	0.1	0.9	0	0	0	0	0	0	0.1	0	0	0	0.1	0.1	0.09
**HOT893**	*Actinomyces oris*	0	0	2.2	0.1	5.2	0	0	0	0.1	0.1	0	0	0	0	0	0	0	0	0	0	0.1	0
**HOT346**	*TM7_[G-1] sp._Oral_Taxon _346*	0.2	1.1	0	0.4	0	0.1	0	0.1	0	0	0	0	0.7	0	0	0	0	0	0	0	0	0
**HOT317**	*Prevotella sp._Oral_Taxon _317*	0	0	0	0	0	0.5	0	0	0	0	0.2	3.9	0	0.1	0	0	0	0	0	0	0.1	0
**GGT1624;HOT587**	*Rothia dentocariosa*	0	0	0.1	0.2	0.4	1.1	1.4	0.4	0.1	0.4	0	0	0	0	0.1	0.8	0.2	0.1	0.2	0.2	0.2	0.11
**HOT609**	*Neisseria flava*	0.3	2.5	0.1	0.1	0	0	0	0.1	0.1	0	0	0	0	0.8	0.8	0	0	0	0	0	0	0.06
**HOT284**	*Porphyromonas sp._Oral_Taxon _284*	0	0	0.5	1.9	0	0.8	0	0	0	0	0	0	0	0	0	0	0	0	0	0	1.8	0.14
**GGT808;HOT202**	*Fusobacterium nucleatum_ss_ polymorphum*	0	0	0.1	0.2	0	0.4	0	0	0	0	0.1	0.8	0.7	1.8	0.1	0.2	0.1	0.4	0	0	0.1	0.38
**GGT1794;HOT543**	*Streptococcus anginosus*	3.4	1.3	0	0	0	0	0	0	0	0	0.1	0.3	0	0	0.1	0	0	0	0	0	0	0.01
**HOT498**	*Leptotrichia sp._Oral_Taxon _498*	0	0	0	0	0	0	0	0	0	0	0	0	0.1	0	0	0	0	0	0	0	0	0
**GGT1807;HOT638**	*Streptococcus infantis*	0	0	0.1	0.1	0.1	0.3	0	0.9	0.1	0.1	0.2	0.8	0	0	0	0	0	0	0	0	0.1	0.02
**HOT061**	*Streptococcus sp._Oral_Taxon _061*	0	0	0.1	0.1	0.1	0.2	0	0.6	0.1	0.1	0	0.2	0.1	0	0.1	0	0.2	0.6	0	0	0	0.3

**HOT283**	*Porphyromonas catoniae*	0	0	0	0	0	1.7	0	0	0	0	0.1	0	0	0.5	0.2	0	0	0	0	0	0.5	0.11
**HOT275**	*Porphyromonas sp._Oral_Taxon _275*	0	0	0.2	0.6	0	0	0	0	0	0	0	0	0	5.7	0	0	0	0	0	0	0.1	0
**GGT1035;HOT608**	*Lactobacillus fermentum*	0	0	0	0	0	0	0.1	0.1	0.2	0.4	0	0	0	0	0	0	0	0	2.8	1.8	0.1	0.35
**HOT446**	*Actinomyces sp._Oral_Taxon _446*	0	0	0	0	0	0	0	0	0	0	0	0	0	0.1	0.1	0.6	0.5	0.6	0.1	1	0.3	1.1
**HOT348**	*TM7_[G-1] sp._Oral_Taxon _348*	0	0	0	0.2	0	0.2	0	0	0	0	0	0	0.5	0.3	0	0	0	0	0	0	0.7	0
**GGT1625;HOT188**	*Rothia aeria*	0	0	0.2	0.1	0.8	0.1	0.2	0.2	0.1	0.1	0.1	0	1	0	0	0.1	0.1	0.1	0	0	0.5	0.05
**HOT056**	*Streptococcus sp._Oral_Taxon _056*	0	0	0	0	0	0	0.1	0.1	0	0	2.3	0.6	0.1	0	0.1	0	0.1	0	0	0	0.2	0
**HOT175**	*Actinomyces sp._Oral_Taxon _175*	1	1.4	0.3	0.1	0.2	0.3	0	0.1	0	0	0	0	0	0.2	0.1	0.1	0	0	0	0	0.2	0.01
**GGT999;HOT706**	*Kingella oralis*	0	0.1	0.2	0	0	0	0	0	0	0	0	0	0	0	0	0.1	0	0	0	0	0.8	0.09
**GGT397;HOT633**	*Cardiobacterium hominis*	0	0	0	0	0	0	0	0	0	0	0.3	0.9	0.4	0	0	0.3	0	0.1	0	0	0	0
**GGT1002;HOT731**	*Klebsiella pneumoniae*	0	0	0	0	0	0	0	0	0	0	0	0	0	0	0	0	0	0	0	0	0	0
**HOT618**	*Actinomyces gerencseriae*	0	0	0	0	0	0	0	0	0	0	0	0.1	0.3	0	0	0.8	0	0	0	0	0	0
**HOT849**	*Actinomyces johnsonii*	0	0	0.2	1	0	0	0	0	0	0	0	0	0.1	1.4	0.2	0	0	0	0	0	0	0.06
**HOT596**	*Granulicatella elegans*	0.1	0.4	0	0	0	0.1	0.1	0.2	0.2	0.4	0	0	0	0	0.2	0.2	0.1	0.2	0	0	0.2	0.05
**GGT1808;HOT644**	*Streptococcus intermedius*	0.5	0.4	0	0	0.1	0	0	0	0	0	0	0.1	0	0	0	0	0	0	0.1	0.2	0.1	0
**HOTE75**	*Actinomyces sp._Oral_Taxon _E75*	0.4	0.5	0	0	0	0	0	0	0	0	0.2	0.2	0	0	0	0	0	0	0	0	0	0
**GGT46;HOT176**	*Actinomyces naeslundii*	0	0	0.1	0	0.1	0.1	0.2	0.1	0.1	0.5	0.1	0	0.1	0.1	0	0.2	0.1	0.5	0	0.1	0.1	0.02
**GGT1027;HOT819**	*Lactobacillus johnsonii*	0	0	0	0	0	0	0	0	0	0	0.2	0	0	0	0	0	0	0	0	0.1	0	0
**HOTG60**	*Prevotella sp._Oral_Taxon*	0.1	0.1	0	0.3	0	0	0	0.1	0.3	0	0	0.3	0	0.2	0	0	0	0	0	0	0	0.02

	_G60																						
**GGT1482;HOT572**	*Prevotella veroralis*	0	4.1	0	0	0	0	0	0	0	0	0	0	0	0	0	0	0	0	0	0	0	0
**HOT494**	*Lachnoanaerobaculum saburreum*	0	0	0.2	0.1	0	0.1	0	0	0	0	0	1	1.6	0.1	0	0	0	0	0	0	0.1	0.04
**HOT286**	*Tannerella sp._Oral_Taxon_286*	0.1	0	0	0.1	0	0.1	0	0	0	0	0	0.8	0.6	0.1	0	0.2	0	0.1	0	0	0	0.03
**HOT300**	*Prevotella sp._Oral_Taxon_300*	0.8	0.4	0	0	0	0	0	0	0	0	0	0	0	0	0	0	0.1	0	0	0	0	0
**GGT675;HOT118**	*Dialister invisus*	1.7	1.2	0	0	0	0	0	0	0	0	0	0	0	0	0	0	0	0	0	0	0.1	0
**GGT1681;HOT130**	*Selenomonas noxia*	0	0	0.1	0.1	0	0	0	0	0.1	0	0.1	0.8	0.3	0.1	0	0	0	0	0	0	0.1	0
**GGT155;HOT723**	*Atopobium parvulum*	0.2	0	0.3	0	0.4	0	0	0.1	0.1	0	0	0.1	0.1	0	0	0	0	0	0	0.3	0	0.28
**GGT376;HOT575**	*Campylobacter concisus*	0	0	0.3	0.6	0.1	0.1	0	0.2	0.1	0	0	0.1	0.1	0.1	0	0	0	0.1	0	0.2	0.2	0.07
**GGT1031;HOT615**	*Lactobacillus gasseri*	0.1	0.2	0	0	0	0	0	0.2	0.4	0.7	0	0	0	0	0	0.1	0	0	0.6	1.7	0	0
**HOT431**	*Streptococcus sp._Oral_Taxon_431*	0	0	0.1	0.1	0.1	0.3	0.1	0.7	0.1	0.5	0.1	0.2	0	0	0	0	0	0	0	0.1	0	0.02
**GGT1078;HOT224**	*Leptotrichia hofstadii*	0	0	0	0.1	0	0.1	0	0	0	0	0	0.1	2.3	0	0	0	0	0	0	0	0	0.33
**GGT388;HOT325**	*Capnocytophaga granulosa*	0	0	0	0	0	0.1	0	0	0	0	0.1	0.7	1.2	0	0	0	0	0	0	0	0.1	0.02
**HOT329**	*Capnocytophaga leadbetteri*	0	0	0.1	0.1	0	0.3	0	0	0	0	0.1	0.3	0.7	0.1	0	0	0	0	0	0	0.1	0.01
**GGT157;HOT750**	*Atopobium rimae*	1.6	0.1	0	0	0	0	0	0	0	0	0.1	0.1	0	0	0	0	0	0	0	0	0	0.02
**GGT816;HOT626**	*Gemella haemolysans*	0.1	0.7	0	0	0	0.8	0	0	0	0	0	0	0	0	0	0	0	0	0	0	0.1	0.01
**HOT458**	*Aggregatibacter sp._Oral_Taxon_458*	0	0	0	0.2	0	0.8	0	0	0	0	0	0	0.2	0.3	0.1	0	0	0	0	0	0.8	0.05
**GGT1815;HOT073**	*Streptococcus australis*	0	0	0	0	0	0.1	0	0.2	0.1	0	0	0.1	0.2	0	0	0	0	0	0	0.1	0	0.03
**GGT1485;HOT311**	*Prevotella oris*	1.9	0.6	0	0	0	0	0	0	0	0	0	0	0	0	0	0	0	0	0	0	0	0

**GGT1627;HOT681**	*Rothia mucilaginosa*	0	0	0	0	0.2	0.7	0	0.2	0	0	0	0	0	0	0.2	0	0	0	0	0	0.1	0.05
**HOT313**	*Prevotella sp._Oral_Taxon_313*	0	0	0	0	0	0	0	0	0	0	0	0	0	0	0	0	0	0	0	0	0	0
**HOT322**	*Bergeyella sp._Oral_Taxon_322*	0	0	0	0.1	0	0.2	0	0	0	0	0	0.1	0.1	0.1	0.1	0	0	0.1	0	0	0.4	0.03
**HOT540**	*Cardiobacterium valvulum*	0	0	0	0	0	0	0	0	0	0	1.1	1.1	0.2	0.1	0	0	0	0	0	0	0	0.03
**GGT1473;HOT288**	*Prevotella oulorum*	0.3	0.3	0	0.1	0	0	0	0	0	0	0	0.3	0	0.1	0	0	0	0	0	0	0	0
**HOTB43**	*Porphyromonas sp._Oral_Taxon_B43*	0	0	0.1	0.2	0	0	0	0	0	0	0	0	0	0.1	0	0	0	0	0	0	1	0
**GGT392;HOT775**	*Capnocytophaga sputigena*	0.5	0	0.2	0.2	0	0.1	0	0	0	0	0.1	0.1	0.1	0	0	0	0	0	0	0	0.3	0.03
**HOT419**	*Moryella sp._Oral_Taxon_419*	0.1	0.2	0.1	0	0	0.1	0	0	0	0	0	0	0	0	0	0	0	0	0	0	0	0
**HOTF95**	*Capnocytophaga sp._Oral_Taxon_F95*	0	0	0	0	0	0	0	0	0	0	0.6	1.9	0	0	0	0	0	0	0	0	0	0.01
**GGT1486;HOT714**	*Prevotella pallens*	0	0	0	0	0	0	0	0.1	0	0	0	0	0	0	0	0	0	0	0	0	0	0.01
**HOT335**	*Capnocytophaga sp._Oral_Taxon_335*	0	0	0	0	0	0	0	0	0	0	0.4	1.9	0	0	0	0	0	0	0	0	0	0
**GGT2038**	*Yersinia mollaretii*	0	0	0	0	0	0	0	0	0	0	0	0	0.1	0	0	0	0	0	0	0	0	0
**GGT1788;HOT576**	*Streptococcus constellatus*	0.4	0.4	0	0	0	0	0	0.1	0	0	0	0	0	0	0	0	0	0	0	0	0	0
**HOT347**	*TM7_[G-1] sp._Oral_Taxon_347*	0	0	0	0	0	0	0	0	0	0	0	0	0	0	0	0.1	0	0	0	0	0.1	0.03
**GGT53;HOT701**	*Actinomyces odontolyticus*	0	0	0.2	0.1	0.2	0.1	0	0.2	0.1	0	0.1	0.1	0	0.1	0.1	0.1	0	0	0	0	0.1	0.08
**GGT1022;HOT817**	*Lactobacillus crispatus*	0	0	0	0	0	0	0	0	0	0	0.2	0	0	0	0	0	0	0	0	0	0	0
**HOT074**	*Streptococcus sp._Oral_Taxon_074*	0	0	0	0.1	0	0.2	0	0.1	0	0	0	0.1	0	0	0	0	0.1	0.1	0.1	0.2	0	0.02

**GGT706;HOT577**	*Eikenella corrodens*	0	0	0	0	0	0.1	0	0	0	0	0.1	0.2	0	0.1	0	0.1	0	0.1	0	0	0	0
**GGT711**	*Enterobacter cloacae*	0	0	0	0	0	0	0	0	0	0	0.1	0.1	0.1	0	0	0	0	0	0	0	0	0
**GGT1811;HOT721**	*Streptococcus parasanguinis_I*	0	0	0	0	0	0.1	0	0.6	0	0.1	0	0	0	0	0	0	0	0	0	0	0	0
**HOTH27**	*Fusobacterium sp._Oral_Taxon_H27*	0.2	0.2	0	0	0	0	0	0	0	0	0	0	0	0	0	0	0	0	0	0	0	0
**HOT888**	*Actinomyces dentalis*	0	0	0.1	0	0	0.1	0	0.1	0	0	0	0	0	0	0	0	0.1	0	0	0	0	0
**GGT1570;HOT854**	*Ralstonia pickettii*	0	0	0	0	0	0	0	0	0	0	0.1	0	0	0	0	0	0	0	0	0	0	0
**HOT351**	*TM7_[G-3] sp._Oral_Taxon_351*	0.6	0.4	0	0	0	0	0	0	0	0	0	0	0	0	0	0	0	0	0	0	0	0
**HOT097**	*Moryella sp._Oral_Taxon_097*	0.1	0.1	0.2	0	0	0.1	0	0	0	0	0	0	0	0	0	0	0	0	0	0	0	0.01
**HOT412**	*Capnocytophaga sp._Oral_Taxon_412*	0	0	0	0	0	0	0	0	0	0	0	0	0.9	0	0	0	0	0	0	0	0.3	0
**HOT326**	*Capnocytophaga sp._Oral_Taxon_326*	0	0	0.1	0.1	0	0	0	0	0	0	0	0.3	0	0	0	0	0	0	0	0	0	0
**GGT1074;HOT563**	*Leptotrichia buccalis*	0	0	0	0	0	0.1	0	0	0	0	0	0	0.3	0	0	0	0	0	0	0	0	0.01
**GGT1000;HOT582**	*Kingella denitrificans*	0.1	0	0	0	0	0	0	0	0	0	0	0	0.4	0.1	0	0	0	0	0	0	0	0.07
**HOT461**	*Lactobacillus sp._Oral_Taxon_461*	0.1	1.3	0	0	0	0	0	0	0	0	0	0	0	0	0	0	0	0	0	0	0	0
**HOTD12**	*Dietzia sp._Oral_Taxon_D12*	0	0	0	0	0	0	0	0	0	0	0	0	0	0	0	0	0	0	0	0	0	0
**GGT2084;HOT155**	*Veillonellaceae_[G-1] sp._Oral_Taxon_155*	0	0	0	0	0	0	0	0.1	0	0	0	0	0	0	0	0	0	0	0	0	0	0
**GGT1361;HOT807**	*Olsenella sp._Oral_Taxon_807*	0	0.1	0	0	0	0	0	0	0	0	0	0	0.5	0	0	0	0	0	0	0	0	0

**HOT131**	*Mitsuokella sp._Oral_Taxon_131*	0	0	0	0	0	0.1	0	0	0	0	0	0	0.1	0	0	0.1	0.1	0.2	0.1	0.1	0	0
**GGT1041;HOT051**	*Lactobacillus vaginalis*	0	0	0	0	0	0	0	0	0	0	0	0	0	0	0	0	0	0	1	0.3	0	0
**HOT336**	*Capnocytophaga sp._Oral_Taxon_336*	0	0	0.1	0.1	0	0.1	0	0	0	0	0	0	0.3	0	0	0	0	0.2	0	0	0	0
**GGT1362;HOT806**	*Olsenella profusa*	0	0	0.2	0	0.3	0	0	0	0.1	0	0	0	0	0	0	0.1	0	0	0	0	0	0
**GGT573;HOT579**	*Cryptobacterium curtum*	0.1	0.3	0	0	0	0	0	0	0	0	0	0	0.1	0	0	0	0	0	0	0	0	0.02
**GGT1989;HOT524**	*Veillonella atypica*	0	0	0	0	0	0	0	0	0	0	0	0	0	0	0	0	0	0	0	0.4	0	0.02
**HOT473**	*Alloprevotella sp._Oral_Taxon_473*	0	0.1	0	0	0	0.3	0	0	0	0	0	0	0	0	0	0	0	0	0	0	0	0
**GGT1818;HOT021**	*Streptococcus vestibularis*	0	0.2	0	0	0	0	0	0	0	0	0	0	0	0	0	0	0.6	0	0.1	0.2	0	0.01
**HOTE78**	*Streptococcus sp._Oral_Taxon_E78*	0	0	0	0	0	0	0.1	0	0	0.1	0.2	0.1	0	0	0	0	0	0	0	0	0.1	0
**GGT806;HOT201**	*Fusobacterium periodonticum*	0	0	0	0.1	0	0.2	0	0	0	0	0	0	0	0.1	0	0	0	0	0	0	0	0
**GGT1114;HOT122**	*Megasphaera micronuciformis*	0	0.1	0	0	0	0	0	0	0.1	0	0	0	0	0	0	0	0	0	0	0	0.1	0.03
**HOT739**	*Propionibacterium propionicum*	0	0	0	0	0	0	0	0	0	0	0	0	0	0	0	0.1	0.1	0	0	0	0.2	0.01
**GGT813;HOT757**	*Gemella sanguinis*	0	0	0	0.1	0	0.1	0	0.1	0	0	0	0	0	0	0	0	0	0	0	0	0	0.03
**HOT660**	*Defluvibacter lusatiensis*	0	0	0	0	0	0	0	0	0	0	0	0	0	0	0	0	0	0	0	0	0	0
**HOT057**	*Streptococcus sp._Oral_Taxon_057*	0	0	0	0	0	0	0	0.1	0	0	0	0.2	0	0	0	0	0	0	0.1	0	0	0
**GGT530;HOT595**	*Corynebacterium durum*	0	0	0	0	0	0.1	0	0	0	0	0	0	0	0	0	0	0	0	0	0	0	0.19
**HOT324**	*Capnocytophaga sp._Oral_Taxon_324*	0	0	0.2	0.1	0	0	0	0	0	0	0	0	0	0.1	0	0	0	0.1	0	0	0	0
**GGT1758**	*Staphylococcus*	0	0	0	0	0	0	0	0	0	0	0	0	0	0	0	0	0	0	0	0	0	0

	equorum																						
**GGT1367;HOT457**	*Oribacterium sinus*	0	0	0.1	0.1	0	0	0	0.1	0	0	0	0	0	0	0	0	0	0	0	0	0.1	0.01
**HOTA58**	*Acinetobacter sp._Oral_Taxon_A58*	0	0	0	0	0	0	0	0	0	0	0	0	0	0	0	0	0	0	0	0	0	0
**GGT1608**	*Riemerella anatipestifer*	0	0	0	0	0	0	0	0	0	0	0	0	0	0	0	0	0	0	0	0	0	0.31
**HOT870**	*TM7_[G-1] sp._Oral_Taxon_870*	0	0	0	0	0	0	0	0	0	0	0	0	0	0	0	0	0	0	0	0	0	0
**HOT278**	*Porphyromonas sp._Oral_Taxon_278*	0	0	0	0	0	0.7	0	0	0	0	0	0	0	0	0	0	0	0	0	0	0	0
**HOTA88**	*Pseudomonas mosselii_Oral_Taxon_A88*	0	0	0	0	0	0	0	0	0	0	0.5	0	0	0	0	0	0	0	0	0	0	0
**GGT1304;HOT598**	*Neisseria elongata*	0	0	0	0	0	0.1	0	0	0	0	0	0	0.2	0.1	0	0	0	0	0	0	0	0.02
**HOT078**	*Oribacterium sp._Oral_Taxon_078*	0.5	0.1	0	0	0	0	0	0	0	0	0	0	0	0	0	0	0	0	0	0.1	0	0
**HOT352**	*TM7_[G-1] sp._Oral_Taxon_352*	0	0	0	0	0	0	0	0.1	0	0	0	0	0	0	0	0	0	0	0	0	0	0.03
**GGT1488;HOT299**	*Prevotella sp._Oral_Taxon_299*	0	0	0.1	0.1	0.1	0.1	0	0	0	0	0	0	0	0	0	0	0	0	0	0	0	0.01
**HOT308**	*Alloprevotella sp._Oral_Taxon_308*	0	0	0	0	0	0.1	0	0	0	0	0	0.1	0	0	0	0	0	0	0	0	0.1	0.01
**GGT1536;HOT612**	*Pseudomonas fluorescens*	0	0	0	0	0	0	0	0	0	0	0	0	0.1	0	0	0	0	0	0.2	0	0	0
**GGT1820**	*Streptococcus sp._str._M334*	0	0	0	0	0	0	0.3	0	0.1	0.1	0	0	0	0	0	0	0.1	0	0	0	0	0
**HOT096**	*Lachnospiraceae_[G-2] sp._Oral_Taxon_096*	0	0	0.5	0.1	0	0	0	0	0	0	0	0	0	0	0	0	0	0	0	0	0	0
**GGT1490;HOT289**	*Prevotella maculosa*	0.4	0	0	0	0	0	0	0	0	0	0	0	0	0	0	0	0	0	0	0	0	0
**GGT1498;HOT466**	*Alloprevotella tannerae*	0	0	0	0	0	0	0	0	0	0	0	0	0	0	0	0	0	0	0	0	0	0

**GGT1043;HOT756**	*Lactobacillus salivarius*	0	0	0	0	0	0	0	0	0	0	0	0	0	0	0	0	0	0	0.3	0.2	0	0
**GGT33**	*Acinetobacter sp._str._DR1*	0	0	0	0	0	0	0	0	0	0	0	0	0	0	0	0	0	0	0	0	0	0.01
**GGT1077;HOT214**	*Leptotrichia shahii*	0	0	0	0	0	0	0	0	0	0	0	0	0	0	0	0	0	0	0	0	0	0.01
**GGT380;HOT763**	*Campylobacter showae*	0	0	0	0	0	0	0	0	0	0	0	0.1	0.1	0	0	0	0	0	0	0	0	0
**HOT107**	*Lachnoanaerobaculum umeaense*	0.1	0.1	0.1	0	0	0	0	0	0	0	0	0.1	0	0	0	0	0	0	0	0	0	0
**HOT221**	*Leptotrichia sp._Oral_Taxon_221*	0	0	0.1	0.2	0	0	0	0	0	0	0	0	0	0.1	0	0	0	0	0	0.1	0.1	0
**HOT349**	*TM7_[G-1] sp._Oral_Taxon_349*	0	0	0	0	0	0	0	0	0	0	0	0	0	0	0	0	0	0	0	0	0	0
**HOTE20**	*Selenomonas sp._Oral_Taxon_E20*	0	0	0	0	0	0	0	0	0	0	0	0	0	0	0	0	0	0	0	0	0	0
**HOT100**	*Lachnospiraceae_[G-3] sp._Oral_Taxon_100*	0	0	0	0	0	0	0	0	0	0	0	0	0.1	0	0	0	0	0	0	0	0	0
**HOT887**	*Veillonella denticariosi*	0	0	0	0	0	0	0	0	0	0	0	0	0	0	0	0	0	0	0	0	0	0
**HOT215**	*Leptotrichia sp._Oral_Taxon_215*	0	0	0.1	0	0	0.1	0	0	0	0	0	0	0	0	0	0	0	0	0	0	0	0
**GGT1034**	*Lactobacillus helveticus*	0	0	0	0	0	0	0	0	0	0	0.1	0	0	0	0	0	0	0	0	0	0	0.01
**HOT900**	*Bergeyella sp._Oral_Taxon_900*	0	0	0	0	0	0	0	0	0	0	0	0	0.2	0	0	0	0	0	0	0	0	0.02
**GGT803;HOT200**	*Fusobacterium nucleatum_ss_vincentii*	0	0	0	0	0	0	0	0	0	0	0	0	0	0.3	0	0	0	0	0	0	0	0.02
**HOT323**	*Capnocytophaga sp._Oral_Taxon_323*	0	0	0	0	0	0	0	0	0	0	0	0	0	0	0	0	0	0	0	0	0	0
**HOTE63**	*Actinomyces sp._Oral_Taxon_E63*	0	0	0	0	0	0.2	0	0	0	0	0	0	0	0	0	0	0	0	0	0	0	0

**HOT913**	*Alloprevotella sp._Oral_Taxon_913*	0	0	0	0	0	0	0	0	0	0	0	0	0	0	0	0	0	0	0	0	0	0
**HOT886**	*Streptococcus oligofermentans*	0	0	0	0	0	0	0	0	0	0	0	0	0.4	0	0	0	0	0	0	0	0	0
**HOT423**	*Streptococcus sp._Oral_Taxon_423*	0	0	0	0	0	0	0	0	0	0	0	0	0	0	0	0.1	0	0	0	0	0	0
**GGT1813**	*Streptococcus sp._Oral_Taxon_71*	0	0	0	0	0	0	0	0	0	0	0	0	0	0	0.1	0	0	0	0	0	0	0
**HOT181**	*Actinomyces sp._Oral_Taxon_181*	0	0	0	0	0	0.1	0	0.1	0	0	0	0	0	0	0	0	0	0	0	0	0	0
**HOTE28**	*Streptococcus sp._Oral_Taxon_E28*	0	0	0	0	0	0	0	0	0	0	0	0	0	0	0	0	0	0	0	0	0	0
**HOTH23**	*Selenomonas sp._Oral_Taxon_H23*	0	0	0	0	0	0	0	0	0	0	0	0	0	0	0	0	0	0	0	0	0	0
**HOT769**	*Treponema socranskii_ss_socranskii*	0.2	0.1	0	0	0	0	0	0	0	0	0	0	0	0	0	0	0	0	0	0	0	0
**HOT874**	*SR1_[G-1] sp._Oral_Taxon_874*	0	0	0	0	0	0	0	0	0	0	0	0	0	0	0	0	0	0	0	0	0	0
**GGT387;HOT700**	*Capnocytophaga ochracea*	0	0	0	0	0	0	0	0	0	0	0.1	0.3	0	0.1	0	0	0	0	0	0	0	0
**GGT1676;HOT151**	*Selenomonas sputigena*	0.1	0	0	0	0	0	0	0	0	0	0	0	0	0	0	0	0	0	0	0	0	0
**GGT1479;HOT794**	*Prevotella multisaccharivorax*	0	0	0	0	0	0	0	0	0	0	0	0	0	0	0	0	0	0	0	0	0	0
**GGT1821;HOT728**	*Streptococcus peroris*	0	0	0	0	0	0.1	0	0	0	0	0	0	0	0	0	0	0	0	0	0	0	0
**GGT1432;HOT111**	*Parvimonas micra*	0.3	0	0	0	0	0	0	0	0	0	0	0	0	0	0	0	0	0	0	0	0	0
**GGT1302;HOT729**	*Neisseria pharyngis*	0	0	0	0	0	0	0	0	0	0	0	0	0	0	0	0	0	0	0	0	0	0.06
**HOT487**	*Streptococcus sp._Oral_Taxon_487*	0	0	0	0	0	0.1	0	0	0	0	0	0	0	0	0	0	0	0	0	0	0	0.02
**HOT808**	*Tannerella*	0.1	0	0	0	0	0	0	0	0	0	0	0	0	0	0	0	0	0	0	0	0	0

	sp._Oral_Taxon _808																						
**HOTA16**	*Corynebacterium sp._Oral_Taxon _A16*	0	0	0	0	0	0	0.1	0.1	0	0	0	0	0	0	0	0	0	0	0	0	0	0.01
**HOT565**	*Enterobacter cancerogenus*	0	0	0	0	0	0	0	0	0	0	0.1	0	0	0	0	0	0	0	0	0	0	0
**GGT1037;HOT818**	*Lactobacillus reuteri*	0	0	0	0	0	0	0	0	0	0	0	0	0	0	0	0	0	0	0	0	0	0
**GGT306**	*Brevibacillus brevis*	0	0	0	0	0	0	0	0	0	0	0.1	0	0	0	0	0	0	0	0	0	0	0
**HOT914**	*Alloprevotella sp._Oral_Taxon _914*	0	0	0	0	0	0.1	0	0	0	0	0	0	0	0	0	0	0	0	0	0	0	0.01
**HOT225**	*Leptotrichia sp._Oral_Taxon _225*	0	0	0	0	0	0	0	0	0	0.1	0	0	0	0	0	0	0	0	0	0	0	0
**HOT203**	*Fusobacterium sp._Oral_Taxon _203*	0	0.1	0	0	0	0	0	0	0	0	0	0	0	0	0	0	0	0	0	0	0	0.02
**HOTG67**	*Selenomonas sp._Oral_Taxon _G67*	0	0	0	0	0	0	0	0	0	0	0	0.2	0	0	0	0	0	0	0	0	0	0
**GGT10;HOT343**	*Achromobacter xylosoxidans*	0	0	0	0	0	0	0	0	0	0	0	0	0	0	0	0	0	0	0	0	0	0
**HOTF11**	*Streptococcus sp._Oral_Taxon _F11*	0	0	0	0	0	0	0	0	0	0	0	0	0	0	0	0	0	0	0	0	0	0.04
**GGT1026;HOT749**	*Lactobacillus rhamnosus*	0	0	0	0	0	0	0	0	0	0	0	0	0	0	0	0	0	0	0	0	0	0.03
**GGT76;HOT762**	*Aggregatibacter segnis*	0	0	0	0	0	0	0	0	0	0	0	0	0	0.1	0	0	0	0	0	0	0	0
**GGT863;HOT851**	*Haemophilus haemolyticus*	0	0	0	0	0	0.1	0	0	0	0	0	0	0	0	0	0	0	0	0	0	0	0
**HOT912**	*Alloprevotella sp._Oral_Taxon _912*	0	0	0	0	0	0	0	0	0	0	0	0	0	0	0	0	0	0	0	0	0	0
**HOT557**	*Eubacterium_[XI][G-3] brachy*	0.2	0.1	0	0	0	0	0	0	0	0	0	0	0	0	0	0	0	0	0	0	0	0
**HOTC21**	*Kingella sp._Oral_Taxon _C21*	0	0	0	0	0	0	0	0	0	0	0	0	0	0	0	0	0	0	0	0	0.1	0
**GGT2;HOT1**	*Catonella morbi*	0.1	0	0	0	0	0	0	0	0	0	0	0	0	0	0	0	0	0	0	0	0	0

**65**																							
**HOT306**	*Prevotella sp._Oral_Taxon _306*	0	0	0	0	0	0	0	0	0.1	0	0	0	0	0	0	0	0	0	0	0	0	0.01
**GGT1020;HOT568**	*Lactobacillus casei*	0	0	0	0	0	0	0	0	0	0	0	0	0	0	0.1	0	0	0	0	0	0	0
**HOTE72**	*Streptococcus sp._Oral_Taxon _E72*	0	0	0	0	0	0	0	0	0	0	0	0	0	0	0	0	0	0	0	0	0	0.01
**HOT075**	*Clostridiales_[F-2][G-1] sp._Oral_Taxon _075*	0.1	0	0	0.1	0	0	0	0	0	0	0	0	0	0	0	0	0	0	0	0	0	0
**GGT1534;HOT536**	*Pseudomonas aeruginosa*	0	0	0	0	0	0	0	0	0	0	0	0	0	0	0	0	0	0	0	0	0	0
**HOTA56**	*TM7_[G] sp._Oral_Taxon _A56*	0	0	0	0	0	0	0	0.1	0	0	0	0	0	0	0	0	0	0	0	0	0	0.01
**HOT124**	*Selenomonas artemidis*	0	0	0	0	0	0	0	0	0	0	0	0	0	0	0	0	0	0	0	0	0	0
**HOT865**	*Kluyvera ascorbata*	0	0	0	0	0	0	0	0	0	0	0	0	0	0	0	0	0	0	0	0	0	0
**HOT305**	*Prevotella sp._Oral_Taxon _305*	0	0	0	0	0	0	0	0	0.2	0	0	0	0	0	0	0	0	0	0	0	0	0
**HOT353**	*TM7_[G-1] sp._Oral_Taxon _353*	0	0	0	0	0	0	0	0	0	0	0	0	0	0	0	0	0	0	0	0	0	0
**HOTF93**	*Lachnospiraceae_[G] sp._Oral_Taxon _F93*	0	0	0	0	0	0	0	0	0	0	0	0.2	0	0	0	0	0	0	0	0	0	0
**GGT78;HOT485**	*Agrobacterium tumefaciens*	0	0	0	0	0	0	0	0	0	0	0	0	0	0	0	0	0	0	0	0	0	0
**HOT303**	*Prevotella pleuritidis*	0	0	0	0	0	0	0	0	0	0	0	0	0	0	0	0	0	0	0	0	0	0
**HOT121**	*Anaeroglobus geminatus*	0	0	0	0	0	0	0	0	0	0	0	0	0	0	0	0	0	0	0	0	0	0
**HOT212**	*Leptotrichia sp._Oral_Taxon _212*	0	0	0	0	0	0	0	0	0	0	0	0.1	0	0	0	0	0	0	0	0	0	0
**HOT082**	*Lachnoanaerobaculum orale*	0	0	0.1	0	0	0	0	0	0	0	0	0	0	0	0	0	0	0	0	0	0	0

**HOTA46**	*Corynebacterium sp._Oral_Taxon_A46*	0	0	0	0	0	0.2	0	0	0	0	0	0	0	0	0	0	0	0	0	0	0	0
**GGT1723;HOT678**	*Solobacterium moorei*	0	0	0	0	0	0	0	0	0	0	0	0	0	0	0	0	0	0	0	0	0	0
**HOTC60**	*Manihot esculenta_Oral_Taxon_C60*	0	0	0	0	0	0	0	0	0	0	0	0	0	0	0	0	0	0	0	0	0	0
**HOTG62**	*Streptococcus sp._Oral_Taxon_G62*	0	0	0	0	0	0	0	0	0	0	0	0	0	0	0	0	0	0	0	0	0	0
**GGT1478;HOT693**	*Prevotella nigrescens*	0.1	0.1	0	0	0	0	0	0	0	0	0	0	0	0	0	0	0	0	0	0	0	0
**HOT085**	*Clostridiales_[F-2][G-2] sp._Oral_Taxon_085*	0	0.1	0	0	0	0	0	0	0	0	0	0	0	0	0	0	0	0	0	0	0	0
**GGT1680;HOT149**	*Selenomonas sp._Oral_Taxon_149*	0	0	0	0	0	0	0	0	0	0	0	0	0	0	0	0	0	0	0	0	0	0
**HOTG30**	*Veillonella sp._Oral_Taxon_G30*	0	0	0	0	0	0	0	0	0	0	0	0	0	0	0	0	0	0	0	0	0	0
**GGT673;HOT736**	*Dialister pneumosintes*	0	0	0	0	0	0	0	0	0	0	0	0	0	0	0	0	0	0	0	0	0	0
**HOT414**	*Actinomyces sp._Oral_Taxon_414*	0	0	0	0.1	0	0	0	0	0	0	0	0	0	0	0	0	0	0	0	0	0	0
**HOT781**	*Prevotella saccharolytica*	0	0	0	0	0	0	0	0	0	0	0	0	0	0	0	0	0	0	0	0	0	0
**HOT671**	*Actinomyces meyeri*	0	0.1	0	0	0	0	0	0	0	0	0	0	0	0	0	0	0	0	0	0	0	0
**GGT724;HOT803**	*Enterococcus italicus*	0	0	0	0	0	0	0	0	0	0	0	0	0	0	0	0	0	0	0	0	0	0.16
**GGT1497;HOT665**	*Prevotella marshii*	0.1	0	0	0	0	0	0	0	0	0	0	0	0	0	0	0	0	0	0	0	0	0
**GGT61;HOT178**	*Actinomyces sp._Oral_Taxon_178*	0	0	0	0	0	0	0	0	0	0	0	0	0.1	0	0	0	0	0	0	0	0	0
**GGT2076;HOT274**	*Bacteroidales_[G-2] sp._Oral_Taxon_274*	0	0.1	0	0	0	0	0	0	0	0	0	0	0	0	0	0	0	0	0	0	0	0

**HOTE05**	*Streptococcus sp._Oral_Taxon _E05*	0	0	0	0	0	0	0	0	0	0	0	0	0	0	0	0	0	0	0	0	0	0
**HOT223**	*Leptotrichia sp._Oral_Taxon _223*	0.1	0	0	0	0	0	0	0	0	0	0	0	0	0	0	0	0	0	0	0	0	0
**HOTE83**	*Selenomonas sp._Oral_Taxon _E83*	0	0	0	0	0	0	0	0	0	0	0	0	0	0	0	0	0	0	0	0	0	0
**HOT069**	*Streptococcus sp._Oral_Taxon _069*	0	0	0	0	0	0	0	0	0	0	0	0	0	0	0	0	0	0	0	0	0	0
**GGT1792**	*Streptococcus thermophilus*	0	0	0	0	0	0	0	0	0	0	0	0	0	0	0	0	0	0	0	0	0	0.02
**GGT765**	*Faecalibacteriu m prausnitzii*	0	0	0	0	0	0	0	0	0	0	0	0	0	0	0	0	0	0	0	0	0	0
**HOT613**	*Tannerella forsythia*	0	0	0	0	0	0	0	0	0	0	0	0	0	0	0	0	0	0	0	0	0	0
**HOT652**	*Afipia sp._genomospe cies_4*	0	0	0	0	0	0	0	0	0	0	0	0	0	0	0	0	0	0	0	0	0	0
**HOT108**	*Oribacterium sp._Oral_Taxon _108*	0	0	0	0	0	0	0	0	0	0	0	0	0	0	0	0	0	0	0	0	0	0
**HOT350**	*TM7_[G-2] sp._Oral_Taxon _350*	0	0	0	0	0	0	0	0	0	0	0	0	0	0	0	0	0	0	0	0	0	0
**HOTD95**	*Fusobacterium sp._Oral_Taxon _D95*	0.1	0	0	0	0	0	0	0	0	0	0	0	0	0	0	0	0	0	0	0	0	0
**GGT1481**	*Prevotella copri*	0	0	0	0	0	0	0	0	0	0	0	0	0	0	0	0	0	0	0	0	0	0
**GGT1352;H OT544**	*Ochrobactrum anthropi*	0	0	0	0	0	0	0	0	0	0	0	0	0	0	0	0	0	0	0	0	0	0
**GGT1678;H OT125**	*Selenomonas flueggei*	0	0	0	0	0	0	0	0	0	0	0	0	0	0	0	0	0	0	0	0	0	0
**GGT710**	*Enterobacter sp._str._HCB*	0	0	0	0	0	0	0	0	0	0	0	0	0	0	0	0	0	0	0	0	0	0
**HOTH38**	*Cardiobacteriu m sp._Oral_Taxon _H38*	0	0	0	0	0	0	0	0	0	0	0	0	0.1	0	0	0	0	0	0	0	0	0
**GGT200**	*Bacteroides fragilis*	0	0	0	0	0	0	0	0	0	0	0	0	0	0	0	0	0	0	0	0	0	0

**GGT256;HOT588**	*Bifidobacterium dentium*	0	0	0	0	0	0	0	0	0	0	0	0	0	0	0	0	0	0	0	0	0	0
**HOTE49**	*Aggregatibacter sp._Oral_Taxon_E49*	0	0	0	0	0	0	0	0	0	0	0	0.1	0	0	0	0	0	0	0	0	0	0
**HOT213**	*Leptotrichia hongkongensis*	0	0	0	0	0	0	0	0	0	0	0	0	0	0	0	0	0	0	0	0	0	0
**GGT918**	*Halomonas sp._str._HTNK1*	0	0	0	0	0	0	0	0	0	0	0	0	0	0	0	0	0	0	0	0	0	0
**HOT866**	*Actinomyces graevenitzii*	0	0	0	0	0	0	0	0	0	0	0	0	0	0	0	0	0	0	0	0	0	0
**HOT600**	*Prevotella enoeca*	0	0	0	0	0	0	0	0	0	0	0	0	0	0	0	0	0	0	0	0	0	0
	**Column Sum**	**99.9**	**99.9**	**99.5**	**99.9**	**98.8**	**99.5**	**99.8**	**99.8**	**99.4**	**99.9**	**99.9**	**99.8**	**99.9**	**99.8**	**99.8**	**100**	**99.8**	**99.9**	**99.9**	**99.8**	**99.9**	**99.86**

**Relative abundance ≧0.1**		**Diseased (AWSL) occlusal surfaces (bacterial microbiome from each surface sampled)**																						
**Taxon_ID**	**Species**	**137**	**147**	**217**	**227**	**247**	**327**	**417**	**427**	**437**	**447**	**617**	**627**	**737**	**817**	**827**	**917**	**927**	**937**	**1017**	**1027**	**1117**	**1127**	**1137**	**1237**
**GGT1;HOT534**	*Granulicatella paradiacens*	5.2	9.2	3.5	19.3	3.1	16.6	29.7	11.1	47.7	30.5	4.5	9.8	5.7	23	27.6	20.6	9.3	29.6	0.9	5.8	11.9	10.6	2.5	5.5
**GGT1799;HOT686**	*Streptococcus mutans*	6.9	26.2	0.6	1	5.8	0.3	3.9	19.8	5.3	11	1.5	0.4	0.3	2.8	2.8	5.7	26.1	10.8	0.9	0.8	3.6	13.3	2.4	3.7
**GGT1822**	*Streptococcus sp._str._C300*	2.4	4.1	7.4	17	14.9	19.6	7.3	4.9	4.2	3.3	5.3	5.1	0.7	4.2	11.1	6	5.5	2.8	0.4	1.1	3.3	3.6	0.2	9.1
**GGT1051;HOT022**	*Lautropia mirabilis*	2.8	2.2	30.4	12.7	21.9	6	2.8	1.8	2.7	2.5	5.8	9.5	4.1	3.3	0.6	5	0.5	2.5	0.2	0	4.1	6.1	0.8	0.1
**HOTC61**	*Pseudomonas sp._Oral_Taxon_C61*	0	0	0	0	0	0	0	0	0	0	0	0	39.1	0	0	0	0	0	39.3	0	0	0	0	23.7
**GGT1803;HOT622**	*Streptococcus gordonii*	1.7	5.5	0.6	1.5	0.4	6.7	8.7	7.2	4	5.4	1.1	2.2	0.1	3.3	4.1	4.8	11.4	8.6	0	0.1	3.9	2.6	0.2	9
**GGT1789;HOT758**	*Streptococcus sanguinis*	0.7	0.3	5.8	2.6	4.6	3.3	6	4	2.6	3.3	9.7	4.5	1.7	3.2	3.2	1.9	5.8	2.6	0.7	0.4	1.2	2.1	0.5	0.2
**HOT389**	*Abiotrophia defectiva*	0.8	0.4	0.9	4.3	0.3	4.8	1.2	1.7	2	4.7	0	0	0	0.3	0.9	0.2	0.4	0.5	0	0.1	11.5	15.1	5.8	0.1
**GGT1806;HOT707**	*Streptococcus oralis*	0	0	0.1	0.6	0.1	0.1	7.5	3.7	0.5	7.7	16.8	14.1	0	0	0.1	3.1	4.2	1.7	0	0	10.8	0.3	0	0

**HOTE53**	*Veillonella sp._Oral_Taxon_E53*	1.4	4.1	7.1	1.6	8.8	3.1	1.2	1.9	2.7	1	0.6	4.8	3.7	1.6	0.1	2	1.5	1.7	1.7	14.2	6.6	2.6	0.4	0.3
**GGT1533**	*Pseudomonas putida*	0	0	0	0	0	0	0	0	0	0	36.2	0	0	0	0	0	0	0	0	0	0	0	0	0
**GGT1312;HOT764**	*Neisseria sicca*	0.1	0.1	0.6	4.1	0	0.1	0	0	0	0	0	0	2	17.2	35	0	0	0	0	0	0	0	0	0.1
**GGT1991;HOT158;HOT160;HOT161**	*Veillonella parvula_group*	0.7	2.4	4.6	1.6	6.5	1	0.6	0.8	1.1	0.5	0.7	5.5	1.7	0.7	0.1	0.9	0.4	0.3	2.6	15.5	2.8	1.3	0.1	0.1
**GGT738**	*Escherichia coli*	0	0	0	0	0	0	0	0	0	0	0.1	0.1	0	0	0	0	0	0	0	0	0	0	85	0.3
**GGT32; HOT554**	*Acinetobacter baumannii*	0	0	0	0	0	0	1.2	0	0	0	0	0	0	0	0	0	0	0	0	0	0	0	0	0
**HOT064**	*Streptococcus sp._Oral_Taxon_064*	0	0.1	0.1	0.1	0.1	0.2	14.7	9.6	4.4	13.8	1	1.5	0	3.9	3.1	0.2	0.1	0	0	0	0.1	0	0	0.6
**GGT1784;HOT734**	*Streptococcus pneumoniae*	0	0.3	0.1	0.5	0.1	0.5	0.4	0.8	0.2	0.7	0.1	0.3	0.1	0.2	0.7	22.1	1.9	7.9	0.9	0.1	0.3	0.1	0	0.1
**GGT1484;HOT291**	*Prevotella denticola*	6.5	5.6	0	0	0	0	0	0.5	0.9	0	0	0	0.2	0	0	0	2	0.3	0	0	1.4	0	0	5.1
**GGT712**	*Enterobacter sp._str._638*	0	0	0	0	0	0	0	0	0	0	0	0.6	4.9	0	0	0	0	0	0	0	0	0	0	8
**GGT62; HOT171**	*Actinomyces sp._Oral_Taxon_171*	0	0	4.4	0.2	11	0.2	0.7	2.3	1.9	1	0.2	0.2	0.1	0	0	0.2	0.1	0.1	0.9	8.4	1.1	0.4	0.2	0.1
**GGT1809**	*Streptococcus sp._str._2136FAA*	0.1	0.1	0	0	0	0	0	0	0	0	0	0	0	0	0	2.1	18.7	9.3	2	3.3	0.1	0	0	0
**GGT1791;HOT578**	*Streptococcus cristatus*	0	0.1	1.1	5.5	0	5.7	0.4	0.8	0.6	0.6	0.5	1	2.1	1.7	0.8	0.9	2	1.3	0.1	0.1	4	2.8	0	0.3
**GGT1787;HOT398;HOT677**	*Streptococcus mitis*	0	0.4	0.4	0.4	0.1	2	0.7	1.2	0.5	0.3	0.8	4.2	0.6	2.4	1.2	0.3	1.1	1.3	0.3	0.3	3.7	0.8	0.1	2.3
**GGT1795;HOT755**	*Streptococcus salivarius*	0	0	0.1	0.1	0.1	0.4	2.7	4.8	0.5	2	0	0.1	0.1	0	0.1	0	0.4	0	1.3	3.4	0.1	0.4	0.1	1.4

**HOT071**	*Streptococcus sp._Oral_Taxon_071*	1.1	1.3	0	0.9	0	0.7	0.2	0.2	0.3	0.4	0.1	2.4	0.3	0	0	3.2	0.2	0.1	1.1	1.7	0.7	0.5	0	2
**GGT1819;HOT411**	*Streptococcus parasanguinis_II*	0	0	0	0	0.1	0.6	0.6	2.6	0.3	0.7	0	0.3	0.1	0.2	0.7	0	0.8	0.3	2.5	6.1	0.1	0.1	0	0.3
**HOT170**	*Actinomyces sp._Oral_Taxon_170*	0	0.1	9.8	1.9	4.1	0.3	0.2	0.2	0.2	0.1	2.5	4.6	0	0	0	0	0.1	0	0	0	1.4	0.6	0.3	0
**HOT768**	*Streptococcus sobrinus*	0	0	0	0.3	2.1	0	0	0	0	0	0	0	0	0	0	0	0	0	4.7	17.5	0	0	0	1.1
**GGT1483;HOT469**	*Prevotella melaninogenica*	8.1	6.4	0.2	0.7	0	1.8	0	0.6	2.2	0	0	0.9	0.1	0.1	0.1	0.1	0	0	0	0.1	0.1	0.2	0	0.1
**GGT1300;HOT682**	*Neisseria mucosa*	0	0.1	0.1	0.4	0	0.5	0	0.1	0	0	0	0	0.1	0.1	0.2	3.1	0.2	2	0	0	4.6	6.8	0	0.1
**HOT448**	*Actinomyces sp._Oral_Taxon_448*	0	0.2	0.2	0	0.2	0.2	2.1	3	1.9	3.5	0	0.5	0.4	0.1	0	0.8	0.9	5.6	0.4	1.2	1.3	0.8	0	0
**HOT169**	*Actinomyces sp._Oral_Taxon_169*	0.1	0.8	0.9	0.3	0.5	0.2	0.4	0	0	0	0	0	0	0.2	0.4	1.9	0.2	0.3	0.2	0.4	0.4	0.7	0.1	0.1
**GGT1025;HOT716**	*Lactobacillus paracasei*	0.1	0	0.2	0	0	0	0	0	0	0	0	0	0	0	0.1	0	0	0	0	0	0	0	0	0
**GGT1988;HOT717**	*Variovorax paradoxus*	26	0	0	0	0	0	0	0	0	0	0	0	0	0	0	0	0	0	0	0	0	0	0	0
**HOT070**	*Streptococcus sp._Oral_Taxon_070*	0	0.8	0	0	0	0.7	0.1	0.3	0.1	0.1	0	0	0	0	0.2	0	0	0	0	0	0	0	0	0.4
**HOT058**	*Streptococcus sp._Oral_Taxon_058*	0	0	0.4	0.9	0.1	1.6	0.3	0.1	0	0	0	0	1.1	0.5	0.2	0.2	1	0.2	0.1	0	0.3	2.3	0	0.4
**GGT378;HOT623**	*Campylobacter gracilis*	1.3	1.3	0.1	0.2	0	0.1	0	0.7	0.6	0.1	0.5	2.4	0.7	0.5	0	1	0.4	0.9	0.5	3	0.2	0.2	0	0.1
**GGT866;HOT718;HOT826**	*Haemophilus parainfluenzae*	0.3	0.4	4.4	1.5	3.2	1.2	0	0.2	0	0	0.1	0.4	0.3	0.4	0.3	0.1	0.1	0.1	0.2	0.2	0.5	0.5	0	0
**GGT1308;HOT4**	*Neisseria flavescens|su*	0	0.1	0.2	2.5	0.1	0.1	0	0.1	0.1	0	0	0.1	1.4	6.6	1.7	0.1	0	0	0	0	0.3	0.2	0	0

**76;HOT 610**	*bflava*																								
**GGT1546**	*Pseudomonas antarctica*	0	0	0	0	0	0	0	0	0	0	0	0	0	0	0	0	0	0	19.8	0	0	0	0	0
**GGT807;HOT420**	*Fusobacterium nucleatum_ss_animalis*	12.4	2.4	0	0.1	0	0.1	0	0.2	0	0	0	0	0	0	0	0.1	0	0	0.1	0	0.1	0	0	0.1
**HOT191**	*Propionibacterium acidifaciens*	0	0	0.2	0	0.3	0	0.2	2.7	2.6	0.9	0	0	0.1	0	0	3.1	0.5	2.1	0.1	0.2	0	0.1	0	0
**HOT065**	*Streptococcus sp._Oral_Taxon_065*	0	0	0.4	0.3	0.4	0.8	0	1	0.3	0.1	0.1	0.3	0.1	0.1	0.8	0	0	0	0	0.3	0.1	0.1	0	0
**HOT279**	*Porphyromonas sp._Oral_Taxon_279*	0	0	0.1	0.3	0	1.5	0	0	0	0	0.1	0.1	3	5.8	0.2	0	0.1	0.1	0	0	1.3	1.2	0	0
**GGT550;HOT666**	*Corynebacterium matruchotii*	0	0	2	1.2	0.1	0.7	0.8	0.8	0.5	0.2	0	0.1	2.1	1	0.1	0.4	0.1	0.2	0	0	1.2	1.1	0	0
**GGT60;HOT180**	*Actinomyces sp._Oral_Taxon_180*	0.4	0.4	2.1	1.1	1.1	0.7	0	0.1	0	0	0.1	0.1	0.1	0.1	0.1	0	0	0	0	0	0.1	0.1	0	0.1
**HOT195**	*Scardovia wiggsiae*	0.3	0.2	0.2	0	0.3	0	0.2	0.3	1.3	0.5	0.1	0.6	0	0	0	0.1	0.1	0.6	0.7	6.7	0	0	0	0.2
**GGT1075**	*Leptotrichia sp._AF189244.1*	0	0.1	0.6	0.6	0	0.2	0.4	0.5	0.2	0.3	0.1	0.5	0.3	0.3	0.1	0.1	0	0.1	0	0.1	2.4	3.7	0.1	0
**GGT731**	*Erwinia aphidicola*	0	0	0	0	0	0	0	0	0	0	0	0	0	0	0	0	0	0	0	0	0	0	0	14.2
**HOTB66**	*Streptococcus sp._Oral_Taxon_B66*	0	0	0.7	0.8	0.4	0	0	0.1	0.1	0	0	0.1	0.2	0	0	0	0	0	0	0	0.5	1.4	0.3	0
**HOT298**	*Prevotella histicola*	1.9	2.7	0.1	0.3	0.1	0	0.5	0.1	0.5	0.1	0	0.2	0	0.1	0.1	0.2	0	0.1	0	0.5	0.3	0.6	0	0
**HOT417**	*Leptotrichia sp._Oral_Taxon_417*	0	0	0.2	1.3	0	1	0	0.1	0	0	0	0.4	2.9	0.3	0	0	0	0.1	0	0	0.6	0.9	0	0
**GGT56**	*Actinomyces viscosus*	0	0.4	0.3	0.2	0.1	0	0.6	0.6	0.2	0.3	0	0	0	0	0	1	0.1	0.3	0	0	0.3	0.6	0	0
**GGT391;HOT337**	*Capnocytophaga gingivalis*	0	0	0.6	0.2	0	0.2	0	0	0	0	0.3	1.3	2.3	0.3	0	0	0	0.1	0	0	0.4	0.4	0	0

**GGT1023**	*Lactobacillus delbrueckii*	0	0	0	0	0	0	0	0	0	0	0	0	0	0	0	0	0	0	11.2	0.2	0	0	0	0
**HOT222**	*Leptotrichia wadei*	0	0.4	0.1	1.4	0	0.5	0	0.2	0.1	0.1	0	0	0.1	0.1	0	0.1	0.1	0.3	0	0.1	0.4	0.5	0	0
**HOT183**	*Actinobaculum sp._Oral_Taxon_183*	0	0	0	0	0	0	0.3	0.6	2.3	0.1	2.3	0.2	0.2	0	0	3.2	1	0.7	0	0.1	0	0	0	0
**GGT815;HOT046**	*Gemella morbillorum*	0.2	0.1	0	0.1	0	1.5	0	0	0	0	0	0	0	1.2	0.1	0	0	0	0	0	0.5	0.5	0	0
**GGT1477;HOT307**	*Prevotella salivae*	0.1	0.7	0	0.2	0	0	0	0.1	0.9	0	0	0	0	0	0	0.1	0	0	0	0.1	0.1	0.1	0	0.3
**HOT893**	*Actinomyces oris*	0	0	2.2	0.1	5.2	0	0	0	0.1	0.1	0	0	0	0	0	0	0	0	0	0	0.1	0.5	0	0
**HOT346**	*TM7_[G-1] sp._Oral_Taxon_346*	0.2	1.1	0	0.4	0	0.1	0	0.1	0	0	0	0	0.7	0	0	0	0	0	0	0	0	0	0	0.9
**HOT317**	*Prevotella sp._Oral_Taxon_317*	0	0	0	0	0	0.5	0	0	0	0	0.2	3.9	0	0.1	0	0	0	0	0	0	0.1	0	0	0
**GGT1624;HOT587**	*Rothia dentocariosa*	0	0	0.1	0.2	0.4	1.1	1.4	0.4	0.1	0.4	0	0	0	0	0.1	0.8	0.2	0.1	0.2	0.2	0.2	0.5	0.1	0
**HOT609**	*Neisseria flava*	0.3	2.5	0.1	0.1	0	0	0	0.1	0.1	0	0	0	0	0.8	0.8	0	0	0	0	0	0	0	0	0
**HOT284**	*Porphyromonas sp._Oral_Taxon_284*	0	0	0.5	1.9	0	0.8	0	0	0	0	0	0	0	0	0	0	0	0	0	0	1.8	1.9	0	0
**GGT808;HOT202**	*Fusobacterium nucleatum_ss_polymorphum*	0	0	0.1	0.2	0	0.4	0	0	0	0	0.1	0.8	0.7	1.8	0.1	0.2	0.1	0.4	0	0	0.1	0.3	0	0
**GGT1794;HOT543**	*Streptococcus anginosus*	3.4	1.3	0	0	0	0	0	0	0	0	0.1	0.3	0	0	0.1	0	0	0	0	0	0	0	0	0.5
**HOT498**	*Leptotrichia sp._Oral_Taxon_498*	0	0	0	0	0	0	0	0	0	0	0	0	0.1	0	0	0	0	0	0	0	0	0	0	0
**GGT1807;HOT638**	*Streptococcus infantis*	0	0	0.1	0.1	0.1	0.3	0	0.9	0.1	0.1	0.2	0.8	0	0	0	0	0	0	0	0	0.1	0	0	0

**HOT061**	*Streptococcus sp._Oral_Taxon_061*	0	0	0.1	0.1	0.1	0.2	0	0.6	0.1	0.1	0	0.2	0.1	0	0.1	0	0.2	0.6	0	0	0	0	0	0.1
**HOT283**	*Porphyromonas catoniae*	0	0	0	0	0	1.7	0	0	0	0	0.1	0	0	0.5	0.2	0	0	0	0	0	0.5	0.3	0	0
**HOT275**	*Porphyromonas sp._Oral_Taxon_275*	0	0	0.2	0.6	0	0	0	0	0	0	0	0	0	5.7	0	0	0	0	0	0	0.1	0	0	0
**GGT1035;HOT608**	*Lactobacillus fermentum*	0	0	0	0	0	0	0.1	0.1	0.2	0.4	0	0	0	0	0	0	0	0	2.8	1.8	0.1	0	0	0
**HOT446**	*Actinomyces sp._Oral_Taxon_446*	0	0	0	0	0	0	0	0	0	0	0	0	0	0.1	0.1	0.6	0.5	0.6	0.1	1	0.3	0.4	0	0
**HOT348**	*TM7_[G-1] sp._Oral_Taxon_348*	0	0	0	0.2	0	0.2	0	0	0	0	0	0	0.5	0.3	0	0	0	0	0	0	0.7	0.1	0	0
**GGT1625;HOT188**	*Rothia aeria*	0	0	0.2	0.1	0.8	0.1	0.2	0.2	0.1	0.1	0.1	0	1	0	0	0.1	0.1	0.1	0	0	0.5	0.7	0	0
**HOT056**	*Streptococcus sp._Oral_Taxon_056*	0	0	0	0	0	0	0.1	0.1	0	0	2.3	0.6	0.1	0	0.1	0	0.1	0	0	0	0.2	0.7	0	0.1
**HOT175**	*Actinomyces sp._Oral_Taxon_175*	1	1.4	0.3	0.1	0.2	0.3	0	0.1	0	0	0	0	0	0.2	0.1	0.1	0	0	0	0	0.2	0.3	0	0
**GGT999;HOT706**	*Kingella oralis*	0	0.1	0.2	0	0	0	0	0	0	0	0	0	0	0	0	0.1	0	0	0	0	0.8	2.5	0	0
**GGT397;HOT633**	*Cardiobacterium hominis*	0	0	0	0	0	0	0	0	0	0	0.3	0.9	0.4	0	0	0.3	0	0.1	0	0	0	0	0	0
**GGT1002;HOT731**	*Klebsiella pneumoniae*	0	0	0	0	0	0	0	0	0	0	0	0	0	0	0	0	0	0	0	0	0	0	0	0
**HOT618**	*Actinomyces gerencseriae*	0	0	0	0	0	0	0	0	0	0	0	0.1	0.3	0	0	0.8	0	0	0	0	0	0	0	0
**HOT849**	*Actinomyces johnsonii*	0	0	0.2	1	0	0	0	0	0	0	0	0	0.1	1.4	0.2	0	0	0	0	0	0	0	0	0
**HOT596**	*Granulicatella elegans*	0.1	0.4	0	0	0	0.1	0.1	0.2	0.2	0.4	0	0	0	0	0.2	0.2	0.1	0.2	0	0	0.2	0.2	0.1	0.1
**GGT1808;HOT6**	*Streptococcus intermedius*	0.5	0.4	0	0	0.1	0	0	0	0	0	0	0.1	0	0	0	0	0	0	0.1	0.2	0.1	0	0	0.8

**44**																									
**HOTE75**	*Actinomyces sp._Oral_Taxon_E75*	0.4	0.5	0	0	0	0	0	0	0	0	0.2	0.2	0	0	0	0	0	0	0	0	0	0	0	1.8
**GGT46; HOT176**	*Actinomyces naeslundii*	0	0	0.1	0	0.1	0.1	0.2	0.1	0.1	0.5	0.1	0	0.1	0.1	0	0.2	0.1	0.5	0	0.1	0.1	0	0	0
**GGT1027;HOT819**	*Lactobacillus johnsonii*	0	0	0	0	0	0	0	0	0	0	0.2	0	0	0	0	0	0	0	0	0.1	0	0	0	0
**HOTG60**	*Prevotella sp._Oral_Taxon_G60*	0.1	0.1	0	0.3	0	0	0	0.1	0.3	0	0	0.3	0	0.2	0	0	0	0	0	0	0	0	0	0.1
**GGT1482;HOT572**	*Prevotella veroralis*	0	4.1	0	0	0	0	0	0	0	0	0	0	0	0	0	0	0	0	0	0	0	0	0	0
**HOT494**	*Lachnoanaerobaculum saburreum*	0	0	0.2	0.1	0	0.1	0	0	0	0	0	1	1.6	0.1	0	0	0	0	0	0	0.1	0.1	0	0
**HOT286**	*Tannerella sp._Oral_Taxon_286*	0.1	0	0	0.1	0	0.1	0	0	0	0	0	0.8	0.6	0.1	0	0.2	0	0.1	0	0	0	0	0	0
**HOT300**	*Prevotella sp._Oral_Taxon_300*	0.8	0.4	0	0	0	0	0	0	0	0	0	0	0	0	0	0	0.1	0	0	0	0	0	0	0.2
**GGT675;HOT118**	*Dialister invisus*	1.7	1.2	0	0	0	0	0	0	0	0	0	0	0	0	0	0	0	0	0	0	0.1	0	0	0.1
**GGT1681;HOT130**	*Selenomonas noxia*	0	0	0.1	0.1	0	0	0	0	0.1	0	0.1	0.8	0.3	0.1	0	0	0	0	0	0	0.1	0	0	0.1
**GGT155;HOT723**	*Atopobium parvulum*	0.2	0	0.3	0	0.4	0	0	0.1	0.1	0	0	0.1	0.1	0	0	0	0	0	0	0.3	0	0	0	0.2
**GGT376;HOT575**	*Campylobacter concisus*	0	0	0.3	0.6	0.1	0.1	0	0.2	0.1	0	0	0.1	0.1	0.1	0	0	0	0.1	0	0.2	0.2	0.2	0	0
**GGT1031;HOT615**	*Lactobacillus gasseri*	0.1	0.2	0	0	0	0	0	0.2	0.4	0.7	0	0	0	0	0	0.1	0	0	0.6	1.7	0	0	0	0
**HOT431**	*Streptococcus sp._Oral_Taxon_431*	0	0	0.1	0.1	0.1	0.3	0.1	0.7	0.1	0.5	0.1	0.2	0	0	0	0	0	0	0	0.1	0	0	0	0
**GGT1078;HOT2**	*Leptotrichia hofstadii*	0	0	0	0.1	0	0.1	0	0	0	0	0	0.1	2.3	0	0	0	0	0	0	0	0	0.1	0	0

**24**																									
**GGT388;HOT325**	*Capnocytophaga granulosa*	0	0	0	0	0	0.1	0	0	0	0	0.1	0.7	1.2	0	0	0	0	0	0	0	0.1	0.1	0	0
**HOT329**	*Capnocytophaga leadbetteri*	0	0	0.1	0.1	0	0.3	0	0	0	0	0.1	0.3	0.7	0.1	0	0	0	0	0	0	0.1	0	0	0
**GGT157;HOT750**	*Atopobium rimae*	1.6	0.1	0	0	0	0	0	0	0	0	0.1	0.1	0	0	0	0	0	0	0	0	0	0	0	0.2
**GGT816;HOT626**	*Gemella haemolysans*	0.1	0.7	0	0	0	0.8	0	0	0	0	0	0	0	0	0	0	0	0	0	0	0.1	0.1	0	0
**HOT458**	*Aggregatibacter sp._Oral_Taxon_458*	0	0	0	0.2	0	0.8	0	0	0	0	0	0	0.2	0.3	0.1	0	0	0	0	0	0.8	0.4	0	0
**GGT1815;HOT073**	*Streptococcus australis*	0	0	0	0	0	0.1	0	0.2	0.1	0	0	0.1	0.2	0	0	0	0	0	0	0.1	0	0	0	0
**GGT1485;HOT311**	*Prevotella oris*	1.9	0.6	0	0	0	0	0	0	0	0	0	0	0	0	0	0	0	0	0	0	0	0	0	0
**GGT1627;HOT681**	*Rothia mucilaginosa*	0	0	0	0	0.2	0.7	0	0.2	0	0	0	0	0	0	0.2	0	0	0	0	0	0.1	0.1	0	0
**HOT313**	*Prevotella sp._Oral_Taxon_313*	0	0	0	0	0	0	0	0	0	0	0	0	0	0	0	0	0	0	0	0	0	0	0	0
**HOT322**	*Bergeyella sp._Oral_Taxon_322*	0	0	0	0.1	0	0.2	0	0	0	0	0	0.1	0.1	0.1	0.1	0	0	0.1	0	0	0.4	0.5	0.1	0
**HOT540**	*Cardiobacterium valvulum*	0	0	0	0	0	0	0	0	0	0	1.1	1.1	0.2	0.1	0	0	0	0	0	0	0	0	0	0
**GGT1473;HOT288**	*Prevotella oulorum*	0.3	0.3	0	0.1	0	0	0	0	0	0	0	0.3	0	0.1	0	0	0	0	0	0	0	0	0	0.2
**HOTB43**	*Porphyromonas sp._Oral_Taxon_B43*	0	0	0.1	0.2	0	0	0	0	0	0	0	0	0	0.1	0	0	0	0	0	0	1	1.2	0	0
**GGT392;HOT775**	*Capnocytophaga sputigena*	0.5	0	0.2	0.2	0	0.1	0	0	0	0	0.1	0.1	0.1	0	0	0	0	0	0	0	0.3	0.4	0	0
**HOT419**	*Moryella*	0.1	0.2	0.1	0	0	0.1	0	0	0	0	0	0	0	0	0	0	0	0	0	0	0	0	0	0

	sp._Oral_Taxon_419																								
**HOTF95**	*Capnocytophaga sp._Oral_Taxon_F95*	0	0	0	0	0	0	0	0	0	0	0.6	1.9	0	0	0	0	0	0	0	0	0	0	0	0
**GGT1486;HOT714**	*Prevotella pallens*	0	0	0	0	0	0	0	0.1	0	0	0	0	0	0	0	0	0	0	0	0	0	0	0	0.1
**HOT335**	*Capnocytophaga sp._Oral_Taxon_335*	0	0	0	0	0	0	0	0	0	0	0.4	1.9	0	0	0	0	0	0	0	0	0	0	0	0
**GGT2038**	*Yersinia mollaretii*	0	0	0	0	0	0	0	0	0	0	0	0	0.1	0	0	0	0	0	0	0	0	0	0	0.2
**GGT1788;HOT576**	*Streptococcus constellatus*	0.4	0.4	0	0	0	0	0	0.1	0	0	0	0	0	0	0	0	0	0	0	0	0	0	0	0
**HOT347**	*TM7_[G-1] sp._Oral_Taxon_347*	0	0	0	0	0	0	0	0	0	0	0	0	0	0	0	0.1	0	0	0	0	0.1	0	0	0
**GGT53;HOT701**	*Actinomyces odontolyticus*	0	0	0.2	0.1	0.2	0.1	0	0.2	0.1	0	0.1	0.1	0	0.1	0.1	0.1	0	0	0	0	0.1	0.1	0	0
**GGT1022;HOT817**	*Lactobacillus crispatus*	0	0	0	0	0	0	0	0	0	0	0.2	0	0	0	0	0	0	0	0	0	0	0	0	0
**HOT074**	*Streptococcus sp._Oral_Taxon_074*	0	0	0	0.1	0	0.2	0	0.1	0	0	0	0.1	0	0	0	0	0.1	0.1	0.1	0.2	0	0	0	0
**GGT706;HOT577**	*Eikenella corrodens*	0	0	0	0	0	0.1	0	0	0	0	0.1	0.2	0	0.1	0	0.1	0	0.1	0	0	0	0	0	0
**GGT711**	*Enterobacter cloacae*	0	0	0	0	0	0	0	0	0	0	0.1	0.1	0.1	0	0	0	0	0	0	0	0	0	0	0
**GGT1811;HOT721**	*Streptococcus parasanguinis_I*	0	0	0	0	0	0.1	0	0.6	0	0.1	0	0	0	0	0	0	0	0	0	0	0	0	0	0
**HOTH27**	*Fusobacterium sp._Oral_Taxon_H27*	0.2	0.2	0	0	0	0	0	0	0	0	0	0	0	0	0	0	0	0	0	0	0	0	0	0
**HOT888**	*Actinomyces dentalis*	0	0	0.1	0	0	0.1	0	0.1	0	0	0	0	0	0	0	0	0.1	0	0	0	0	0	0	0.1
**GGT157**	*Ralstonia*	0	0	0	0	0	0	0	0	0	0	0.1	0	0	0	0	0	0	0	0	0	0	0	0	0

**0;HOT854**	*pickettii*																							
**HOT351**	*TM7_[G-3] sp._Oral_Taxon_351*	0.6	0.4	0	0	0	0	0	0	0	0	0	0	0	0	0	0	0	0	0	0	0	0	0	0
**HOT097**	*Moryella sp._Oral_Taxon_097*	0.1	0.1	0.2	0	0	0.1	0	0	0	0	0	0	0	0	0	0	0	0	0	0	0	0	0	0
**HOT412**	*Capnocytophaga sp._Oral_Taxon_412*	0	0	0	0	0	0	0	0	0	0	0	0	0.9	0	0	0	0	0	0	0	0.3	0.4	0	0
**HOT326**	*Capnocytophaga sp._Oral_Taxon_326*	0	0	0.1	0.1	0	0	0	0	0	0	0	0.3	0	0	0	0	0	0	0	0	0	0	0	0
**GGT1074;HOT563**	*Leptotrichia buccalis*	0	0	0	0	0	0.1	0	0	0	0	0	0	0.3	0	0	0	0	0	0	0	0	0	0	0
**GGT1000;HOT582**	*Kingella denitrificans*	0.1	0	0	0	0	0	0	0	0	0	0	0	0.4	0.1	0	0	0	0	0	0	0	0.1	0	0
**HOT461**	*Lactobacillus sp._Oral_Taxon_461*	0.1	1.3	0	0	0	0	0	0	0	0	0	0	0	0	0	0	0	0	0	0	0	0	0	0
**HOTD12**	*Dietzia sp._Oral_Taxon_D12*	0	0	0	0	0	0	0	0	0	0	0	0	0	0	0	0	0	0	0	0	0	0	0	1.2
**GGT2084;HOT155**	*Veillonellaceae_[G-1] sp._Oral_Taxon_155*	0	0	0	0	0	0	0	0.1	0	0	0	0	0	0	0	0	0	0	0	0	0	0	0	0
**GGT1361;HOT807**	*Olsenella sp._Oral_Taxon_807*	0	0.1	0	0	0	0	0	0	0	0	0	0	0.5	0	0	0	0	0	0	0	0	0	0	0
**HOT131**	*Mitsuokella sp._Oral_Taxon_131*	0	0	0	0	0	0.1	0	0	0	0	0	0	0.1	0	0	0.1	0.1	0.2	0.1	0.1	0	0	0	0.3
**GGT1041;HOT051**	*Lactobacillus vaginalis*	0	0	0	0	0	0	0	0	0	0	0	0	0	0	0	0	0	0	1	0.3	0	0	0	0
**HOT336**	*Capnocytophaga sp._Oral_Taxo*	0	0	0.1	0.1	0	0.1	0	0	0	0	0	0	0.3	0	0	0	0	0.2	0	0	0	0	0	0

	n_336																								
**GGT1362;HOT806**	*Olsenella profusa*	0	0	0.2	0	0.3	0	0	0	0.1	0	0	0	0	0	0	0.1	0	0	0	0	0	0	0	0
**GGT573;HOT579**	*Cryptobacterium curtum*	0.1	0.3	0	0	0	0	0	0	0	0	0	0	0.1	0	0	0	0	0	0	0	0	0	0	0.2
**GGT1989;HOT524**	*Veillonella atypica*	0	0	0	0	0	0	0	0	0	0	0	0	0	0	0	0	0	0	0	0.4	0	0	0	0
**HOT473**	*Alloprevotella sp._Oral_Taxon_473*	0	0.1	0	0	0	0.3	0	0	0	0	0	0	0	0	0	0	0	0	0	0	0	0	0	0
**GGT1818;HOT021**	*Streptococcus vestibularis*	0	0.2	0	0	0	0	0	0	0	0	0	0	0	0	0	0	0.6	0	0.1	0.2	0	0	0	0
**HOTE78**	*Streptococcus sp._Oral_Taxon_E78*	0	0	0	0	0	0	0.1	0	0	0.1	0.2	0.1	0	0	0	0	0	0	0	0	0.1	0	0	0
**GGT806;HOT201**	*Fusobacterium periodonticum*	0	0	0	0.1	0	0.2	0	0	0	0	0	0	0	0.1	0	0	0	0	0	0	0	0	0	0
**GGT1114;HOT122**	*Megasphaera micronuciformis*	0	0.1	0	0	0	0	0	0	0.1	0	0	0	0	0	0	0	0	0	0	0	0.1	0	0	0
**HOT739**	*Propionibacterium propionicum*	0	0	0	0	0	0	0	0	0	0	0	0	0	0	0	0.1	0.1	0	0	0	0.2	0.4	0	0
**GGT813;HOT757**	*Gemella sanguinis*	0	0	0	0.1	0	0.1	0	0.1	0	0	0	0	0	0	0	0	0	0	0	0	0	0	0	0
**HOT660**	*Defluvibacter lusatiensis*	0	0	0	0	0	0	0	0	0	0	0	0	0	0	0	0	0	0	0	0	0	0	0	1.1
**HOT057**	*Streptococcus sp._Oral_Taxon_057*	0	0	0	0	0	0	0	0.1	0	0	0	0.2	0	0	0	0	0	0	0.1	0	0	0	0	0
**GGT530;HOT595**	*Corynebacterium durum*	0	0	0	0	0	0.1	0	0	0	0	0	0	0	0	0	0	0	0	0	0	0	0.2	0	0
**HOT324**	*Capnocytophaga sp._Oral_Taxon_324*	0	0	0.2	0.1	0	0	0	0	0	0	0	0	0	0.1	0	0	0	0.1	0	0	0	0	0	0
**GGT175**	*Staphylococcu*	0	0	0	0	0	0	0	0	0	0	0	0	0	0	0	0	0	0	0	0	0	0	0	0

**8**	*s equorum*																								
**GGT1367;HOT457**	*Oribacterium sinus*	0	0	0.1	0.1	0	0	0	0.1	0	0	0	0	0	0	0	0	0	0	0	0	0.1	0	0	0.1
**HOTA58**	*Acinetobacter sp._Oral_Taxon_A58*	0	0	0	0	0	0	0	0	0	0	0	0	0	0	0	0	0	0	0	0	0	0	0	0
**GGT1608**	*Riemerella anatipestifer*	0	0	0	0	0	0	0	0	0	0	0	0	0	0	0	0	0	0	0	0	0	0	0	0
**HOT870**	*TM7_[G-1] sp._Oral_Taxon_870*	0	0	0	0	0	0	0	0	0	0	0	0	0	0	0	0	0	0	0	0	0	0	0	0
**HOT278**	*Porphyromonas sp._Oral_Taxon_278*	0	0	0	0	0	0.7	0	0	0	0	0	0	0	0	0	0	0	0	0	0	0	0	0	0
**HOTA88**	*Pseudomonas mosselii_Oral_Taxon_A88*	0	0	0	0	0	0	0	0	0	0	0.5	0	0	0	0	0	0	0	0	0	0	0	0	0
**GGT1304;HOT598**	*Neisseria elongata*	0	0	0	0	0	0.1	0	0	0	0	0	0	0.2	0.1	0	0	0	0	0	0	0	0	0	0
**HOT078**	*Oribacterium sp._Oral_Taxon_078*	0.5	0.1	0	0	0	0	0	0	0	0	0	0	0	0	0	0	0	0	0	0.1	0	0	0	0
**HOT352**	*TM7_[G-1] sp._Oral_Taxon_352*	0	0	0	0	0	0	0	0.1	0	0	0	0	0	0	0	0	0	0	0	0	0	0	0	0
**GGT1488;HOT299**	*Prevotella sp._Oral_Taxon_299*	0	0	0.1	0.1	0.1	0.1	0	0	0	0	0	0	0	0	0	0	0	0	0	0	0	0.1	0	0
**HOT308**	*Alloprevotella sp._Oral_Taxon_308*	0	0	0	0	0	0.1	0	0	0	0	0	0.1	0	0	0	0	0	0	0	0	0.1	0.1	0	0
**GGT1536;HOT612**	*Pseudomonas fluorescens*	0	0	0	0	0	0	0	0	0	0	0	0	0.1	0	0	0	0	0	0.2	0	0	0	0	0.1
**GGT1820**	*Streptococcus sp._str._M334*	0	0	0	0	0	0	0.3	0	0.1	0.1	0	0	0	0	0	0	0.1	0	0	0	0	0	0	0
**HOT096**	*Lachnospiraceae_[G-2] sp._Oral_Taxon_096*	0	0	0.5	0.1	0	0	0	0	0	0	0	0	0	0	0	0	0	0	0	0	0	0	0	0
**GGT149**	*Prevotella*	0.4	0	0	0	0	0	0	0	0	0	0	0	0	0	0	0	0	0	0	0	0	0	0	0

**0;HOT289**	*maculosa*																								
**GGT1498;HOT466**	*Alloprevotella tannerae*	0	0	0	0	0	0	0	0	0	0	0	0	0	0	0	0	0	0	0	0	0	0	0	0
**GGT1043;HOT756**	*Lactobacillus salivarius*	0	0	0	0	0	0	0	0	0	0	0	0	0	0	0	0	0	0	0.3	0.2	0	0	0	0
**GGT33**	*Acinetobacter sp._str._DR1*	0	0	0	0	0	0	0	0	0	0	0	0	0	0	0	0	0	0	0	0	0	0	0	0
**GGT1077;HOT214**	*Leptotrichia shahii*	0	0	0	0	0	0	0	0	0	0	0	0	0	0	0	0	0	0	0	0	0	0	0	0
**GGT380;HOT763**	*Campylobacter showae*	0	0	0	0	0	0	0	0	0	0	0	0.1	0.1	0	0	0	0	0	0	0	0	0	0	0
**HOT107**	*Lachnoanaerobaculum umeaense*	0.1	0.1	0.1	0	0	0	0	0	0	0	0	0.1	0	0	0	0	0	0	0	0	0	0	0	0
**HOT221**	*Leptotrichia sp._Oral_Taxon_221*	0	0	0.1	0.2	0	0	0	0	0	0	0	0	0	0.1	0	0	0	0	0	0.1	0.1	0.1	0	0
**HOT349**	*TM7_[G-1] sp._Oral_Taxon_349*	0	0	0	0	0	0	0	0	0	0	0	0	0	0	0	0	0	0	0	0	0	0	0	0
**HOTE20**	*Selenomonas sp._Oral_Taxon_E20*	0	0	0	0	0	0	0	0	0	0	0	0	0	0	0	0	0	0	0	0	0	0	0	0
**HOT100**	*Lachnospiraceae_[G-3] sp._Oral_Taxon_100*	0	0	0	0	0	0	0	0	0	0	0	0	0.1	0	0	0	0	0	0	0	0	0	0	0
**HOT887**	*Veillonella denticariosi*	0	0	0	0	0	0	0	0	0	0	0	0	0	0	0	0	0	0	0	0	0	0	0	0
**HOT215**	*Leptotrichia sp._Oral_Taxon_215*	0	0	0.1	0	0	0.1	0	0	0	0	0	0	0	0	0	0	0	0	0	0	0	0	0	0
**GGT1034**	*Lactobacillus helveticus*	0	0	0	0	0	0	0	0	0	0	0.1	0	0	0	0	0	0	0	0	0	0	0	0	0
**HOT900**	*Bergeyella sp._Oral_Taxon_900*	0	0	0	0	0	0	0	0	0	0	0	0	0.2	0	0	0	0	0	0	0	0	0	0	0
**GGT803;HOT20**	*Fusobacterium*	0	0	0	0	0	0	0	0	0	0	0	0	0	0.3	0	0	0	0	0	0	0	0	0	0

**0**	*nucleatum_ss _vincentii*																								
**HOT323**	*Capnocytophaga sp._Oral_Taxon_323*	0	0	0	0	0	0	0	0	0	0	0	0	0	0	0	0	0	0	0	0	0	0	0	0
**HOTE63**	*Actinomyces sp._Oral_Taxon_E63*	0	0	0	0	0	0.2	0	0	0	0	0	0	0	0	0	0	0	0	0	0	0	0	0	0
**HOT913**	*Alloprevotella sp._Oral_Taxon_913*	0	0	0	0	0	0	0	0	0	0	0	0	0	0	0	0	0	0	0	0	0	0	0	0
**HOT886**	*Streptococcus oligofermentans*	0	0	0	0	0	0	0	0	0	0	0	0	0.4	0	0	0	0	0	0	0	0	0	0	0
**HOT423**	*Streptococcus sp._Oral_Taxon_423*	0	0	0	0	0	0	0	0	0	0	0	0	0	0	0	0.1	0	0	0	0	0	0	0	0
**GGT1813**	*Streptococcus sp._Oral_Taxon_71*	0	0	0	0	0	0	0	0	0	0	0	0	0	0	0.1	0	0	0	0	0	0	0	0	0
**HOT181**	*Actinomyces sp._Oral_Taxon_181*	0	0	0	0	0	0.1	0	0.1	0	0	0	0	0	0	0	0	0	0	0	0	0	0	0	0
**HOTE28**	*Streptococcus sp._Oral_Taxon_E28*	0	0	0	0	0	0	0	0	0	0	0	0	0	0	0	0	0	0	0	0	0	0	0	0
**HOTH23**	*Selenomonas sp._Oral_Taxon_H23*	0	0	0	0	0	0	0	0	0	0	0	0	0	0	0	0	0	0	0	0	0	0	0	0
**HOT769**	*Treponema socranskii_ss_socranskii*	0.2	0.1	0	0	0	0	0	0	0	0	0	0	0	0	0	0	0	0	0	0	0	0	0	0
**HOT874**	*SR1_[G-1] sp._Oral_Taxon_874*	0	0	0	0	0	0	0	0	0	0	0	0	0	0	0	0	0	0	0	0	0	0	0	0
**GGT387;HOT700**	*Capnocytophaga ochracea*	0	0	0	0	0	0	0	0	0	0	0.1	0.3	0	0.1	0	0	0	0	0	0	0	0	0	0
**GGT1676;HOT151**	*Selenomonas sputigena*	0.1	0	0	0	0	0	0	0	0	0	0	0	0	0	0	0	0	0	0	0	0	0	0	0.1
**GGT1479;HOT7**	*Prevotella multisaccharv*	0	0	0	0	0	0	0	0	0	0	0	0	0	0	0	0	0	0	0	0	0	0	0	0

**94**	*orax*																								
**GGT1821;HOT728**	*Streptococcus peroris*	0	0	0	0	0	0.1	0	0	0	0	0	0	0	0	0	0	0	0	0	0	0	0	0	0
**GGT1432;HOT111**	*Parvimonas micra*	0.3	0	0	0	0	0	0	0	0	0	0	0	0	0	0	0	0	0	0	0	0	0	0	0
**GGT1302;HOT729**	*Neisseria pharyngis*	0	0	0	0	0	0	0	0	0	0	0	0	0	0	0	0	0	0	0	0	0	0	0	0
**HOT487**	*Streptococcus sp._Oral_Taxon_487*	0	0	0	0	0	0.1	0	0	0	0	0	0	0	0	0	0	0	0	0	0	0	0	0	0
**HOT808**	*Tannerella sp._Oral_Taxon_808*	0.1	0	0	0	0	0	0	0	0	0	0	0	0	0	0	0	0	0	0	0	0	0	0	0
**HOTA16**	*Corynebacterium sp._Oral_Taxon_A16*	0	0	0	0	0	0	0.1	0.1	0	0	0	0	0	0	0	0	0	0	0	0	0	0.1	0	0
**HOT565**	*Enterobacter cancerogenus*	0	0	0	0	0	0	0	0	0	0	0.1	0	0	0	0	0	0	0	0	0	0	0	0	0
**GGT1037;HOT818**	*Lactobacillus reuteri*	0	0	0	0	0	0	0	0	0	0	0	0	0	0	0	0	0	0	0	0	0	0	0	0
**GGT306**	*Brevibacillus brevis*	0	0	0	0	0	0	0	0	0	0	0.1	0	0	0	0	0	0	0	0	0	0	0	0	0
**HOT914**	*Alloprevotella sp._Oral_Taxon_914*	0	0	0	0	0	0.1	0	0	0	0	0	0	0	0	0	0	0	0	0	0	0	0	0	0
**HOT225**	*Leptotrichia sp._Oral_Taxon_225*	0	0	0	0	0	0	0	0	0	0.1	0	0	0	0	0	0	0	0	0	0	0	0.1	0	0
**HOT203**	*Fusobacterium sp._Oral_Taxon_203*	0	0.1	0	0	0	0	0	0	0	0	0	0	0	0	0	0	0	0	0	0	0	0	0	0
**HOTG67**	*Selenomonas sp._Oral_Taxon_G67*	0	0	0	0	0	0	0	0	0	0	0	0.2	0	0	0	0	0	0	0	0	0	0	0	0
**GGT10;HOT343**	*Achromobacter xylosoxidans*	0	0	0	0	0	0	0	0	0	0	0	0	0	0	0	0	0	0	0	0	0	0	0	0.3
**HOTF11**	*Streptococcus sp._Oral_Taxo*	0	0	0	0	0	0	0	0	0	0	0	0	0	0	0	0	0	0	0	0	0	0	0	0

	n_F11																							
**GGT1026;HOT749**	*Lactobacillus rhamnosus*	0	0	0	0	0	0	0	0	0	0	0	0	0	0	0	0	0	0	0	0	0	0	0	0
**GGT76; HOT762**	*Aggregatibacter segnis*	0	0	0	0	0	0	0	0	0	0	0	0	0	0.1	0	0	0	0	0	0	0	0	0	0
**GGT863;HOT851**	*Haemophilus haemolyticus*	0	0	0	0	0	0.1	0	0	0	0	0	0	0	0	0	0	0	0	0	0	0	0	0	0
**HOT912**	*Alloprevotella sp._Oral_Taxon_912*	0	0	0	0	0	0	0	0	0	0	0	0	0	0	0	0	0	0	0	0	0	0	0	0
**HOT557**	*Eubacterium_[XI][G-3] brachy*	0.2	0.1	0	0	0	0	0	0	0	0	0	0	0	0	0	0	0	0	0	0	0	0	0	0
**HOTC21**	*Kingella sp._Oral_Taxon_C21*	0	0	0	0	0	0	0	0	0	0	0	0	0	0	0	0	0	0	0	0	0.1	0.2	0	0
**GGT2;HOT165**	*Catonella morbi*	0.1	0	0	0	0	0	0	0	0	0	0	0	0	0	0	0	0	0	0	0	0	0	0	0
**HOT306**	*Prevotella sp._Oral_Taxon_306*	0	0	0	0	0	0	0	0	0.1	0	0	0	0	0	0	0	0	0	0	0	0	0	0	0
**GGT1020;HOT568**	*Lactobacillus casei*	0	0	0	0	0	0	0	0	0	0	0	0	0	0	0.1	0	0	0	0	0	0	0	0	0
**HOTE72**	*Streptococcus sp._Oral_Taxon_E72*	0	0	0	0	0	0	0	0	0	0	0	0	0	0	0	0	0	0	0	0	0	0	0	0
**HOT075**	*Clostridiales_[F-2][G-1] sp._Oral_Taxon_075*	0.1	0	0	0.1	0	0	0	0	0	0	0	0	0	0	0	0	0	0	0	0	0	0	0	0
**GGT1534;HOT536**	*Pseudomonas aeruginosa*	0	0	0	0	0	0	0	0	0	0	0	0	0	0	0	0	0	0	0	0	0	0	0	0.2
**HOTA56**	*TM7_[G] sp._Oral_Taxon_A56*	0	0	0	0	0	0	0	0.1	0	0	0	0	0	0	0	0	0	0	0	0	0	0	0	0
**HOT124**	*Selenomonas artemidis*	0	0	0	0	0	0	0	0	0	0	0	0	0	0	0	0	0	0	0	0	0	0	0	0
**HOT865**	*Kluyvera ascorbata*	0	0	0	0	0	0	0	0	0	0	0	0	0	0	0	0	0	0	0	0	0	0	0	0
**HOT305**	*Prevotella*	0	0	0	0	0	0	0	0	0.2	0	0	0	0	0	0	0	0	0	0	0	0	0	0	0

	sp._Oral_Taxon_305																								
**HOT353**	*TM7_[G-1] sp._Oral_Taxon_353*	0	0	0	0	0	0	0	0	0	0	0	0	0	0	0	0	0	0	0	0	0	0	0	0
**HOTF93**	*Lachnospiraceae_[G] sp._Oral_Taxon_F93*	0	0	0	0	0	0	0	0	0	0	0	0.2	0	0	0	0	0	0	0	0	0	0	0	0
**GGT78; HOT485**	*Agrobacterium tumefaciens*	0	0	0	0	0	0	0	0	0	0	0	0	0	0	0	0	0	0	0	0	0	0	0	0.2
**HOT303**	*Prevotella pleuritidis*	0	0	0	0	0	0	0	0	0	0	0	0	0	0	0	0	0	0	0	0	0	0	0	0
**HOT121**	*Anaeroglobus geminatus*	0	0	0	0	0	0	0	0	0	0	0	0	0	0	0	0	0	0	0	0	0	0	0	0
**HOT212**	*Leptotrichia sp._Oral_Taxon_212*	0	0	0	0	0	0	0	0	0	0	0	0.1	0	0	0	0	0	0	0	0	0	0	0	0
**HOT082**	*Lachnoanaerobaculum orale*	0	0	0.1	0	0	0	0	0	0	0	0	0	0	0	0	0	0	0	0	0	0	0	0	0
**HOTA46**	*Corynebacterium sp._Oral_Taxon_A46*	0	0	0	0	0	0.2	0	0	0	0	0	0	0	0	0	0	0	0	0	0	0	0	0	0
**GGT1723;HOT678**	*Solobacterium moorei*	0	0	0	0	0	0	0	0	0	0	0	0	0	0	0	0	0	0	0	0	0	0	0	0
**HOTC60**	*Manihot esculenta_Oral_Taxon_C60*	0	0	0	0	0	0	0	0	0	0	0	0	0	0	0	0	0	0	0	0	0	0	0	0
**HOTG62**	*Streptococcus sp._Oral_Taxon_G62*	0	0	0	0	0	0	0	0	0	0	0	0	0	0	0	0	0	0	0	0	0	0	0	0.1
**GGT1478;HOT693**	*Prevotella nigrescens*	0.1	0.1	0	0	0	0	0	0	0	0	0	0	0	0	0	0	0	0	0	0	0	0	0	0
**HOT085**	*Clostridiales_[F-2][G-2] sp._Oral_Taxon_085*	0	0.1	0	0	0	0	0	0	0	0	0	0	0	0	0	0	0	0	0	0	0	0	0	0
**GGT1680;HOT149**	*Selenomonas sp._Oral_Taxon_149*	0	0	0	0	0	0	0	0	0	0	0	0	0	0	0	0	0	0	0	0	0	0	0	0

**HOTG30**	*Veillonella sp._Oral_Taxon_G30*	0	0	0	0	0	0	0	0	0	0	0	0	0	0	0	0	0	0	0	0	0	0	0	0
**GGT673;HOT736**	*Dialister pneumosintes*	0	0	0	0	0	0	0	0	0	0	0	0	0	0	0	0	0	0	0	0	0	0	0	0
**HOT414**	*Actinomyces sp._Oral_Taxon_414*	0	0	0	0.1	0	0	0	0	0	0	0	0	0	0	0	0	0	0	0	0	0	0	0	0
**HOT781**	*Prevotella saccharolytica*	0	0	0	0	0	0	0	0	0	0	0	0	0	0	0	0	0	0	0	0	0	0	0	0
**HOT671**	*Actinomyces meyeri*	0	0.1	0	0	0	0	0	0	0	0	0	0	0	0	0	0	0	0	0	0	0	0	0	0
**GGT724;HOT803**	*Enterococcus italicus*	0	0	0	0	0	0	0	0	0	0	0	0	0	0	0	0	0	0	0	0	0	0	0	0
**GGT1497;HOT665**	*Prevotella marshii*	0.1	0	0	0	0	0	0	0	0	0	0	0	0	0	0	0	0	0	0	0	0	0	0	0
**GGT61;HOT178**	*Actinomyces sp._Oral_Taxon_178*	0	0	0	0	0	0	0	0	0	0	0	0	0.1	0	0	0	0	0	0	0	0	0	0	0
**GGT2076;HOT274**	*Bacteroidales_[G-2] sp._Oral_Taxon_274*	0	0.1	0	0	0	0	0	0	0	0	0	0	0	0	0	0	0	0	0	0	0	0	0	0
**HOTE05**	*Streptococcus sp._Oral_Taxon_E05*	0	0	0	0	0	0	0	0	0	0	0	0	0	0	0	0	0	0	0	0	0	0	0	0
**HOT223**	*Leptotrichia sp._Oral_Taxon_223*	0.1	0	0	0	0	0	0	0	0	0	0	0	0	0	0	0	0	0	0	0	0	0	0	0
**HOTE83**	*Selenomonas sp._Oral_Taxon_E83*	0	0	0	0	0	0	0	0	0	0	0	0	0	0	0	0	0	0	0	0	0	0	0	0.1
**HOT069**	*Streptococcus sp._Oral_Taxon_069*	0	0	0	0	0	0	0	0	0	0	0	0	0	0	0	0	0	0	0	0	0	0	0	0
**GGT1792**	*Streptococcus thermophilus*	0	0	0	0	0	0	0	0	0	0	0	0	0	0	0	0	0	0	0	0	0	0	0	0
**GGT765**	*Faecalibacterium prausnitzii*	0	0	0	0	0	0	0	0	0	0	0	0	0	0	0	0	0	0	0	0	0	0	0	0
**HOT613**	*Tannerella forsythia*	0	0	0	0	0	0	0	0	0	0	0	0	0	0	0	0	0	0	0	0	0	0	0	0

**HOT652**	*Afipia sp._genomospecies_4*	0	0	0	0	0	0	0	0	0	0	0	0	0	0	0	0	0	0	0	0	0	0	0	0
**HOT108**	*Oribacterium sp._Oral_Taxon_108*	0	0	0	0	0	0	0	0	0	0	0	0	0	0	0	0	0	0	0	0	0	0	0	0
**HOT350**	*TM7_[G-2] sp._Oral_Taxon_350*	0	0	0	0	0	0	0	0	0	0	0	0	0	0	0	0	0	0	0	0	0	0	0	0
**HOTD95**	*Fusobacterium sp._Oral_Taxon_D95*	0.1	0	0	0	0	0	0	0	0	0	0	0	0	0	0	0	0	0	0	0	0	0	0	0
**GGT1481**	*Prevotella copri*	0	0	0	0	0	0	0	0	0	0	0	0	0	0	0	0	0	0	0	0	0	0	0	0
**GGT1352;HOT544**	*Ochrobactrum anthropi*	0	0	0	0	0	0	0	0	0	0	0	0	0	0	0	0	0	0	0	0	0	0	0	0.1
**GGT1678;HOT125**	*Selenomonas flueggei*	0	0	0	0	0	0	0	0	0	0	0	0	0	0	0	0	0	0	0	0	0	0	0	0
**GGT710**	*Enterobacter sp._str._HCB*	0	0	0	0	0	0	0	0	0	0	0	0	0	0	0	0	0	0	0	0	0	0	0	0
**HOTH38**	*Cardiobacterium sp._Oral_Taxon_H38*	0	0	0	0	0	0	0	0	0	0	0	0	0.1	0	0	0	0	0	0	0	0	0	0	0
**GGT200**	*Bacteroides fragilis*	0	0	0	0	0	0	0	0	0	0	0	0	0	0	0	0	0	0	0	0	0	0	0	0
**GGT256;HOT588**	*Bifidobacterium dentium*	0	0	0	0	0	0	0	0	0	0	0	0	0	0	0	0	0	0	0	0	0	0	0	0
**HOTE49**	*Aggregatibacter sp._Oral_Taxon_E49*	0	0	0	0	0	0	0	0	0	0	0	0.1	0	0	0	0	0	0	0	0	0	0	0	0
**HOT213**	*Leptotrichia hongkongensis*	0	0	0	0	0	0	0	0	0	0	0	0	0	0	0	0	0	0	0	0	0	0	0	0
**GGT918**	*Halomonas sp._str._HTNK1*	0	0	0	0	0	0	0	0	0	0	0	0	0	0	0	0	0	0	0	0	0	0	0	0
**HOT866**	*Actinomyces graevenitzii*	0	0	0	0	0	0	0	0	0	0	0	0	0	0	0	0	0	0	0	0	0	0	0	0

**HOT600**	*Prevotella enoeca*	0	0	0	0	0	0	0	0	0	0	0	0	0	0	0	0	0	0	0	0	0	0	0	0
	**Column Sum**	**99.5**	**99.5**	**99.9**	**99.8**	**99.8**	**99.8**	**99.9**	**99.7**	**99.7**	**99.9**	**99.8**	**99.8**	**99.8**	**99.7**	**99.9**	**99.8**	**99.9**	**99.8**	**99.7**	**99.9**	**99.9**	**99.8**	**99.8**	**99.8**
