# Supplementary material for: The oral bacterial microbiome of occlusal surfaces in children and its association with diet and caries
Source: PLoS One. 2017 Jul 5;12(7):e0180621. doi: 10.1371/journal.pone.0180621 (PMC5498058; doi:10.1371/journal.pone.0180621)
Supplement: S5 Table — (PDF) [file pone.0180621.s007.pdf]

The Oral Bacterial Microbiome of Occlusal Surfaces in Children and its Association with Diet And Caries

S5 Table: Microbiome diversity measures in each taxonomic level, by clinical diagnosis categories

**Clinical diagnostic categories**					
	**Total**		**Sound**	**AWSL**	
**N**	**46**		**22**	**24**	
**α-diversity**					
	**Total**	**Dominant taxonomy[a]: RA variability (mRA±std)**	**Sound Total (mean±SD)**	**AWSL Total (mean±SD)**	***p*-value[b]**
**Species**	647	*Granulicatella paradiacens:* 0.9-48.4% (16.5%±12.0)<br>*Streptococcus mutans:* 0.0-55.2% (7.2%±10.5)<br>*Streptococcus sp._str._C300:* 0.2-27.3% (6.2%±5.7)<br>*Lautropia mirabilis:* 0.0-30.4% (4.2%±5.7)<br>*Pseudomonas sp._Oral_Taxon_C61:* 0.0-47.2% (4.8%±12.2)<br>*Streptococcus gordonii:* 0.0-21.5% (4.0%±4.8)<br>*Streptococcus sanguinis:* 0.2-9.9 (3.1%±2.4)<br>*Abiotrophia defectiva:* 0.0-54.7% (2.8%±8.3)<br>*Streptococcus oralis:* 0.0-35.9% (2.7%±6.3)<br>*Veillonella sp._Oral_Taxon_E53:* 0.0-14.2% (2.6%±2.8)	571 (197.8±63.6)	594 (217.4+33.4)	0.19
**Genera**	107	*Streptococcus:* 3.5-79.5% (33.2%±18.6)<br>*Granulicatella:* 1.0-50.0% (15.7%±12.9)<br>Klebsiella: 0.0-81.1% (9.6%±19.6)<br>*Pseudomonas:* 0.0-67.1% *(*5.6%±15.2)<br>*Actinomyces:* 0.1-17.3% (3.8%±3.7)<br>*Prevotella:* 0.0-43.4% (8.3%±3.6)<br>*Veillonella:* 0.1-31.4% (5.7%±4.4)<br>*Acinetobacter*: 0.0-76.2% (1.7%±11.1%)	65 (20.5±9.41)	61 (23.5±5.6)	0.19
**Families**	58	*Streptococcaceae:* 3.5-79.5% (33.2%±18.6)<br>*Carnobacteriaceae:* 1.0-50.0% (15.7%±12.9)<br>*Enterobacteriaceae:* 0.0-86.3% (11.6%±22.6)<br>*Pseudomonadaceae:* 0.0-67.5% (5.6%±15.2)<br>*Veillonellaceae:* 0.1-31.5% (4.8%±5.8)<br>*Actinomycetaceae:* 0.1-17.3% (4.0%±3.8)	38 (18.1±5.5)	30 (19.3±3.2)	0.36
**Orders**	29	*Lactobacillales:* 9.7-92.0% (52.3%±26.1)<br>*Enterobacteriales*: 0.0-86.3% (11.6%±22.6)<br>*Pseudomonadales:* 0.0-76.2% (7.3%±18.3)<br>*Actinomycetales*: 0.1-19.8% (5.1%±4.4)<br>*Clostridiales*: 0.1-31.8% (5.1%±5.9)<br>*Bacteroidales:* 0.0-44.6% (4.7%±8.7).	21 (12.1±3.2)	19 (13.0±1.9)	0.24
**Classes**	25	*Bacilli:* 9.7-92.0% (52.7%±26.1) *Gammaproteobacteria:* 0.0-86.3% (19.7%±29.5)<br>*Betaproteobacteria:* 0.0-76.2% (7.3%±18.3)<br>*Actinobacteria:* 0.1-19.8% (5.1%±4.4)<br>*Bacteroides:* 0.0-44.6% (4.7%±8.7)	15 (9.1±2.2)	11 (9.8±1.1)	0.14
**Phyla**	11	*Firmicutes:* 12.1-92.7 (57.8%±26.5)<br>*Proteobacteria:* 1.3-87.1 (28.5%±28.0)<br>*Actinobacteria*: 0.1-20.5 (5.7%±4.6)	9 (6.0±1.1)	7 (6.8±0.6)	**0.00**

		Bacteroidetes: 0.0-51.7 (5.6%±9.6)<br>*Fusobacteria:* 0.0-13.3 (1.8%±3.0)			

[a]Account for 99.4%; 91.9%; 86.1%; 74.9%; 76.6%; 53.3% of the distribution of Phylum, Class, Order, Family, Genus and Specie, respectively.

[b]*p*-value: Unpaired *t* test; 95% confidence interval. In bold, statically significant result.
